# 4*H*-Benzo[*d*][1,3]oxazin-4-ones and Dihydro Analogs from Substituted Anthranilic Acids and Orthoesters

**DOI:** 10.3390/molecules24193555

**Published:** 2019-10-01

**Authors:** Joel K. Annor-Gyamfi, Richard A. Bunce

**Affiliations:** Department of Chemistry, Oklahoma State University, Stillwater, OK 74078-3071, USA; jannorg@okstate.edu

**Keywords:** 4*H*-benzo*[d]*[1,3]oxazin-4-ones, 1,2-dihydro-4*H*-benzo*[d]*[1,3]oxazin-4-ones, heterocyclization, anthranilic acid, orthoesters

## Abstract

A one-pot route to 2-alkyl and 2-aryl-4*H*-benzo[*d*][1,3]oxazin-4-ones (also known as 4*H*-3,1-benzoxazin-4-ones) has been developed and studied. The method involves the reaction of aryl-substituted anthranilic acids with orthoesters in ethanol catalyzed by acetic acid. Additionally, we have also investigated the reaction under microwave conditions. Not all of the substrates were successful in yielding the target heterocycles as some of the reactions failed to undergo the final elimination. This process led to the isolation of (±)-2-alkyl/aryl-2-ethoxy-1,2-dihydro-4*H*-benzo*[d]*[1,3]oxazin-4-ones. The formation of the dihydro analogs correlated with the electron density on the aromatic ring: Electron-donating groups favored the 4*H*- benzo*[d]*[1,3]oxazin-4-ones, while electron-withdrawing groups tended to favor the dihydro product. Substituting a pyridine ring for the benzene ring in the substrate acid suppressed the reaction.

## 1. Introduction

**Figure 1:**
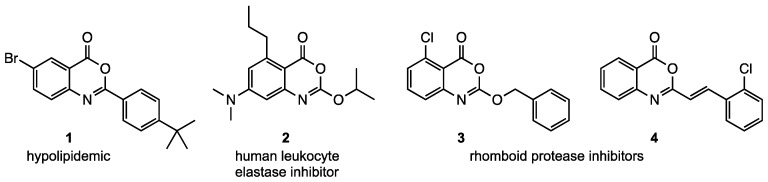
4*H*-Benzo[*d*][1,3]oxazin-4-ones with potential drug activity.

We recently reported the high-yield synthesis of quinazolin-4(3*H*)-ones by reaction of 2-aminobenzamides with orthoesters in ethanol promoted by acetic acid [1]. These heterocycles have received considerable attention as they have demonstrated diverse biological activities. With the success of this previous study, we sought to evaluate a similar strategy for the synthesis of 2-alkyl and 2-aryl-4*H*-benzo[*d*][1,3]oxazin-4-ones (also known as 4*H*-3,1-benzoxazin-4-ones). These compounds are less stable, and thus have been less well studied. An early account noted that several substituted structures related to compound **1** exhibited hypolipidemic activity [2]. Other benzoxazinones demonstrated potential as protease inhibitors. In particular, 4*H*-benzo[*d*][1,3]oxazin-4-one **2** was shown to have substrate inhibitory activity towards the serine protease human leukocyte elastase which is the presumed tissue degenerating agent in the pathogenesis of several diseases [3]. Two additional studies revealed that **3** and **4** interfered with the action of intramembrane serine proteases known as rhomboids that are important in the progression of diseases such as cancer, diabetes, invasion of apicomplexan parasites, and Parkinson’s disease [4,5]. Thus, access to these drug candidates would allow further study of the etiology of these diseases and could lead to new drugs to treat them. Structures **1**–**4** are shown in Figure 1. Apart from investigations of their potential use as drugs, 4*H*-benzo*[d]*[1,3]oxazin-4-ones have also been used as precursors to new heterocycles [6] as well as interesting peptide based oligomers [7].

Classic approaches to 4*H*-benzo*[d]*[1,3]oxazin-4-ones involved reaction of anthranilic acid with two equivalents of benzoyl chloride in pyridine to afford >90% of 2-aryl-4*H*-benzo*[d]*[1,3]oxazin-4-ones [2]. Similarly, treatment of substituted anthranilic acids with one equivalent of various acid chlorides to give the 2-amidobenzoic acid, followed by boiling in acetic anhydride, also afforded these products in good yields [8,9]. A later synthesis of 4*H*-benzo[*d*][1,3]oxazin-4-ones described a high-yield palladium-catalyzed cyclocarbonylation of *o*-iodoanilines with acid chlorides [10]. Furthermore, three oxidative approaches to these aryl-fused heterocycles have also been reported. In the first example, copper was used to promote the conversion of 2-alkynylanilines to 2-aryl-4*H*-benzo*[d]*[1,3]oxazin-4-ones using base conditions under oxygen atmosphere [11]. Another approach utilized oxone in nitromethane at 100 °C to accomplish the oxidation of 2-arylindoles to these same targets. A third oxidative procedure involved the water assisted oxidation of 2-arylindoles to 2-aryl-4*H*-benzo[*d*][1,3]oxazin-4-ones using (diacetoxyiodo)benzene [12]. Finally, a method related to the current procedure was reported for a small series of compounds substituted by phenyl at C2. This procedure utilized microwave energy to promote the formation of 2-phenyl-4*H*-benzo*[d]*[1,3]oxazin-4-ones from four anthranilic acids and triethyl orthobenzoate [13]. The yields varied in this reaction, but no problems were noted. In the current study, we have attempted to expand the scope of this transformation, examining the reaction of additional aryl-substituted anthranilic acids with four orthoesters. The results of our study are detailed below.

## 2. Results and Discussion

**Scheme 1:**
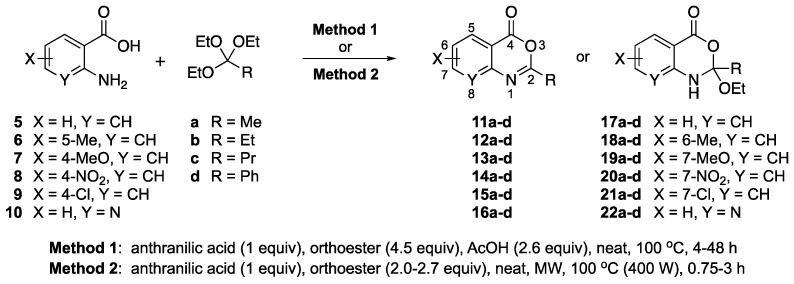
Formation of 4*H*-benzo*[d]*[1,3]oxazin-4-ones and their dihydro analogs from anthranilic acids and orthoesters.

Scheme 1 depicts the focus and scope of the current study. We have explored the synthesis of a range of 4*H*-benzo*[d]*[1,3]oxazin-4-ones from the reaction of six anthranilic acids with a range of commercially available orthoesters (Table 1 and Table 2). The reaction was run under two sets of conditions: one under thermal conditions promoted by acid, and the other under microwave conditions. The acid promoted conditions involved heating anthranilic acid (1 equivalent) with the orthoester (4.5 equivalents) in the presence of AcOH (2.6 equivalents), neat, at 100 °C, for 4–48 h. The microwave reaction was run using anthranilic acid (1 equivalent) and the orthoester (2.0–2.7 equivalents), neat, without AcOH, at 100 °C (400 W), for 0.75–3 h. Upon cooling, the products from both procedures crystallized from the crude reaction mixtures and were purified by trituration from 5% pentane in ether.

Contrary to what has been reported in related studies to date, we found that not all of the thermal reactions produced the target heterocycles cleanly. In several cases, (±)-2-substituted-1,2-dihydro-4*H*-benzo*[d]*[1,3]oxazin-4-ones, resulting from ring closure without elimination of the final molecule of ethanol, were isolated. This led us to expand our study to evaluate microwave reactions in addition to the standard acid-promoted thermal reactions; however, both protocols afforded the intermediate dihydro compounds from specific substrates. In several cases, it was possible to isolate either product by modification of the procedure. For example, the reaction of anthranilic acid (**5**) with triethyl orthobenzoate (**d**) for 24 h under thermal conditions afforded dihydro product **17d**, while extending the reaction to 48 h yielded 4*H*-benzo*[d]*[1,3]oxazin-4-one **11d**. In several other examples, however, it was not possible to induce the final elimination reaction.

The outcome of the reaction appeared to correlate with the electron density in the aromatic ring of the anthranilic acid. For rings substituted with electron donating groups (OMe, Me) or hydrogen, the 4*H*-benzo*[d]*[1,3]oxazin-4-one product predominated. More electron-poor aromatic rings bearing a second electron-withdrawing group on the aromatic ring (NO_2_ and Cl), in addition to the ortho carbonyl group, showed a tendency to give the 1,2-dihydro-4*H*-benzo*[d]*[1,3]oxazin-4-one product. Changing the benzene ring of the anthranilic acid to an electron-deficient pyridine, as in 2-aminonicotinic acid (**10**), almost completely shut down the reaction.

In most cases, it was possible to isolate at least one of the products in pure form. The lone exception was the attempted conversion of **5** to **17c** with triethyl orthobutyrate (**c**), which gave an inseparable mixture of the benzoxazinone and dihydro product under all conditions. Attempts to drive the reaction to the benzoxazinone using 5 equivalents of AcOH or spiking the reaction with H_2_SO_4_ failed to alter the outcome, while treatment with base (K_2_CO_3_, EtOH, molecular sieves) resulted in ring opening to the *N*-acylanthranilic acid. Finally, in attempted reactions of 2-aminonicotinic acid (**10**), triethyl orthoacetate (**a**) proceeded to give dihydro product **21a**, but all others afforded only ethyl 2-aminonicotinate resulting from esterification of the substrate acid.

**Scheme 2:**
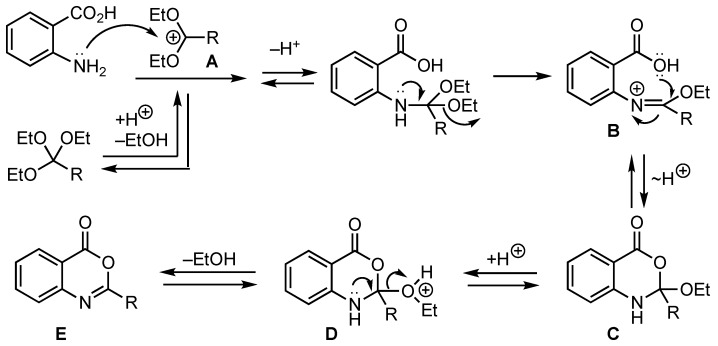
Presumed mechanism for formation of 4*H*-benzo*[d]*[1,3]oxazin-4-ones.

The presumed mechanism for the formation of 4*H*-benzo*[d]*[1,3]oxazin-4-ones is shown in Scheme 2. The process involves initial protonation of the orthoester and loss of ethanol to afford the stabilized carbocation **A**. Attack on **A** by the anthranilic acid amino group, proton exchange and loss of a second molecule of ethanol would then yield iminium ion **B**. Subsequent closure of the acid oxygen on the iminium carbon and proton exchange would give ring closed dihydro product **C**. Finally, protonation of **C** to give **D** and loss of ethanol would then generate the desired heterocycle **E**.

**Figure 2:**
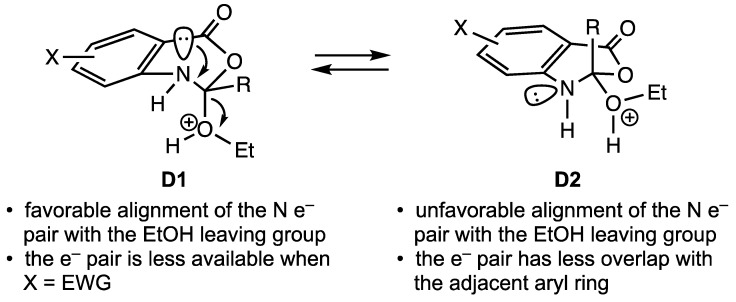
Conformational options for intermediate **D**.

Difficulty was noted in the final elimination of ethanol when the aromatic ring carried a second electron-withdrawing group (EWG) in addition to the adjacent ester carbonyl. For this elimination, the availability of the unshared electron pair on N1 and its ability to align antiperiplanar to the departing alcohol were assumed to be keys to the success of this elimination. The availability of these electrons, however, would be diminished in substrates with a strongly electron-deficient ring. Relatively little seems to be known about the necessity for the antiperiplanar alignment of the unshared pair for efficient elimination in this system. This anomeric effect has been extensively studied in acetals and hemiacetals [14], but much less so for their nitrogen analogs since elimination is normally facile. If achievable, conformation **D1** would result in good overlap for elimination of ethanol (see Figure 2). Placement of the C2 ethoxy group in a pseudo-axial position for this elimination should be favorable since this group is considered to be sterically smaller than an alkyl group [15,16]. However, this conformation also maximizes overlap of the N1 electron pair with the aromatic ring as well as the ortho carbonyl, and this could slow the elimination if the aromatic ring bears an additional electron-withdrawing substituent. The alternative conformation **D2** is not optimally aligned for elimination (see Figure 2). If the ring closure and protonation steps led to this conformation, conversion to **D1** could occur, but would likely be sluggish since flexibility in this rigid system is allowed only at the saturated atoms of the benzoxazinone.

## 3. Experimental Section

### 3.1. General

All commercial reagents and solvents were used as received. Unless otherwise indicated, all reactions were carried out under dry N_2_ in oven-dried glassware. Reactions were monitored by thin layer chromatography (TLC) using silica gel GF plates (Analtech No. 21521, Newark, DE, USA). Microwave reactions were performed in 2–5 mL microwave tubes (Biotage No. 354624, Charlotte, NC, USA) at 100 °C using a Biotage Initiator 2.5 system (Charlotte, NC, USA) at a power of 400 W from a magneton at 2.45 GHz. Melting points were obtained on a MEL-TEMP apparatus (Laboratory Devices, Cambridge, MA, USA), and were uncorrected. FT-IR spectra were run using a Varian Scimitar FTS 800 spectrophotometer (Randolph, MA, USA) as thin films or nujol mulls on NaCl disks. ^1^H- and ^13^C-NMR spectra were measured using a Bruker Avance 400 system (Billerica, MA, USA) in the indicated solvents (Cambridge Isotope, Andover, MA, USA) at 400 MHz and 101 MHz, respectively, with (CH_3_)_4_Si as the internal standard; coupling constants (*J*) are given in Hz. Copies of NMR spectra for the products are included in the Supplementary Information. High-resolution mass spectra (HRMS-ESI) were obtained using a Thermo LTQ-Orbitrap XL mass spectrometer (Thermo Scientific, Waltham, MA, USA).

### 3.2. Representative Procedure under Thermal Conditions. Method 1

The anthranilic acid (1.0 mmol) and the orthoester (4.5 mmol) were placed in a 15-mL Chemglass screw-cap pressure tube (CG-1880-01, Vineland, NJ, USA). Glacial acetic acid (2.6 mmol) was added, N_2_ was introduced to the vessel and the cap was tightened. The vessel was heated at 100 °C under neat conditions for 4–48 h, and then cooled to room temperature. Upon cooling, the crude product crystallized from the reaction mixture and was purified by trituration from 5% ether in pentane.

### 3.3. Representative Procedure under Microwave Conditions. Method 2

A solution of the anthranilic acid (1.0 mmol) in the orthoester (0.45 mL, 2.0–2.7 equivalents) was prepared in a 5-mL microwave tube and stirred for 30 s prior to irradiation at the "high absorption" setting. The reaction was performed under N_2_ at 100 °C (400 W) for 0.75–3 h. Upon cooling, the crude product crystallized from the reaction mixture and was purified by trituration from 5% ether in pentane.

The following compounds were prepared:

*2-Methyl-4H-benzo[d]*[1,3]*oxazin-4-one* (**11a**): 130 mg (81%, method 1) and 132 mg (82%, method 2) as white crystals, m.p. 79–80 °C (ref. [8] 80–81 °C); IR (thin film): 1700, 1624 cm^−1^; ^1^H-NMR (400 MHz, CDCl_3_): δ 8.29 (dd, 1H, *J* = 7.5, 1.6 Hz, ArH), 7.78 (ddd, 1H, *J* = 8.4, 7.1, 1.6 Hz, ArH), 7.69 (d, 1H, *J* = 8.4 Hz, ArH), 7.48 (ddd, 1H, *J* = 8.1, 7.1, 1.2 Hz, ArH), 2.60 (s, 3H, CH_3_); ^13^C-NMR (101 MHz, CDCl_3_): δ 164.1, 153.2, 149.4, 134.9, 127.0, 126.5, 126.3, 120.3, 22.2; HRMS (ESI): *m/z* [M + H]^+^ calcd. for C_9_H_7_NO_2_: 162.0555, found: 162.0553.

*2-Ethyl-4H-benzo[d]*[1,3]*oxazin-4-one* (**11b**): 140 mg (80%, method 1) and 145 mg (83%, method 2) as white crystals, m.p. 83–84 °C (ref. [8] m.p. 85–86 ^o^C); IR (thin film): 1750, 1645 cm^−1^; ^1^H-NMR (400 MHz, CDCl_3_): δ 8.20 (dd, 1H, *J* = 7.9, 1.5 Hz, ArH), 7.80 (ddd, 1H, *J* = 8.4, 7.5, 1.5 Hz, ArH), 7.57 (d, 1, *J* = 8.4 Hz, ArH), 7.50 (ddd, 1H, *J* = 8.2, 7.5, 1.2 Hz, ArH), 2.73 (q, 2H, *J* = 7.5 Hz, CH_2_CH_3_), 1.37 (t, 3H, *J* = 7.5 Hz, CH_2_CH_3_); ^13^C-NMR (101 MHz, CDCl_3_): δ 163.9, 159.9, 146.5, 136.5, 128.4, 128.1, 126.6, 116.9, 28.2, 10.3; HRMS (ESI): *m/z* [M + H]^+^ calcd. for C_10_H_9_NO_2_: 176.0712, found: 176.0713.

*2-Phenyl-4H-benzo[d]*[1,3]*oxazin-4-one* (**11d**): 174 mg (78%, method 1) and 179 mg (80%, method 2) as off-white crystals, m.p. 121–122 °C (ref. [8] m.p. 123 °C); IR (thin film): 1762, 1615 cm^−1^; ^1^H-NMR (400 MHz, CDCl_3_): δ 8.34-8.30 (complex, 2H, ArH), 8.25 (dd, 1H, *J* = 7.9, 1.5, ArH), 7.84 (ddd, 1H, *J* = 8.1, 7.3, 1.5 Hz, ArH), 7.70 (d, *J* = 8.1 Hz, ArH), 7.62-7.48 (complex, 4H, ArH); ^13^C-NMR (101 MHz, CDCl_3_): δ 159.6, 157.1, 147.0, 136.6, 132.6, 130.3, 128.8, 128.6, 128.32, 128.26, 127.2, 117.0; HRMS (ESI): *m/z* [M + H]^+^ calcd. for C_14_H_9_NO_2_: 224.0712, found: 224.0709.

*2,6-Dimethyl-4H-benzo[d]*[1,3]*oxazin-4-one* (**12a**): 119 mg (68%, method 2) as off-white crystals, m.p. 122–123 °C; IR (thin film): 1682, 1595 cm^−1^; ^1^H-NMR (400 MHz, CDCl_3_): δ 7.98 (s, 1H, ArH), 7.61 (d, 1H, *J* = 8.2 Hz, ArH), 7.44 (d, 1H, *J* = 8.2 Hz, ArH), 2.47 (s, 3H, CH_3_), 2.45 (s, 3H, CH_3_); ^13^C-NMR (101 MHz, CDCl_3_): δ 159.9, 159.4, 144.3, 138.5, 137.7, 128.0, 126.2, 116.3, 21.3, 21.2; HRMS (ESI): *m/z* [M + H]^+^ calcd. for C_10_H_9_NO_2_: 176.0712, found: 176.0709.

*2-Ethyl-6-methyl-4H-benzo[d]*[1,3]*oxazin-4-one* (**12b**): 125 mg (66%, method 2) as white crystals, m.p. 103–104 °C; IR (thin film): 1686, 1596 cm^−1^; ^1^H-NMR (400 MHz, CDCl_3_): δ 7.98 (s, 1H, ArH), 7.60 (d, 1H, *J* = 8.2 Hz, ArH), 7.46 (d, 1H, *J* = 8.2 Hz, ArH), 2.71 (q, 2H, *J* = 7.5 Hz, *CH_2_*CH_3_), 2.47 (s, 3H, ArCH_3_), 1.36 (t, 3H, *J* = 7.5 Hz, CH_2_*CH_3_*); ^13^C-NMR (101 MHz, CDCl_3_): δ 163.1, 160.1, 144.3, 138.4, 137.7, 128.0, 126.4, 116.5, 26.1, 21.2, 10.3; HRMS (ESI): *m/z* [M + H]^+^ calcd. for C_11_H_11_NO_2_: 190.0868, found: 190.0865.

*6-Methyl-2-phenyl-4H-benzo[d]*[1,3]*oxazin-4-one* (**12d**): 202 mg (85%, method 2) as off-white crystals, m.p. 144–145 °C; IR (thin film): 1752, 1607 cm^−1^; ^1^H-NMR (400 MHz, CDCl_3_): δ 8.29 (d, 2H, *J* = 8.2 Hz, ArH), 8.04 (s, 1H, ArH), 7.66-7.47 (complex, 5H, ArH), 2.49 (s, 3H, ArCH_3_); ^13^C-NMR (101 MHz, CDCl_3_): δ 159.8, 156.4, 144.8, 138.7, 137.8, 132.4, 130.4, 128.7, 128.19, 128.17, 127.0, 116.7, 21.2; HRMS (ESI): *m/z* [M + H]^+^ for C_15_H_11_NO_2_: 238.0868, found: 238.0870.

*7-Methoxy-2-methyl-4H-benzo[d]*[1,3]*oxazin-4-one* (**13a**): 166 mg (87%, method 1) and 168 mg (88%, method 2) as light yellow crystals, m.p. 119–120 °C; IR (nujol): 2830, 1747, 1610 cm^−1^; ^1^H-NMR (400 MHz, DMSO-*d_6_*): δ 8.00 (d, 1H, *J* = 8.8 Hz, ArH), 7.14 (dd, 1H, *J* = 8.8, 2.5 Hz, ArH), 7.05 (d, 1H, *J* = 2.5 Hz, ArH), 3.91 (s, 3H, OCH_3_), 2.38 (s, 3H, CH_3_); ^13^C-NMR (101 MHz, DMSO-*d_6_*): δ 166.3, 161.5, 159.2, 149.0, 130.2, 117.1, 109.5, 109.1, 56.5, 21.5; HRMS (ESI): *m/z* [M + H]^+^ calcd. for C_10_H_9_NO_3_: 192.0661, found: 192.0659.

*2-Ethyl-7-methoxy-4H-benzo[d]*[1,3]*oxazin-4-one* (**13b**): 176 mg (86%, method 1) and 181 mg (88%, method 2) as light yellow crystals, m.p. 89–90 °C; IR (nujol): 2840, 1767, 1644, 1609 cm^−1^; ^1^H-NMR (400 MHz, DMSO-*d_6_*): δ 7.99 (d, 1H, *J* = 8.8 Hz, ArH) 7.14 (dd, 1H, *J* = 8.8, 2.5 Hz, ArH), 7.07 (d, 1H, *J* = 2.5 Hz, ArH), 3.92 (s, 3H, OCH_3_), 2.68 (q, 2H, *J* = 7.5 Hz, *CH_2_*CH_3_), 1.24 (t, 3H, *J* = 7.5 Hz, CH_2_*CH_3_*); ^13^C-NMR (101 MHz, DMSO-*d_6_*): δ 166.3, 164.9, 159.2, 149.0, 130.1, 117.3, 109.7, 109.1, 56.6, 27.7, 10.2; HRMS (ESI): *m/z* [M + H]^+^ calcd. for C_11_H_11_NO_3_: 206.0817, found: 206.0817.

*7-Methoxy-2-propyl-4H-benzo[d]*[1,3]*oxazin-4-one* (**13c**): 189 mg (86%, method 1) and 191 mg (87%, method 2) as light yellow crystals, m.p. 77–78 °C; IR (nujol): 2841, 1766, 1643, 1609 cm^−1^; ^1^H-NMR (400 MHz, DMSO-*d_6_*): δ 8.00 (d, 1H, *J* = 8.8 Hz, ArH), 7.14 (dd, 1H, *J* = 8.8, 2.5 Hz, ArH), 7.07 (d, 1H, *J* = 2.5 Hz, ArH), 3.92 (s, 3H, OCH_3_), 2.63 (t, 2H, *J* = 7.4 Hz, *CH_2_*CH_2_CH_3_), 1.75 (sextet, 2H, *J* = 7.4 Hz, CH_2_*CH_2_*CH_3_); 0.98 (t, 3H, *J* = 7.4 Hz, CH_2_CH_2_*CH_3_*); ^13^C-NMR (101 MHz, DMSO-*d_6_*): δ 166.3, 163.9, 159.2, 148.9, 130.1, 117.3, 109.7, 109.1, 56.6, 36.2, 19.2, 13.9; HRMS (ESI): *m/z* [M + H]^+^ calcd. for C_12_H_13_NO_3_: 220.0974, found: 220.0971.

*7-Methoxy-2-phenyl-4H-benzo[d]*[1,3]*oxazin-4-one* (**13d**): 210 mg (83%, method 1) and 215 mg (85%, method 2) as light yellow crystals, m.p. 149–150 °C; IR (nujol): 2842, 1744, 1607 cm^−1^; ^1^H-NMR (400 MHz, DMSO-*d_6_*): δ 8.17 (d, 2H, *J* = 8.1 Hz, ArH), 8.03 (d, 1H, *J* = 9.4 Hz, ArH), 7.67 (d, 1H, *J* = 7.3 Hz, ArH), 7.59 (t, 2H, *J* = 8.1 Hz, ArH), 7.15 (m, 2H, ArH), 3.94 (s, 3H, OCH_3_); ^13^C-NMR (101 MHz, DMSO-*d_6_*): δ 166.4, 158.8, 157.6, 149.2, 133.2, 130.5, 130.3, 129.4, 128.3, 117.6, 109.9, 109.6, 56.6; HRMS (ESI): *m/z* [M + H]^+^ calcd. for C_15_H_11_NO_3_: 254.0817, found: 254.0814.

*7-Nitro-2-phenyl-4H-benzo[d]*[1,3]*oxazin-4-one* (**14d**): 244 mg (91%, method 2) as light orange crystals, m.p. 175–176 °C; IR (thin film): 1757, 1629, 1608, 1530, 1349 cm^−1^; ^1^H-NMR (400 MHz, CDCl_3_): δ 8.52 (d, 1H, *J* = 2.2 Hz, ArH), 8.41 (d, 1H, *J* = 8.6 Hz, ArH), 8.33 (d, 2H, *J* = 7.4 Hz, ArH), 8.29 (dd, 1H, *J* = 8.6, 2.2 Hz, ArH), 7.64 (t, 1H, *J* = 7.4 Hz, ArH), 7.55 (t, 2H, *J* = 7.4 Hz, ArH); ^13^C-NMR (101 MHz, CDCl_3_): δ 159.0, 157.9, 152.9, 148.1, 133.6, 130.4, 129.3, 129.0, 128.7, 122.5, 122.0, 121.3; HRMS (ESI): *m/z* [M + H]^+^ calcd. for C_14_H_8_N_2_O_4_: 269.0562, found: 269.0558.

*7-Chloro-2-methyl-4H-benzo[d]*[1,3]*oxazin-4-one* (**15a**): 166 mg (85%, method 2) as white crystals, m.p. 149–150 °C; IR (thin film): 1762, 1696, 1642, 1596 cm^−1^; ^1^H-NMR (400 MHz, CDCl_3_): δ 8.11 (d, 1H, *J* = 8.4 Hz, ArH), 7.54 (d, 1H, *J* = 2.0 Hz, ArH), 7.46 (dd, 1H, *J* = 8.4, 2.0 Hz, ArH), 2.47 (s, 3H CH_3_); ^13^C-NMR (101 MHz, CDCl_3_): δ 161.6, 158.9, 147.5, 142.9, 129.7, 128.8, 126.3, 115.1, 21.4; HRMS (ESI): *m/z* [M + H]^+^ calcd. for C_9_H_6_^35^ClNO_2_: 196.0165, found: 196.0166.

*7-Chloro-2-phenyl-4H-benzo[d]*[1,3]*oxazin-4-one* (**15d**): 237 mg (92%, method 2) as off-white crystals, m.p. 188–189 °C; IR (thin film): 1760, 1619, 1596 cm^−1^; ^1^H-NMR (400 MHz, CDCl_3_): δ 8.31 (d, 2H, *J* = 7.4 Hz, ArH), 8.17 (d, 1H, *J* = 8.4 Hz, ArH), 7.70 (d, 1H, *J* = 2.0 Hz, ArH), 7.60 (tt, 1H, *J* = 7.4, 1.4 Hz, ArH), 7.52 (t, 2H, *J* = 7.4 Hz, ArH), 7.48 (dd, 1H, *J* = 8.4, 2.0 Hz, ArH); ^13^C-NMR (101 MHz, CDCl_3_): δ 158.8, 158.3, 148.1, 143.0, 133.0, 129.9, 129.86, 128.83, 128.79, 128.5, 127.0, 115.4; HRMS (ESI): *m/z* [M + H]^+^ calcd. for C_14_H_8_^35^ClNO_2_: 258.0322, found: 258.0317.

*(±)-2-Ethoxy-2-phenyl-1,2-dihydro-4H-benzo[d]*[1,3]*oxazin-4-one* (**17d**): 232 mg (86%, method 1) as white crystals, m.p. 92–93 °C; IR (thin film): 3254, 1668, 1595 cm^−1^; ^1^H-NMR (400 MHz, CDCl_3_): δ 12.09 (br s, 1H, NH), 8.94 (dd, 1H, *J* = 8.5, 1.1 Hz), ArH), 8.1 (dd, 1H, *J* = 8.0, 1.7 Hz, ArH), 8.06 (dd, 2H, *J* = 7.8, 1.5 Hz, ArH), 7.64-7.49 (complex, 4H, ArH), 7.13 (td, 1H, *J* = 7.4, 1.2 Hz, ArH), 4.43 (q, 2H, *J* = 7.1 Hz, O*CH_2_*CH_3_), 1.43 (t, 3H, *J* = 7.1 Hz, OCH_2_*CH_3_*); ^13^C-NMR (101 MHz, CDCl_3_): δ 168.6, 165.4, 141.9, 134.9, 134.7, 131.9, 130.9, 128.8, 127.4, 122.6, 120.5, 115.5, 61.5, 14.2; HRMS (ESI): *m/z* [M + H]^+^ calcd. for C_16_H_15_NO_2_: 254.1181, found: 254.1177.

*(±)-2-Ethoxy-2,6-dimethyl-1,2-dihydro-4H-benzo[d]*[1,3]*oxazin-4-one* (**18a**): 195 mg (88%, method 1) as white crystals, m.p. 110–111 °C; IR (thin film): 3266, 1677, 1595 cm^−1^; ^1^H-NMR (400 MHz, CDCl_3_): δ 10.97 (br s, 1H, NH), 8.57 (d, 1H, *J* = 8.6 Hz, ArH), 7.83 (s, 1H, ArH), 7.34 (d, 1H, *J* = 8.6 Hz, ArH), 4.37 (q, 2H, *J* = 7.1 Hz, O*CH_2_*CH_3_), 2.33 (s, 3H, ArCH_3_), 2.22 (s, 3H, CH_3_), 1.42 (t, 3H, *J* = 7.1 Hz, OCH_2_*CH_3_*); ^13^C-NMR (101 MHz, CDCl_3_): δ 168.9, 168.4, 139.2, 135.3, 131.9, 130.8, 120.3, 115.0, 61.3, 25.5, 20.7, 14.2; HRMS (ESI): *m/z* [M + H]^+^ calcd. for C_12_H_15_NO_3_: 222.1130, found: 222.1128.

*(±)2-Ethoxy-2-ethyl-6-methyl-1,2-dihydro-4H-benzo[d]*[1,3]*oxazin-4-one* (**18b**): 205 mg (87%, method 1) as white crystals, m.p. 78–79 °C; IR (thin film): 3261, 1677, 1596 cm^−1^; ^1^H-NMR (400 MHz, CDCl_3_): δ 11.0 (br s, 1H, NH), 8.62 (d, 1H, *J* = 8.6 Hz, ArH), 7.83 (s, 1H, ArH), 7.34 (d, 1H, *J* = 8.6 Hz, ArH), 4.37 (q, 2H, *J* = 7.0 Hz, O*CH_2_*CH_3_), 2.46 (q, 2H, *J* = 7.5 Hz, *CH_2_*CH_3_), 2.33 (s, 3H, ArCH_3_), 1.42 (t, 3H, *J* = 7.0 Hz, OCH_2_*CH_3_*), 1.27 (t, 3H, *J* = 7.5 Hz, CH_2_*CH_3_*); ^13^C-NMR (101 MHz, CDCl_3_): δ 172.7, 168.4, 139.4, 135.3, 131.7, 130.8, 120.3, 114.9, 61.3, 31.7, 20.7, 14.2, 9.7; HRMS (ESI): *m/z* [M + H]^+^ calcd. for C_13_H_17_NO_3_: 236.1287, found: 236.1284.

*(±)-2-Ethoxy-6-methyl-2-propyl-1,2-dihydro-4H-benzo[d]*[1,3]*oxazin-4-one* (**18c**): 219 mg (88%, method 1) and 222 mg (89%, method 2) as off-white crystals, m.p. 39–40 °C; IR (thin film): 3265, 1682, 1599 cm^−1^; ^1^H-NMR (400 MHz, CDCl_3_): δ 10.98 (br s, 1H, NH), 8.62 (d, 1H, *J* = 8.6 Hz, ArH), 7.83 (s, 1H, ArH), 7.35 (d, 1H, *J* = 8.6 Hz, ArH), 4.38 (q, 2H, *J* = 7.1 Hz, O*CH_2_*CH_3_), 2.41 (q, 2H, *J* = 7.5 Hz, *CH_2_*CH_2_CH_3_), 2.33 (s, 3H, ArCH_3_), 1.78 (sextet, 2H, *J* = 7.5 Hz, CH_2_*CH_2_*CH_3_), 1.42 (t, 3H, *J* = 7.1 Hz, OCH_2_*CH_3_*), 1.01 (t, 3H, *J* = 7.5 Hz, CH_2_CH_2_*CH_3_*); ^13^C-NMR (101 MHz, CDCl_3_): δ 171.9, 168.4, 139.3, 135.3, 131.7, 130.8, 120.3, 114.9, 61.3, 40.6, 20.7, 19.0, 14.2, 13.8; HRMS (ESI): *m/z* [M + H]^+^ calcd. for C_14_H_19_NO_3_: 250.1443, found: 250.1441.

*(±)-2-Ethoxy-6-methyl-2-phenyl-1,2-dihydro-4H-benzo[d]*[1,3]*oxazin-4-one* (**18d**): 249 mg (88%, method 1) as white crystals, m.p. 117–118 °C; IR (thin film): 3249, 1662, 1602 cm^−1^; ^1^H-NMR (400 MHz, CDCl_3_): δ 11.98 (br s, 1H, NH), 8.82 (d, 1H, *J* = 8.5 Hz, ArH), 8.05 (d, 2H, *J* = 6.8 Hz, ArH), 7.89 (s, 1H, ArH), 7.58-7.47 (complex, 3H), 7.42 (d, 1H, *J* = 8.5 Hz, ArH), 4.42 (q, 2H, *J* = 7.1 Hz, O*CH_2_*CH_3_), 3.36 (s, 3H, ArCH_3_), 1.44 (q, 3H, *J* = 7.1 Hz, OCH_2_*CH_3_*); ^13^C-NMR (101 MHz, CDCl_3_): δ 168.9, 165.5, 139.5, 135.4, 135.1, 132.1, 131.8, 131.0, 128.8, 127.3, 120.4, 115.4, 61.5, 20.8, 14.3; HRMS (ESI): *m/z* [M + H]^+^ calcd. for C_17_H_17_NO_3_: 284.1287, found: 284.1283.

*(±)-2-Ethoxy-2-methyl-7-nitro-1,2-dihydro-4H-benzo[d]*[1,3]*oxazin-4-one* (**20a**): 212 mg (84%, method 1) and 219 mg (87%, method 2) as light tan crystals, m.p. 108–109 °C; IR (thin film): 3270, 1702, 1604, 1538, 1350 cm^−1^; ^1^H-NMR (400 MHz, CDCl_3_): δ 11.16 (br s, 1H, NH), 9.60 (d, 1H, *J* = 2.4 Hz, ArH), 8.20 (d, 1H, *J* = 8.8 Hz, ArH), 7.87 (dd, 1H, *J* = 8.8, 2.4 Hz, ArH), 4.45 (q, 2H, *J* = 7.1 Hz, O*CH_2_*CH_3_), 2.28 (s, 3H, CH_3_), 1.46 (t, 3H, *J* = 7.1 Hz, OCH_2_*CH_3_*); ^13^C-NMR (101 MHz, CDCl_3_): δ 169.2, 166.9, 151.2, 142.2, 131.9, 119.3, 116.4, 115.2, 62.5, 25.5, 14.1; HRMS (ESI): *m/z* [M + H]^+^ calcd. for C_11_H_12_N_2_O_5_: 253.0825, found: 253.0824.

*(±)-2-Ethoxy-2-ethyl-7-nitro-1,2-dihydro-4H-benzo[d]*[1,3]*oxazin-4-one* (**20b**): 229 mg (86%, method 1) and 234 mg (88%, method 2) as light tan crystals, m.p. 74–75 °C; IR (thin film): 3273, 1694, 1600, 1538, 1349 cm^−1^; ^1^H-NMR (400 MHz, CDCl_3_): δ 11.18 (br s, 1H, NH), 9.65 (d, 1H, *J* = 2.4 Hz, ArH), 8.20 (d, 1H, *J* = 8.8 Hz, ArH), 7.86 (dd, 1H, *J* = 8.8, 2.4 Hz, ArH), 4.45 (q, 2H, *J* = 7.1 Hz, O*CH_2_*CH_3_), 2.53 (q, 2H, *J* = 7.5 Hz, *CH_2_*CH_3_), 1.46 (t, 3H, *J* = 7.1 Hz, OCH_2_*CH_3_*), 1.30 (t, 3H, *J* = 7.5 Hz, CH_2_*CH_3_*); ^13^C-NMR (101 MHz, CDCl_3_): δ 173.1, 166.9, 151.2, 142.5, 131.9, 119.3, 116.3, 115.3, 62.4, 31.6, 14.1, 9.3; HRMS (ESI): *m/z* [M + H]^+^ calcd. for C_12_H_14_N_2_O_5_: 267.0981, found: 267.0977.

*(±)-2-Ethoxy-2-propyl-7-nitro-1,2-dihydro-4H-benzo[d]*[1,3]*oxazin-4-one* (**20c**): 238 mg (85%, method 1) and 245 mg (88%, method 2) as light tan crystals, m.p. 66–67 °C; IR (thin film): 3270, 1697, 1604, 1538, 1349 cm^−1^; ^1^H-NMR (400 MHz, CDCl_3_): δ 11.17 (br s, 1H, NH), 9.65 (d, 1H, *J* = 2.4 Hz, ArH), 8.20 (d, 1H, *J* = 8.8 Hz, ArH), 7.86 (dd, 1H, *J* = 8.8, 2.4 Hz, ArH), 4.45 (q, 2H, *J* = 7.1 Hz, O*CH_2_*CH_3_), 2.47 (q, 2H, *J* = 7.4 Hz, *CH_2_*CH_2_CH_3_), 1.81 (sextet, 2H, *J* = 7.4 Hz, CH_2_*CH_2_*CH_3_), 1.46 (t, 3H, *J* = 7.1 Hz, OCH_2_*CH_3_*), 1.04 (t, 3H, *J* = 7.4 Hz, CH_2_CH_2_*CH_3_*); ^13^C-NMR (101 MHz, CDCl_3_): δ 172.3, 166.9, 151.2, 142.5, 131.9, 119.3, 116.3, 115.3, 62.4, 40.4, 18.8, 14.1, 13.7; HRMS (ESI): *m/z* [M + H]^+^ calcd. for C_13_H_16_N_2_O_5_: 281.1138, found: 281.1140.

*(±)-2-Ethoxy-7-nitro-2-phenyl-1,2-dihydro-4H-benzo[d]*[1,3]*oxazin-4-one* (**20d**): 283 mg (90%, method 1) as off-white crystals, m.p. 180–181 °C; IR (thin film): 3257, 1673, 1651, 1600, 1538, 1346 cm^−1^; ^1^H-NMR (400 MHz, CDCl_3_): δ 11.62 (br s, 1H, NH), 9.31 (d, 1H, *J* = 2.4 Hz, ArH), 8.20 (d, 1H, *J* = 8.8 Hz, ArH), 8.03 (dd, 1H, *J* = 8.8, 2.4 Hz, ArH), 7.97 (d, 2H, *J* = 7.4 Hz, ArH, 7.69 (t, 1H, *J* = 7.4 Hz, ArH), 7.61 (t, 2H, *J* = 7.4 Hz, ArH), 4.38 (q, 2H, *J* = 7.1 Hz, O*CH_2_*CH_3_), 1.33 (t, 3H, *J* = 7.1 Hz, OCH_2_*CH_3_*); ^13^C-NMR (101 MHz, CDCl_3_): δ 166.7, 165.7, 150.6, 141.0, 134.1, 133.1, 132.7, 129.5, 127.7, 1239, 118.2, 116.0, 62.6, 14.3; HRMS (ESI): *m/z* [M + H]^+^ calcd. for C_16_H_14_N_2_O_5_: 315.0981, found: 315.0975.

*(±)-7-Chloro-2-ethoxy-2-methyl-1,2-dihydro-4H-benzo[d]*[1,3]*oxazin-4-one* (**21a**): 203 mg (84%, method 1) as white crystals, m.p. 75–76 °C; IR (thin film): 3262, 1681, 1578 cm^−1^; ^1^H-NMR (400 MHz, CDCl_3_): δ 10.70 (br s, 1H, NH), 8.43 (d, 1H, *J* = 2.2 Hz, ArH), 7.92 (d, 1H, *J* = 8.6 Hz, ArH), 7.23 (dd, 1H, *J* = 8.6, 2.2 Hz, ArH), 4.83 (q, 2H, *J* = 7.1 Hz, O*CH_2_*CH_3_), 2.15 (s, 3H, CH_3_), 1.34 (t, 3H, *J* = 7.1 Hz, OCH_2_*CH_3_*); ^13^C-NMR (101 MHz, CDCl_3_): δ 169.1, 167.7, 142.4, 140.8, 131.8, 122.6, 120.1, 113.2, 61.6, 25.5, 14.2; HRMS (ESI): *m/z* [M + H]^+^ calcd. for C_11_H_12_^35^ClNO_3_: 242.0584, found: 242.0582.

*(±)-7-Chloro-2-ethoxy-2-ethyl-1,2-dihydro-4H-benzo[d]*[1,3]*oxazin-4-one* (**21b**): 215 mg (84%, method 1) and 220 mg (86%, method 2) as white crystals, m.p. 69–70 °C; IR (thin film): 3238, 1685, 1581 cm^−1^; ^1^H-NMR (400 MHz, CDCl_3_): δ 11.17 (br s, 1H, NH), 8.86 (d, 1H, *J* = 2.2 Hz, ArH), 7.96 (d, 1H, *J* = 8.6 Hz, ArH), 7.04 (dd, 1H, *J* = 8.6, 2.2 Hz, ArH), 4.38 (q, 2H, *J* = 7.1 Hz, O*CH_2_*CH_3_), 2.48 (q, 2H, *J* = 7.5 Hz, *CH_2_*CH_3_), 1.41 (t, 3H, *J* = 7.1 Hz, OCH_2_*CH_3_*), 1.27 (t, 3H, *J* = 7.5 Hz, CH_2_*CH_3_*); ^13^C-NMR (101 MHz, CDCl_3_): δ 173.0, 167.8, 142.6, 140.9, 131.8, 122.5, 120.2, 113.2, 61.6, 31.7, 14.2, 9.5; HRMS (ESI): *m/z* [M + H]^+^ calcd. for C_12_H_14_^35^ClNO_3_: 256.0741, found: 256.0743.

*(±)-7-Chloro-2-ethoxy-2-propyl-1,2-dihydro-4H-benzo[d]*[1,3]*oxazin-4-one* (**21c**): 224 mg (83%, method 1) and 227 mg (84%, method 2) as white semisolid, m.p. 25–26 °C; IR (thin film): 3280, 1691, 1581cm^−1^; ^1^H-NMR (400 MHz, CDCl_3_): δ 11.16 (br s, 1H, NH), 8.85 (s, 1H, ArH), 7.96 (d, 1H, *J* = 8.6 Hz, ArH), 7.04 (dd, 1H, *J* = 8.6, 2.2 Hz, ArH), 4.38 (q, 2H, *J* = 7.1 Hz, O*CH_2_*CH_3_), 2.42 (q, 2H, *J* = 7.4 Hz, *CH_2_*CH_2_CH_3_), 1.78 (sextet, 2H, *J* = 7.4 Hz, CH_2_*CH_2_*CH_3_), 1.42 (t, 3H, *J* = 7.1 Hz, OCH_2_*CH_3_*), 1.02 (t, 3H, *J* = 7.4 Hz, CH_2_CH_2_*CH_3_*); ^13^C-NMR (101 MHz, CDCl_3_): δ 172.2, 167.7, 142.5, 140.8, 131.8, 122.5, 120.2, 113.2, 61.6, 40.5, 18.9, 14.2, 13.2; HRMS (ESI): *m/z* [M + H]^+^ calcd. for C_13_H_16_^35^ClNO_3_: 270.0897, found: 270.0893.

*(±)-7-Chloro-2-ethoxy-2-phenyl-1,2-dihydro-4H-benzo[d]*[1,3]*oxazin-4-one* (**21d**): 258 mg (85%, method 1) as white crystals, m.p. 128–129 °C; IR (thin film): 3241, 1673, 1585 cm^−1^; ^1^H-NMR (400 MHz, CDCl_3_): δ 12.13 (br s, 1H, NH), 9.05 (d, 1H, *J* = 2.1 Hz, ArH), 8.03 (overlapping signals, apparent t, 3H, *J* ≈ 8.4 Hz, ArH), 7.60-7.49 (complex, 3H, ArH), 7.08 (dd, 1H, *J* = 8.6, 2.1 Hz, ArH), 4.42 (q, 2H, *J* = 7.1 Hz, O*CH_2_*CH_3_), 1.43 (t, 3H, *J* = 7.1 Hz, OCH_2_*CH_3_*); ^13^C-NMR (101 MHz, CDCl_3_): δ 168.1, 165.7, 142.7, 141.0, 134.4, 132.2, 132.0, 128.9, 127.4, 122.8, 120.3, 113.6, 61.8, 14.2; HRMS (ESI): *m/z* [M + H]^+^ calcd. for C_16_H_14_^35^ClNO_3_: 304.0741, found: 304.0737.

*(±)-2-Ethoxy-2-methyl-1,2-dihydro-4H-pyrido[2,3-d]*[1,3]*oxazin-4-one* (**22a**): 177 mg (85%, method 1) as white crystals, m.p. 55–56 °C; IR (thin film): 3270, 1680, 1589 cm^−1^; ^1^H-NMR (400 MHz, CDCl_3_): δ 10.79 (br s, 1H, NH), 8.59 (dd, 1H, *J* = 4.8, 2.0 Hz, PyH), 8.33 (dd, 1H, *J* = 7.9, 2.0 Hz, PyH), 7.06 (dd, 1H, *J* = 7.9, 4.8 Hz, PyH), 4.41 (q, 2H, *J* = 2.1 Hz, O*CH_2_*CH_3_), 2.42 (s, 3H, CH_3_), 1.42 (t, 3H, *J* = 7.1 Hz, OCH_2_*CH_3_*); ^13^C-NMR (101 MHz, CDCl_3_): δ 169.6, 166.6, 152.9, 152.5, 140.0, 118.0, 111.1, 62.0, 26.0, 14.2; HRMS (ESI): *m/z* [M + H]^+^ calcd. for C_10_H_12_N_2_O_3_: 209.0926, found: 209.0925.

## 4. Conclusions

We have studied the formation of 4*H*-benzo[*d*][1,3]oxazin-4-ones under thermal conditions with acetic acid as well as microwave conditions without acid. In most cases, the target heterocycle was formed in high yield. The reaction was performed with no solvent, and the desired products were isolated by crystallization and trituration. The microwave reaction proceeded much faster (0.75–3 h) than the thermal reactions (4–48 h) and did not require added acid. Thus, the microwave protocol represents the superior procedure. Stereoelectronic effects strongly impacted the outcome of the reaction. In addition to the benzoxazinone products, a dihydro intermediate resulting from failure to undergo the final elimination was observed in some substrates. The formation of the dihydro compound arose in reactants where the fused aromatic ring possessed a second electron-withdrawing group in addition to the conjugated carbonyl on the ortho carbon. This could deactivate the electron pair on N1 and slow the elimination process, even if the C2 ethoxy group is able to adopt the required pseudo-axial conformation. The formation of these dihydro systems provides access to a new substitution pattern in this family of compounds, and could yield additional candidates for biological testing. This finding coupled with the fact that orthoesters are easily prepared from nitriles [17,18] should provide a route to many members of this novel class of structures.

## Figures and Tables

**Table 1:** Formation of 4*H*-benzo*[d]*[1,3]oxazin-4-ones.

Substrate	Product	R	Method ^a^	Time (h)	Yield (%)
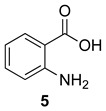	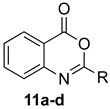	a: Me	1(2)	48(0.75)	81(82)
		b: Et	1(2)	7(0.75)	80(83)
		c: Pr	1(2)	4(0.75)	^b^
		d: Ph	1(2)	48(0.75)	78(80)
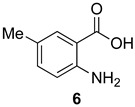	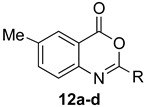	a: Me	2	0.75	68
		b: Et	2	0.75	66
		c: Pr	1(2)	24(0.75)	^c^
		d: Ph	2	0.75	85
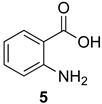	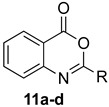	a: Me	1(2)	48(0.75)	81(82)
		b: Et	1(2)	7(0.75)	80(83)
		c: Pr	1(2)	4(0.75)	^b^
		d: Ph	1(2)	48(0.75)	78(80)
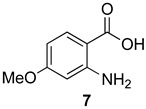	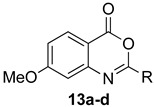	a: Me	1(2)	24(0.75)	87(88)
		b: Et	1(2)	24(0.75)	86(88)
		c: Pr	1(2)	24(0.75)	86(87)
		d: Ph	1(2)	48(0.75)	83(85)
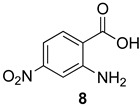	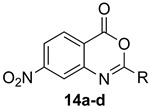	a: Me	1(2)	24(0.75)	^c^
		b: Et	1(2)	24(0.75)	^c^
		c: Pr	1(2)	24(0.75)	^c^
		d: Ph	2	3	91
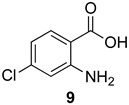	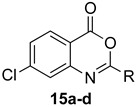	a: Me	2	0.75	85
		b: Et	1(2)	48(0.75)	^c^
		c: Pr	1(2)	48(0.75)	^c^
		d: Ph	2	1.5	92
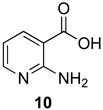	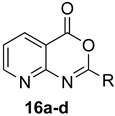	a: Me	1	24	^c^
		b: Et	2	0.75	^d^
		c: Pr	2	0.75	^d^
		d: Ph	2	0.75	^d^

^a^ For anthranilic acid (1 equivalent): Method 1: Orthoester (4.5 equivalents), AcOH (2.6 equivalents), neat, 100 °C, for the indicated time; Method 2: Orthoester (2.0–2.7 equivalents), neat, MW, 100 °C, for the indicated time; ^b^ An inseparable mixture of the 4*H*-benzo[*d*][1,3]oxazin-4-one and the dihydro product was formed; ^c^ The dihydro product formed; ^d^ Only ethyl 2-aminonicotinate was formed from esterification of the substrate acid.

**Table 2:** Formation of (±)-1,2-dihydro-4*H*-benzo*[d]*[1,3]oxazin-4-ones.

Substrate	Product	R	Method ^a^	Time (h)	Yield (%)
**5**	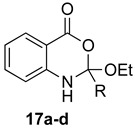	**a:** Me			^b^
		**b:** Et			^b^
		**c:** Pr			^c^
		**d:** Ph	1	24	86
**6**	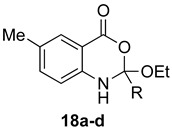	**a:** Me	1	24	88
		**b:** Et	1	24(0.75)	87
		**c:** Pr	1(2)	24	88(89)
		**d:** Ph	1	48	89
**7**	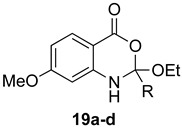	**a:** Me			^b^
		**b:** Et			^b^
		**c:** Pr			^b^
		**d:** Ph			^b^
**8**	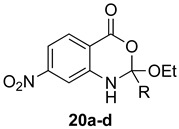	**a:** Me	1(2)	24(1.5)	84(87)
		**b:** Et	1(2)	24(1.5)	86(88)
		**c:** Pr	1(2)	24(1.5)	85(88)
		**d:** Ph	1	24	90
**9**	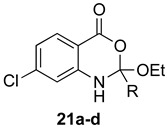	**a:** Me	1	24	84
		**b:** Et	1(2)	48(0.75)	84(86)
		**c:** Pr	1(2)	48(0.75)	83(84)
		**d:** Ph	1	48	85
**10**	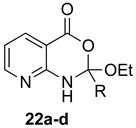	**a:** Me	1	24	85
		**b:** Et			^d^
		**c:** Pr			^d^
		**d:** Ph			^d^

^a^ For anthranilic acid (1 equivalent): Method 1: Orthoester (4.5 equivalents), AcOH (2.6 equivalents), neat, 100 °C, for the indicated time; Method 2: Orthoester (2.0–2.7 equivalents), neat, MW, 100 °C, for the indicated time; b Only the 4*H*-benzo*[d]*[1,3]oxazin-4-one product was formed; c An inseparable mixture of the 4*H*-benzo*[d]*[1,3]oxazin-4-one and the dihydro product was formed; d Only ethyl 2-aminonicotinate was formed from esterification of the substrate acid.

## Supplementary Materials

The supplemental materials are available online.
