# Supplementary material for: 4H-Benzo[d][1,3]oxazin-4-ones and Dihydro Analogs from Substituted Anthranilic Acids and Orthoesters
Source: Molecules. 2019 Oct 1;24(19):3555. doi: 10.3390/molecules24193555 (PMC6804250; doi:10.3390/molecules24193555)

4*H*-Benzo[*d*][1,3]oxazin-4-ones and Dihydro Analogs from Substituted Anthranilic Acids and Orthoesters

Joel K. Annor-Gyamfi and Richard A. Bunce *

Department of Chemistry Oklahoma State University, Stillwater, OK 74078-3071 USA; jannorg@okstate.edu (J.K.A-G.)

* Correspondence: rab@okstate.edu; Tel.: +1-405-744-5952

Supplemental Information

Spectra for Products **page**

^{1}H and ^{13}C NMR for *2-Methyl-4H-benzo[d][1,3]oxazin-4-one* (**11a**).. 3
^{1}H and ^{13}C NMR for *2-Ethyl-4H-benzo[d][1,3]oxazin-4-one* (**11b**).. 4
^{1}H and ^{13}C NMR for *2-Phenyl-4H-benzo[d][1,3]oxazin-4-one* (**11d**).. 5
^{1}H and ^{13}C NMR for *2,6-Dimethyl-4H-benzo[d][1,3]oxazin-4-one* (**12a**).................................. 6
^{1}H and ^{13}C NMR for *2-Ethyl-6-methyl-4H-benzo[d][1,3]oxazin-4-one* (**12b**)............................ 7
^{1}H and ^{13}C NMR for *6-Methyl-2-phenyl-4H-benzo[d][1,3]oxazin-4-one* (**12d**).......................... 8
^{1}H and ^{13}C NMR for *7-Methoxy-2-methyl-4H-benzo[d][1,3]oxazin-4-one* (**13a**)........................ 9
^{1}H and ^{13}C NMR for *2-Ethyl-7-methoxy-4H-benzo[d][1,3]oxazin-4-one* (**13b**)......................... 10
^{1}H and ^{13}C NMR for *7-Methoxy-2-propyl-4H-benzo[d][1,3]oxazin-4-one* (**13c**)....................... 11
^{1}H and ^{13}C NMR for *7-Methoxy-2-phenyl-4H-benzo[d][1,3]oxazin-4-one* (**13d**)...................... 12
^{1}H and ^{13}C NMR for *7-Nitro-2-phenyl-4H-benzo[d][1,3]oxazin-4-one* (**14d**).......................... 13
^{1}H and ^{13}C NMR for *7-Chloro-2-methyl-4H-benzo[d][1,3]oxazin-4-one* (**15a**)......................... 14
^{1}H and ^{13}C NMR for *7-Chloro-2-phenyl-4H-benzo[d][1,3]oxazin-4-one* (**15d**)......................... 15
^{1}H and ^{13}C NMR for *(±)-2-Ethoxy-2-phenyl-1,2-dihydro-4H-benzo[d][1,3]oxazin-4-one* (**17d**)............ 16
^{1}H and ^{13}C NMR for *(±)-2-Ethoxy-2,6-dimethyl-1,2-dihydro-4H-benzo[d][1,3]oxazin-4-one* (**18a**)............ 17
^{1}H and ^{13}C NMR for *(±)-2-Ethoxy-2-ethyl-6-methyl-1,2-dihydro-4H-benzo[d][1,3]oxazin-4-one* (**18b**)............ 18
^{1}H and ^{13}C NMR for *(±)-2-Ethoxy-6-methyl-2-propyl-1,2-dihydro-4H-benzo[d][1,3]oxazin-4-one* (**18c**) 19
^{1}H and ^{13}C NMR for *(±)-2-Ethoxy-6-methyl-2-phenyl-1,2-dihydro-4H-benzo[d][1,3]oxazin-4-one* (**18d**) 20
^{1}H and ^{13}C NMR for *(±)-2-Ethoxy-2-methyl-7-nitro-1,2-dihydro-4H-benzo[d][1,3]oxazin-4-one* (**20a**) 21
^{1}H and ^{13}C NMR for *(±)-2-Ethoxy-2-ethyl-7-nitro-1,2-dihydro-4H-benzo[d][1,3]oxazin-4-one* (**20b**) 22
^{1}H and ^{13}C NMR for *(±)-2-Ethoxy-7-nitro-2-propyl-1,2-dihydro-4H-benzo[d][1,3]oxazin-4-one* (**20c**)............ 23
^{1}H and ^{13}C NMR for *(±)-2-Ethoxy-7-nitro-2-phenyl-1,2-dihydro-4H-benzo[d][1,3]oxazin-*

4-one (**20d**).. 24
^{1}H and ^{13}C NMR for *(±)-7-Chloro-2-ethoxy-2-methyl-1,2-dihydro-4H-benzo[d][1,3]oxazin-4-one* (**21a**).. 25
^{1}H and ^{13}C NMR for *(±)-7-Chloro-2-ethoxy-2-ethyl-1,2-dihydro-4H-benzo[d][1,3]oxazin-4-one* (**21b**) 26
^{1}H and ^{13}C NMR for *(±)-7-Chloro-2-ethoxy-2-propyl-1,2-dihydro-4H-benzo[d][1,3]oxazin-4-one* (**21c**) 27
^{1}H and ^{13}C NMR for *(±)-7-Chloro-2-ethoxy-2-phenyl-1,2-dihydro-4H-benzo[d][1,3]oxazin-4-one* (**21d**) 28
^{1}H and ^{13}C NMR for *(±)-2-Ethoxy-2-methyl-1,2-dihydro-4H-pyrido[2,3-d][1,3]oxazin-4-one* (**22a**) 29

[1]H for *2-Methyl-4H-benzo[d][1,3]oxazin-4-one* (**11a**)

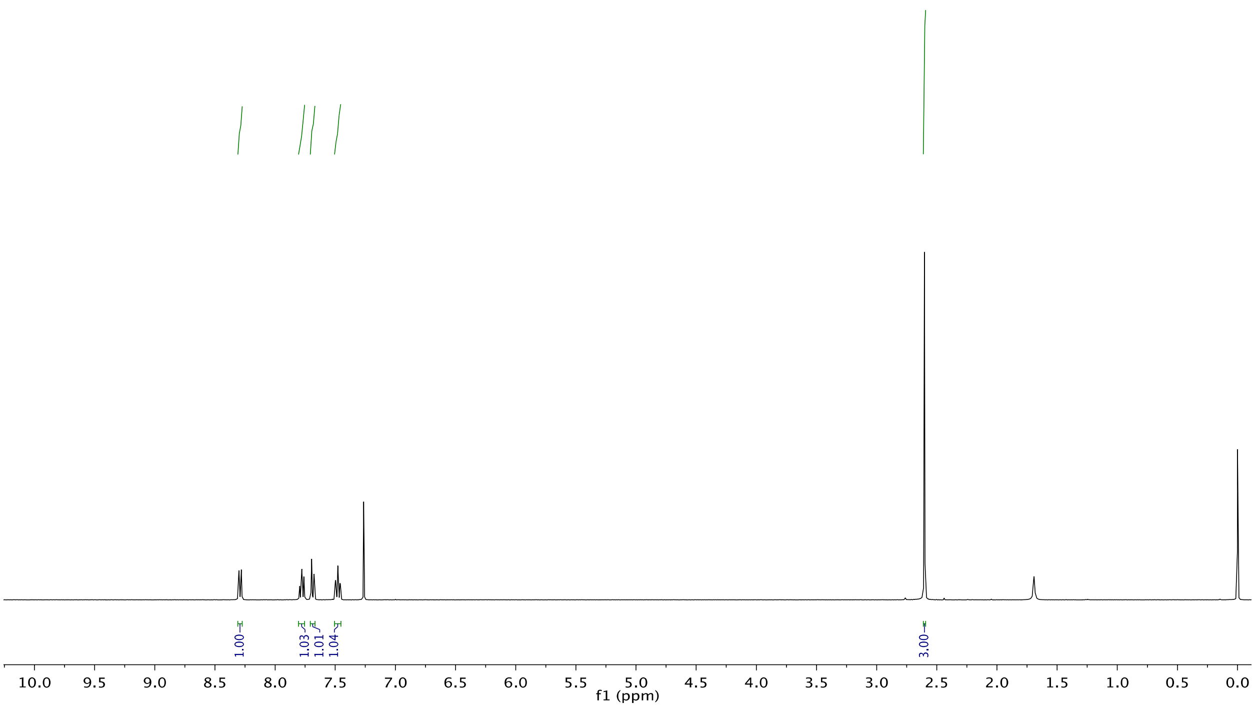

[13]C for *2-Methyl-4H-benzo[d][1,3]oxazin-4-one* (**11a**)

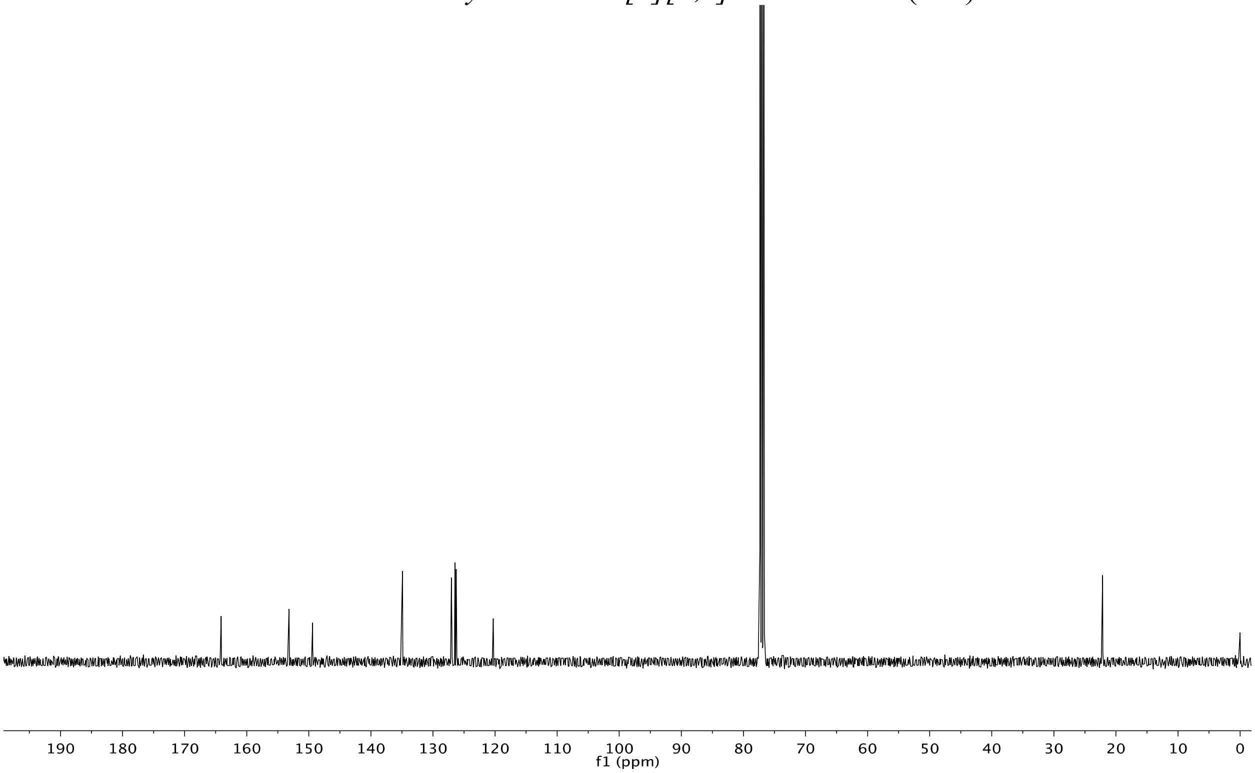

^{1}H for *2-Ethyl-4H-benzo[d][1,3]oxazin-4-one* (**11b**)

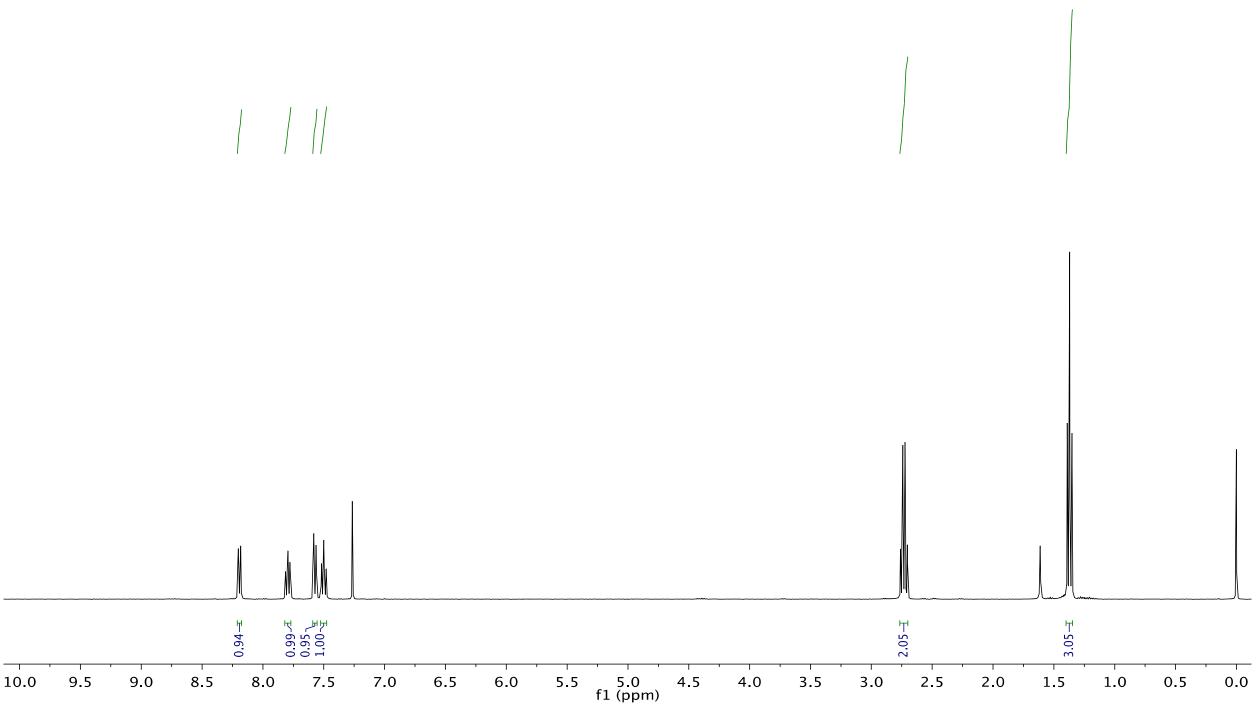

^{13}C for *2-Ethyl-4H-benzo[d][1,3]oxazin-4-one* (**11b**)

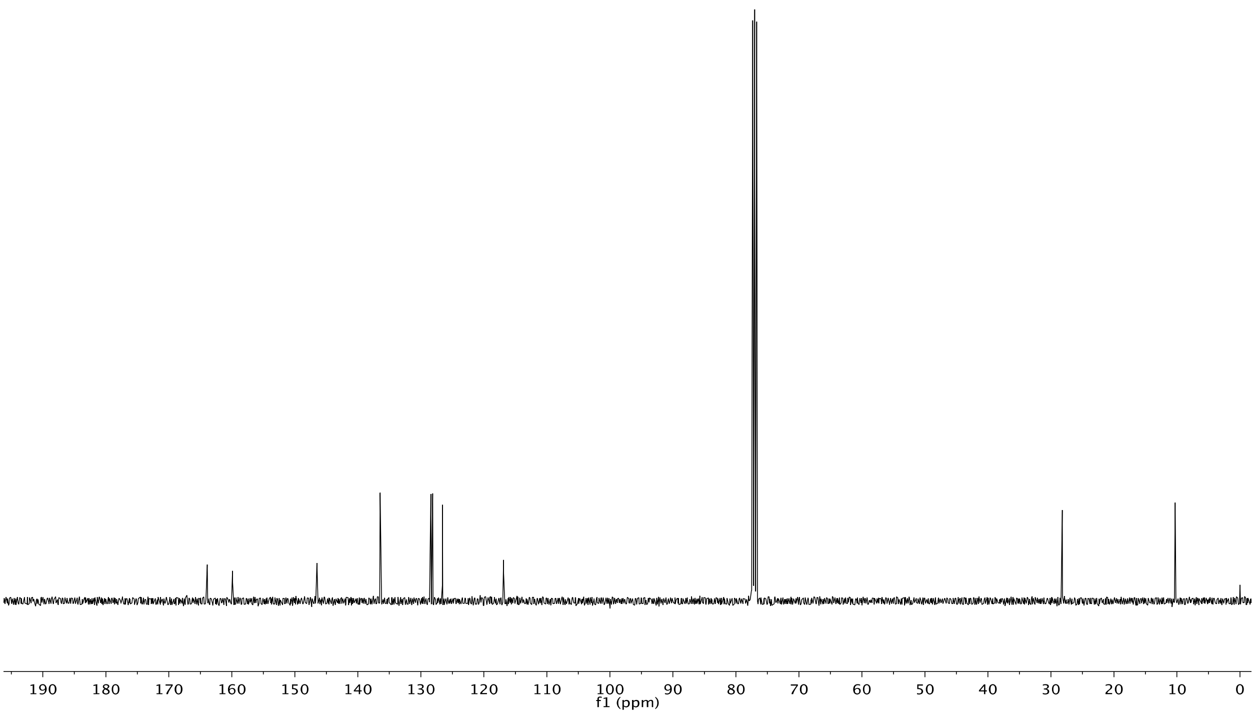

[1]H for *2-Phenyl-4H-benzo[d][1,3]oxazin-4-one* (**11d**)

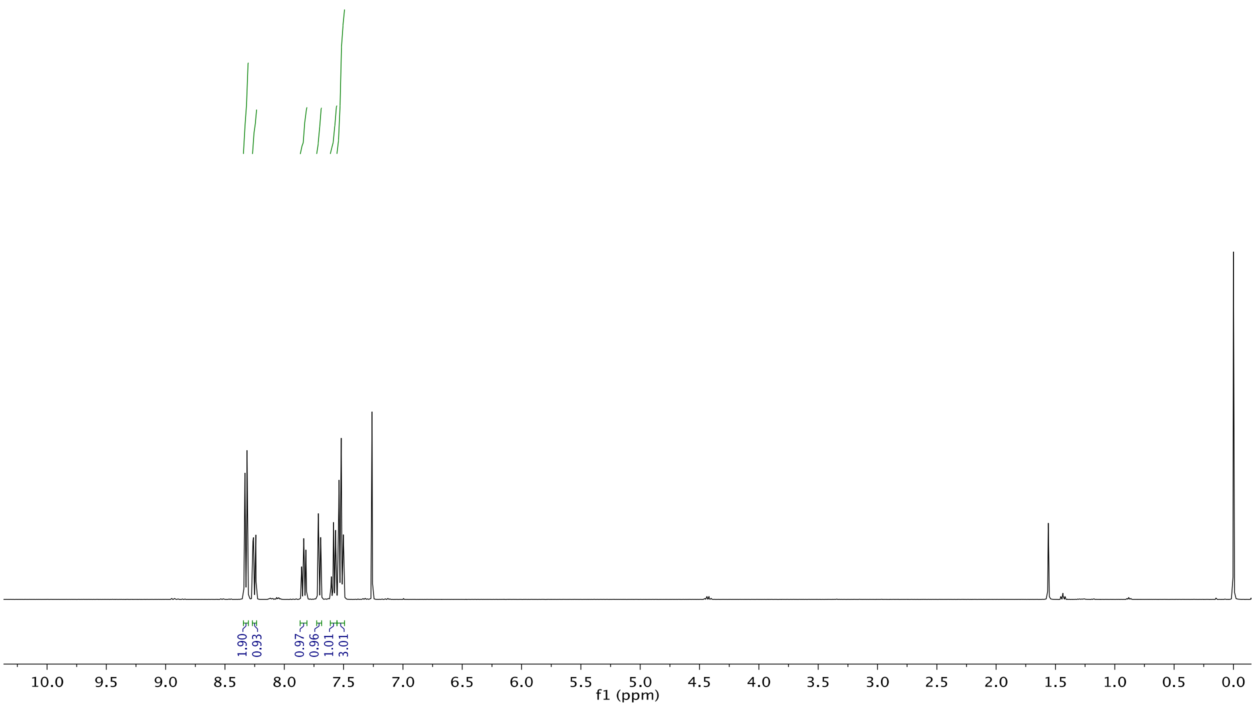

[13]C for *2-Phenyl-4H-benzo[d][1,3]oxazin-4-one* (**11d**)

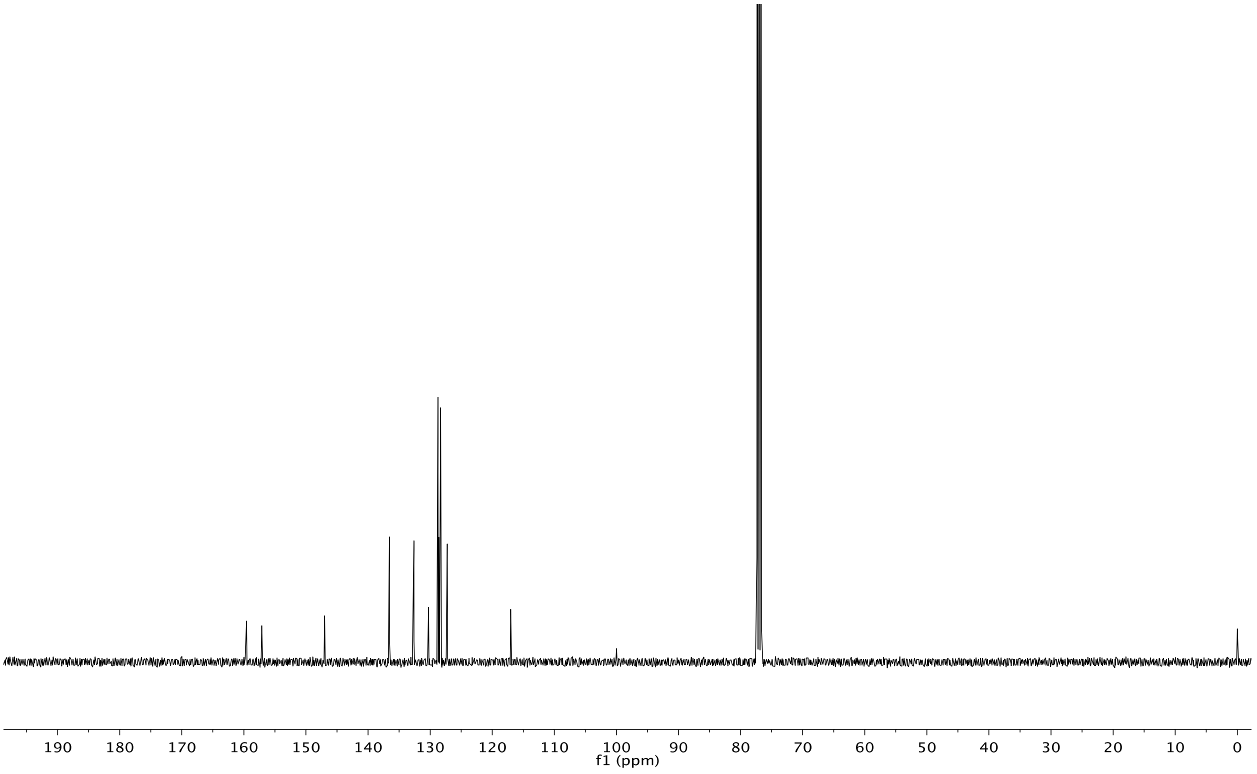

[1]H for *2,6-Dimethyl-4H-benzo[d][1,3]oxazin-4-one* (**12a**)

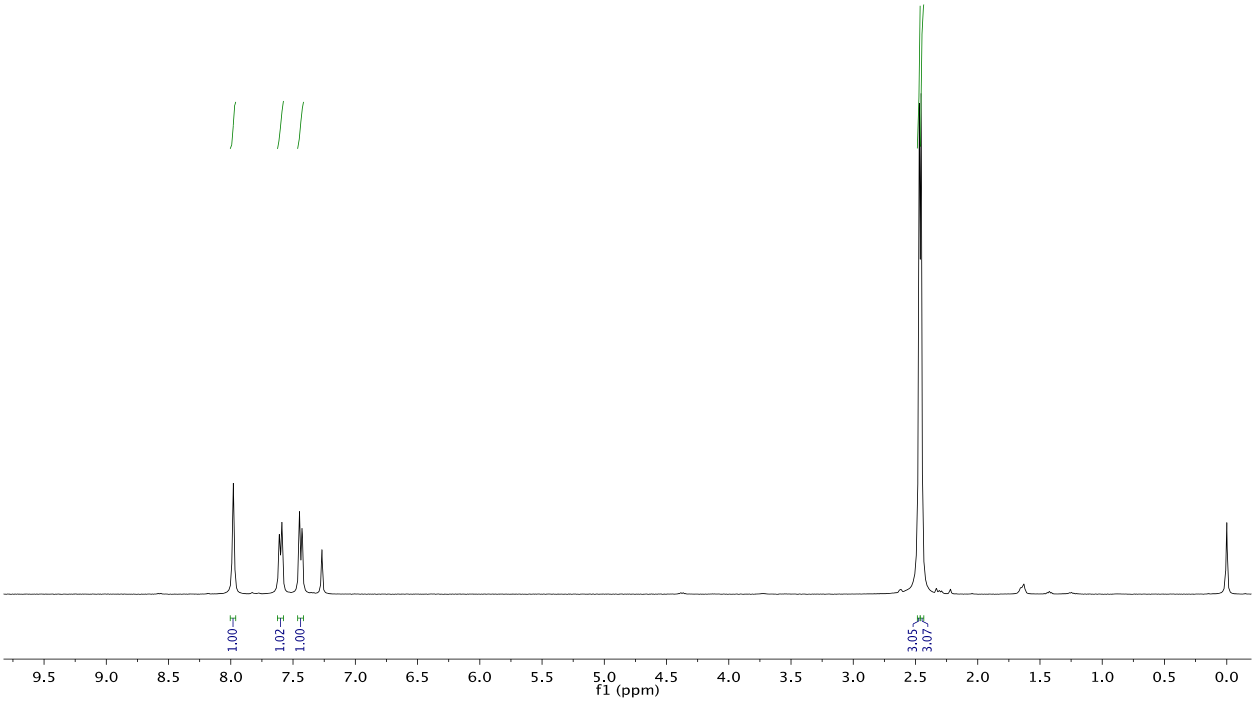

[13]C for *2,6-Dimethyl-4H-benzo[d][1,3]oxazin-4-one* (**12a**)

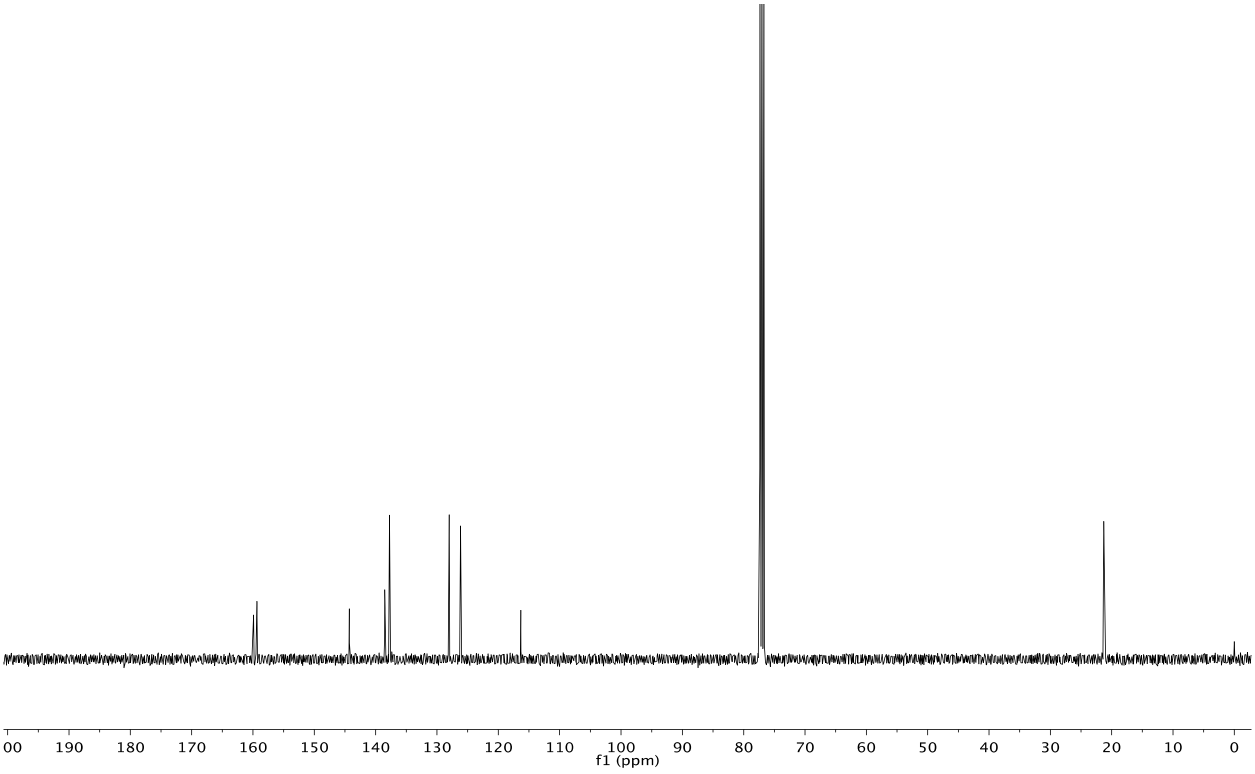

^{1}H for *2-Ethyl-6-methyl-4H-benzo[d][1,3]oxazin-4-one* (**12b**)

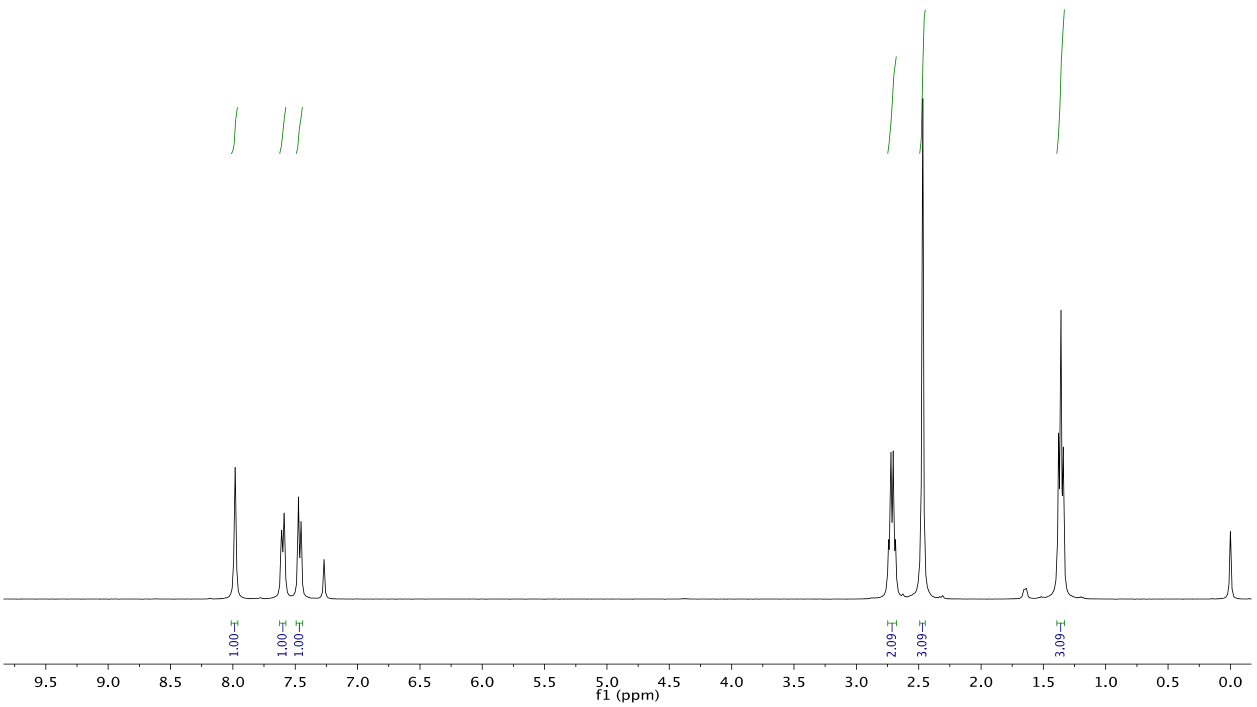

^{13}C for *2-Ethyl-6-methyl-4H-benzo[d][1,3]oxazin-4-one* (**12b**)

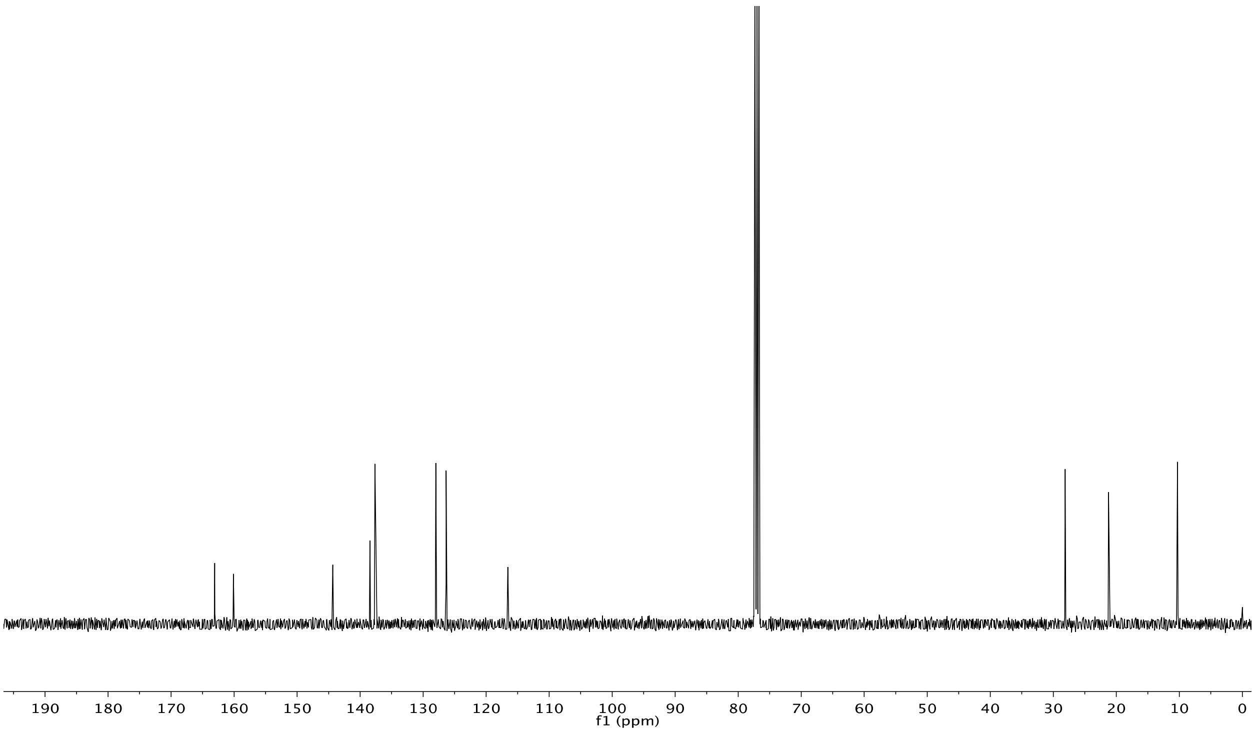

[1]H for *6-Methyl-2-phenyl-4H-benzo[d][1,3]oxazin-4-one* (**12d**)

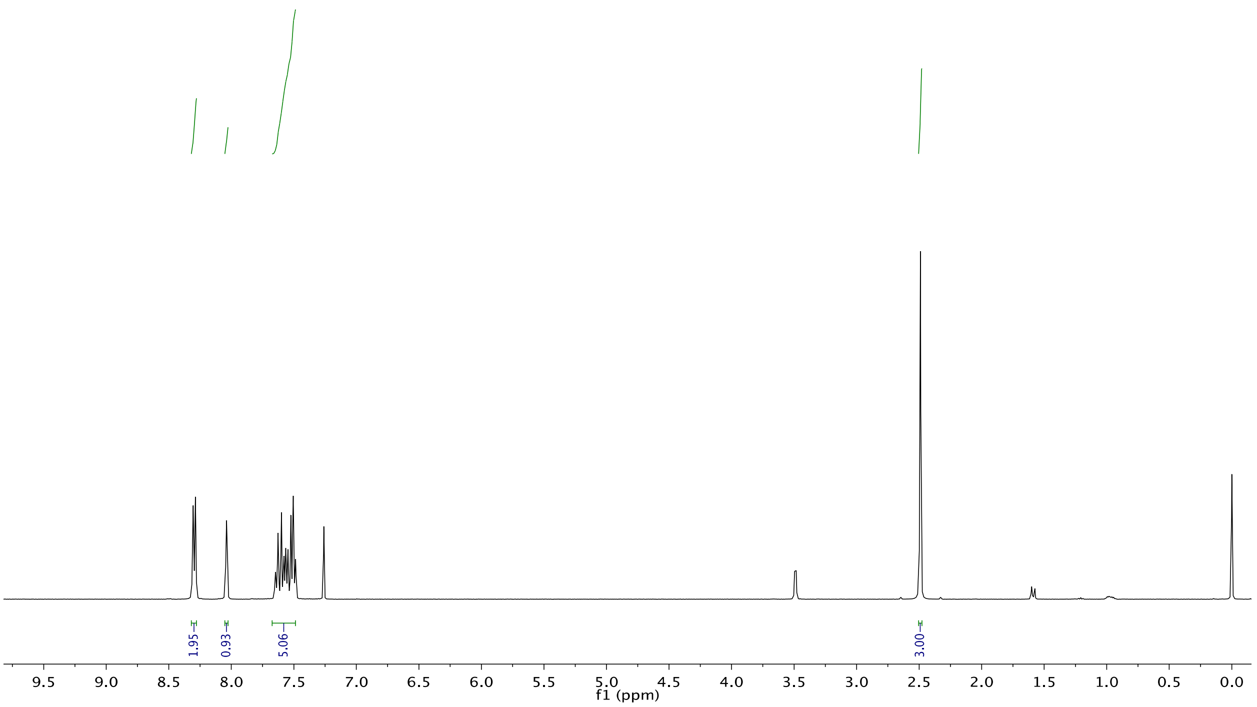

[13]C for *6-Methyl-2-phenyl-4H-benzo[d][1,3]oxazin-4-one* (**12d**)

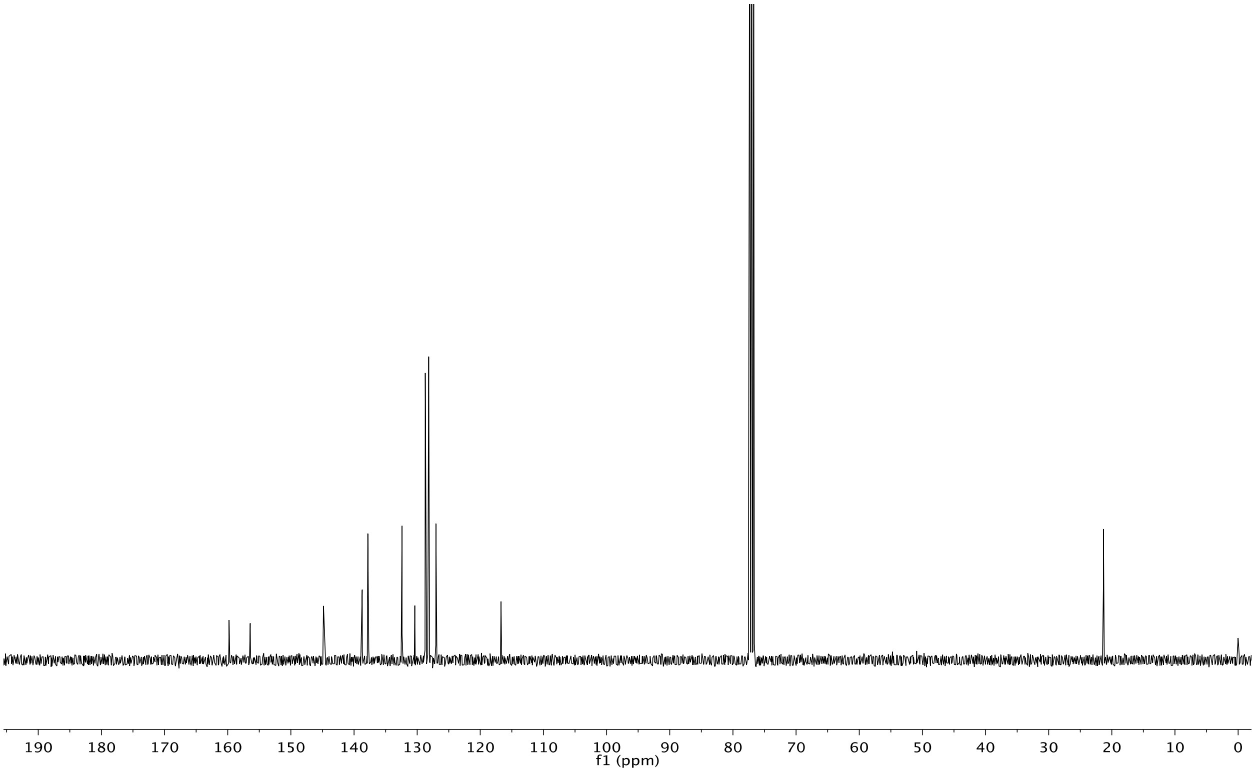

^{1}H for *7-Methoxy-2-methyl-4H-benzo[d][1,3]oxazin-4-one* (**13a**)

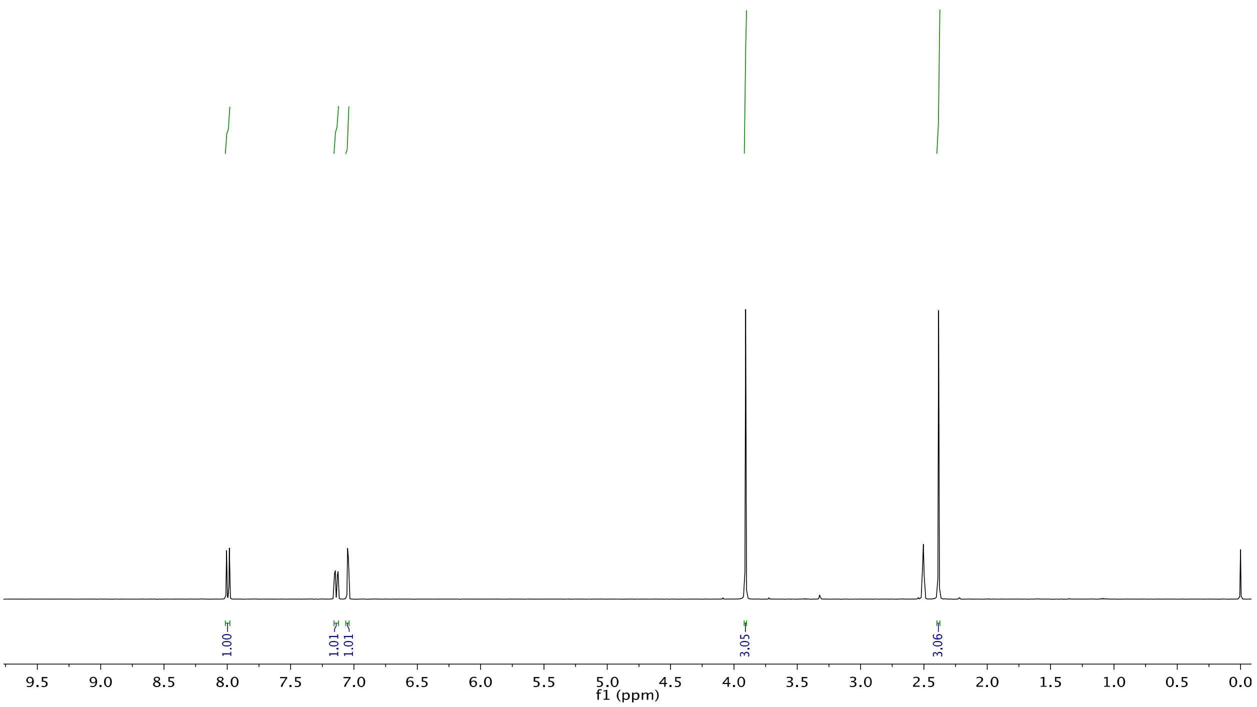

^{13}C for *7-Methoxy-2-methyl-4H-benzo[d][1,3]oxazin-4-one* (**13a**)

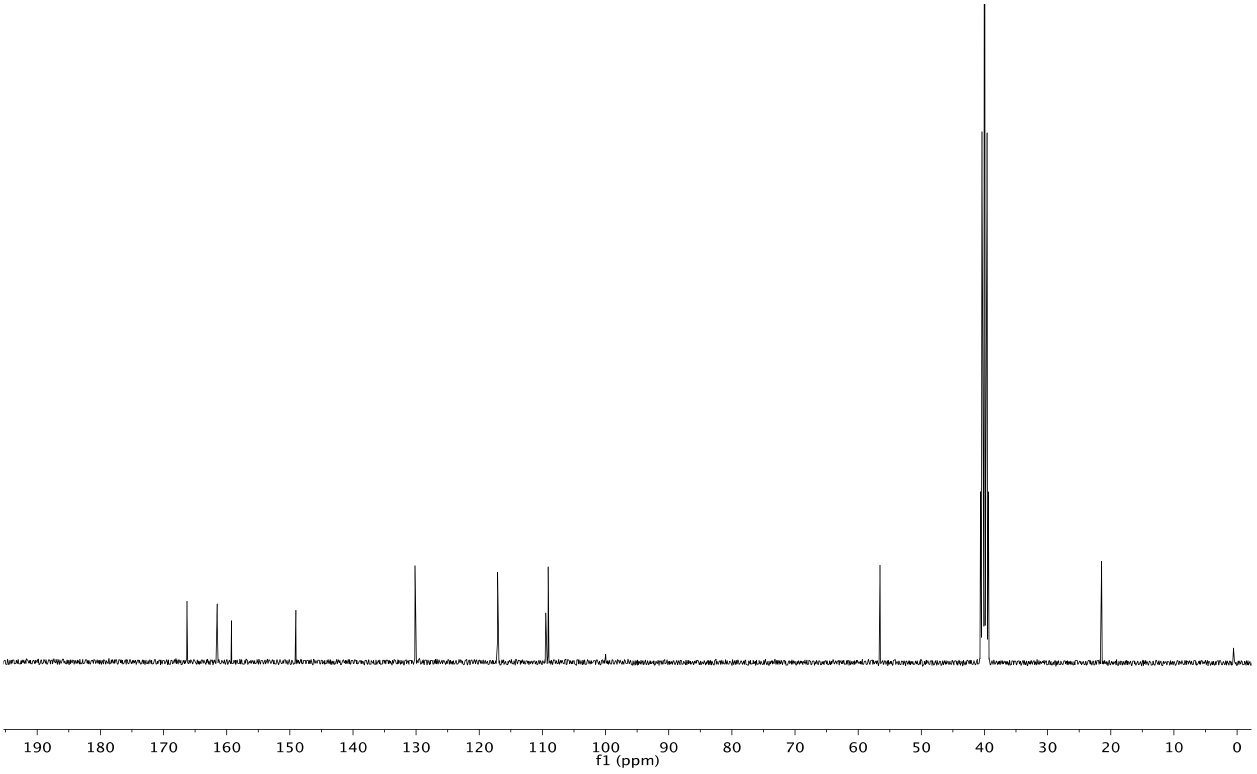

[1]H for *2-Ethyl-7-methoxy-4H-benzo[d][1,3]oxazin-4-one* (**13b**)

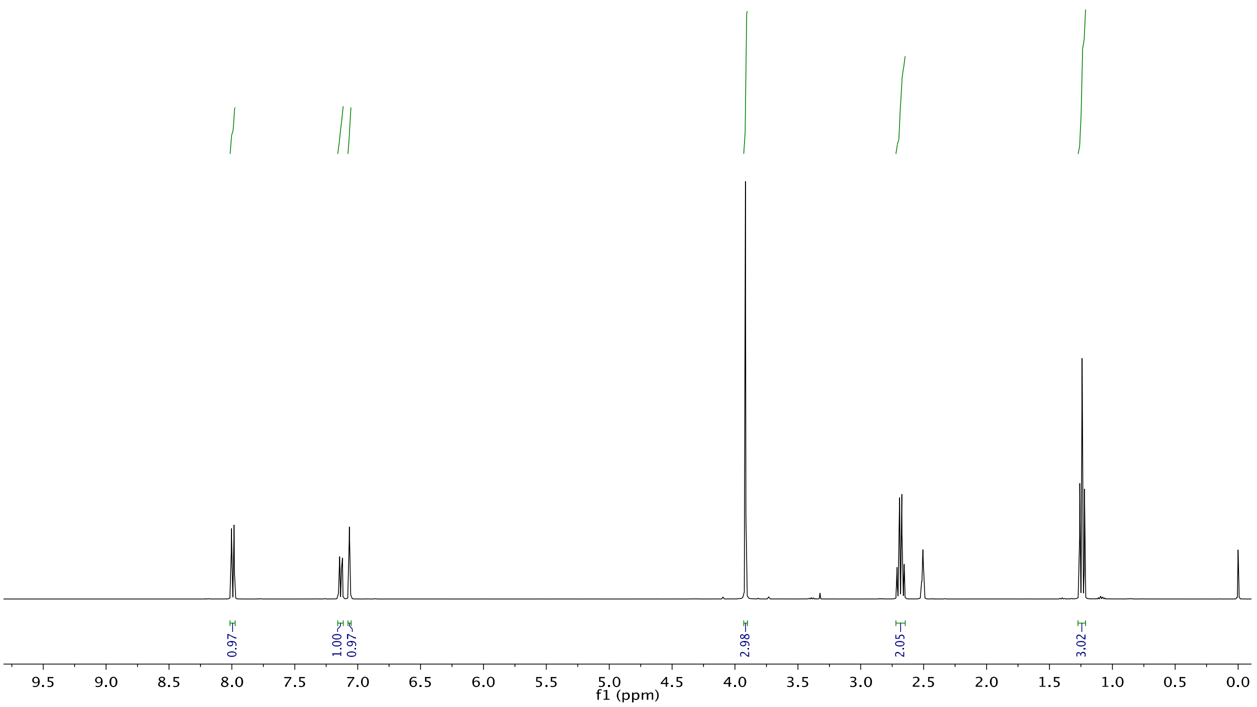

[13]C for *2-Ethyl-7-methoxy-4H-benzo[d][1,3]oxazin-4-one* (**13b**)

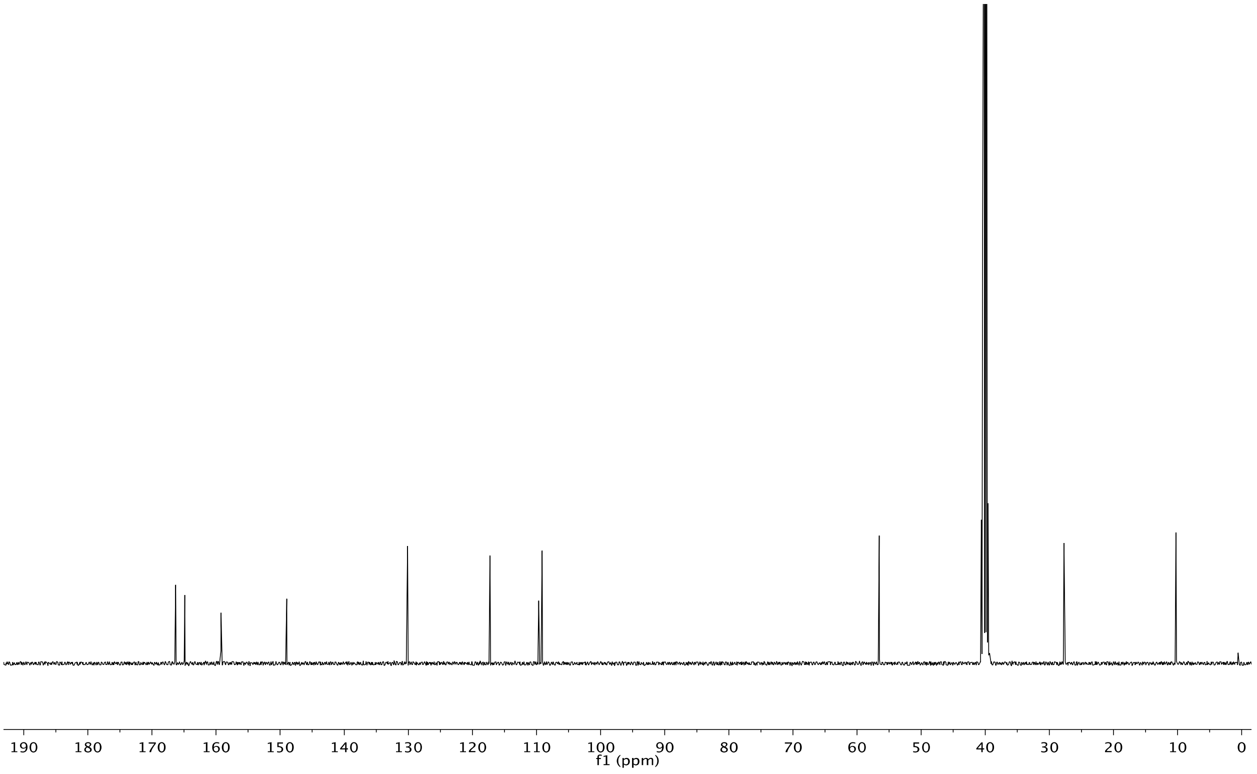

[1]H for *7-Methoxy-2-propyl-4H-benzo[d][1,3]oxazin-4-one* (**13c**)

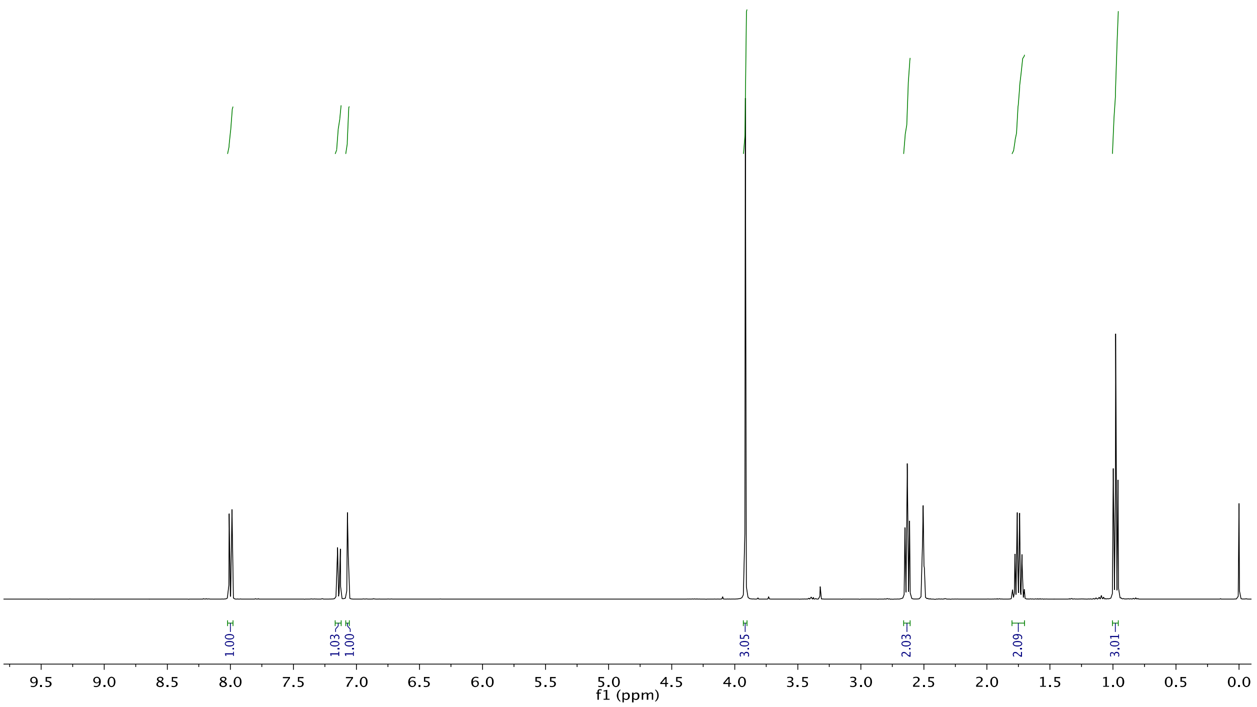

[13]C for *7-Methoxy-2-propyl-4H-benzo[d][1,3]oxazin-4-one* (**13c**)

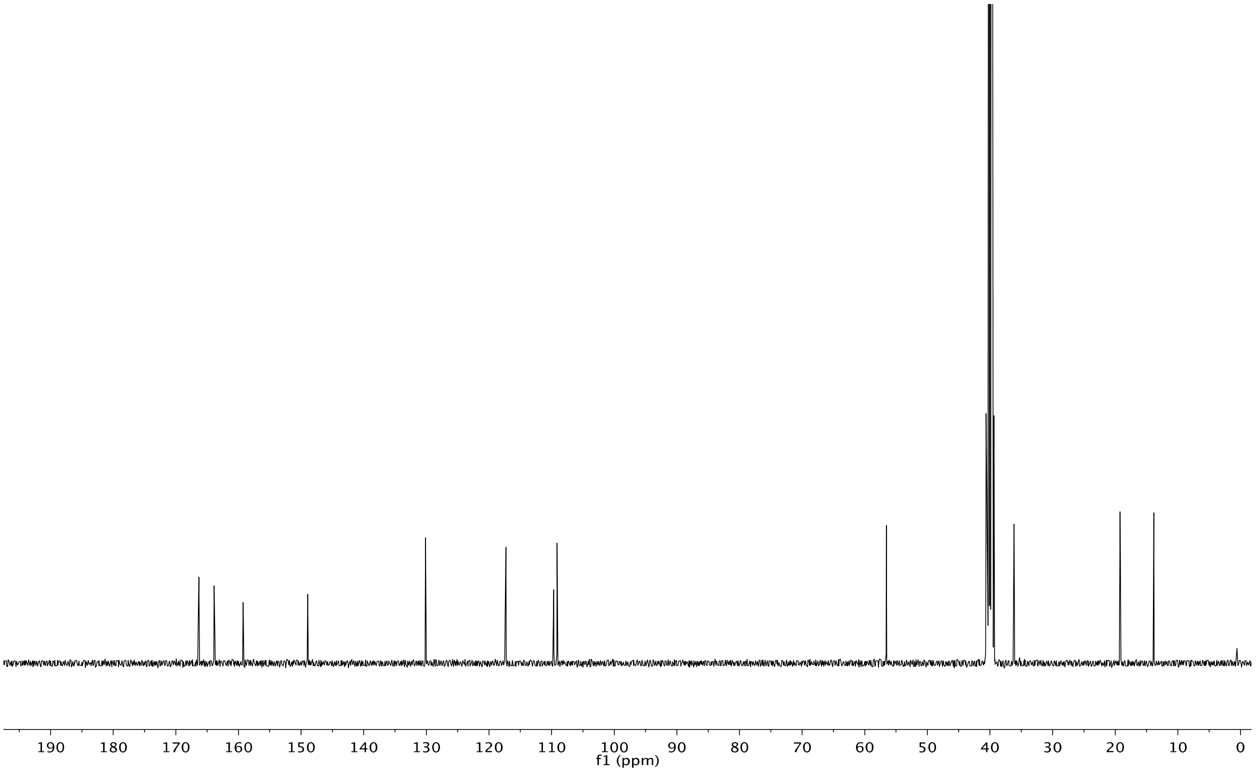

[1]H for *7-Methoxy-2-phenyl-4H-benzo[d][1,3]oxazin-4-one* (**13d**)

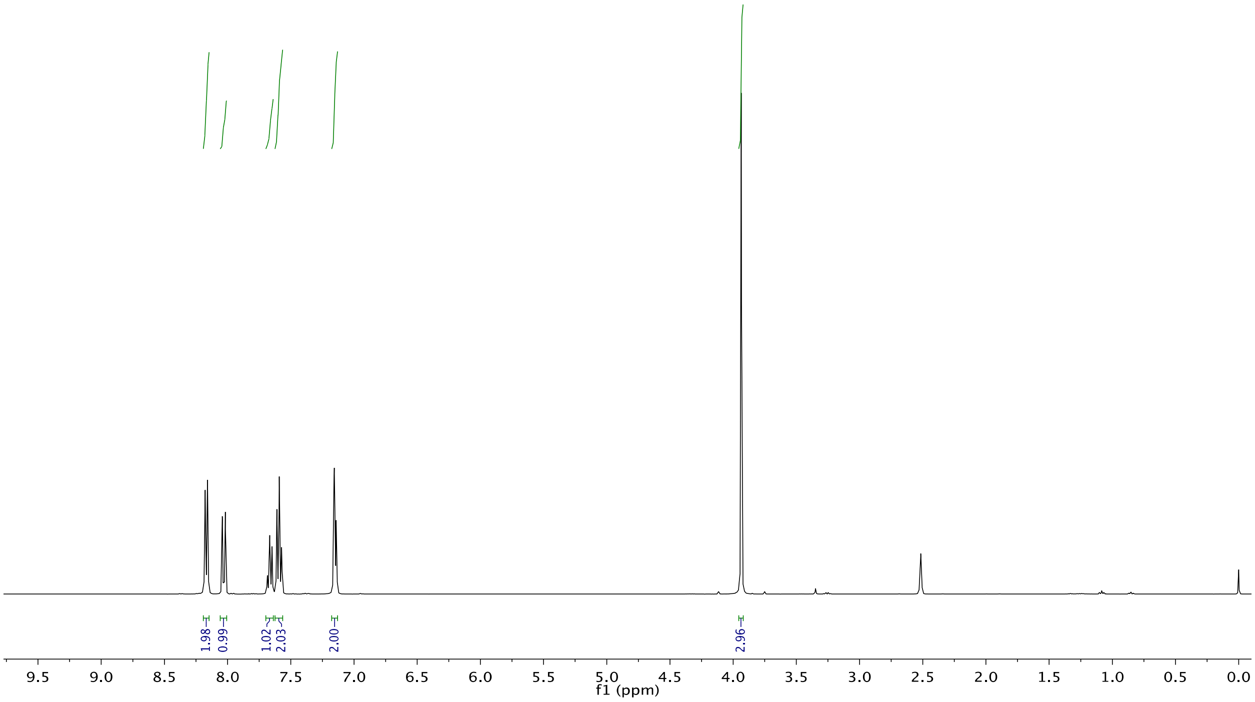

[13]C for *7-Methoxy-2-phenyl-4H-benzo[d][1,3]oxazin-4-one* (**13d**)

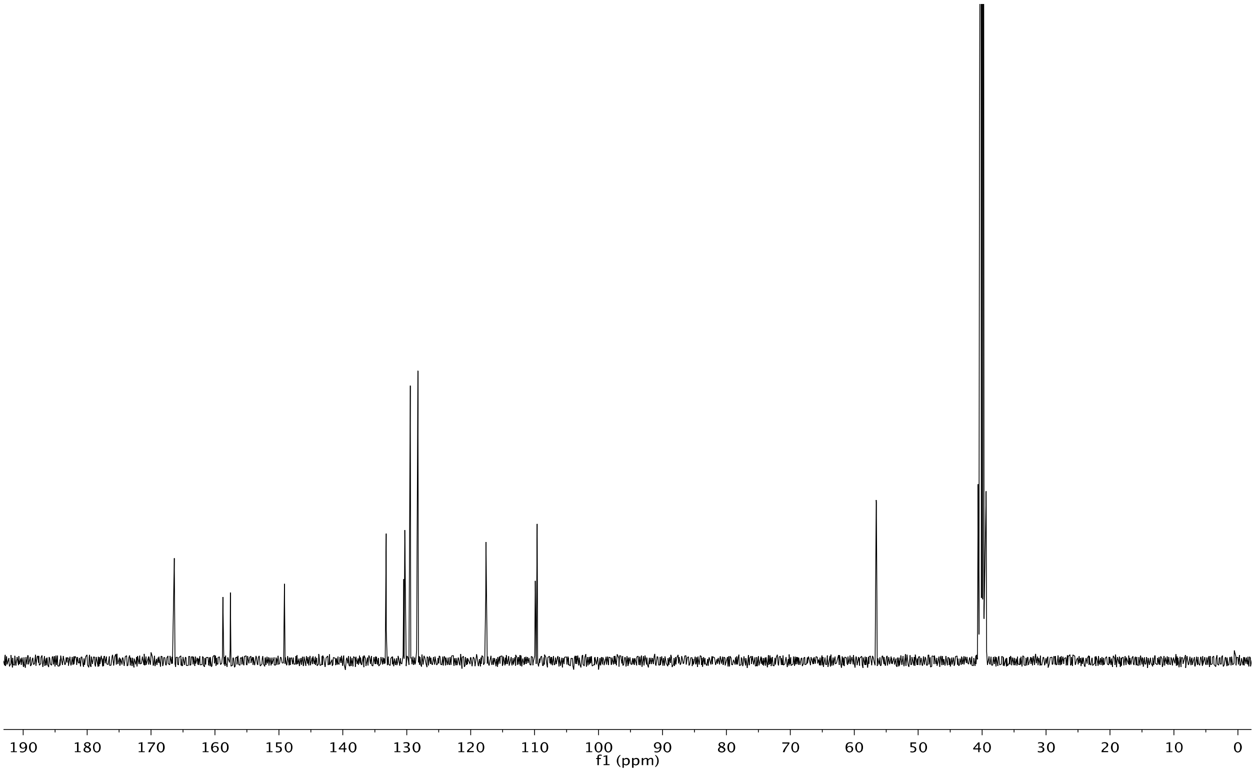

^{1}H for *7-Nitro-2-phenyl-4H-benzo[d][1,3]oxazin-4-one* (**14d**)

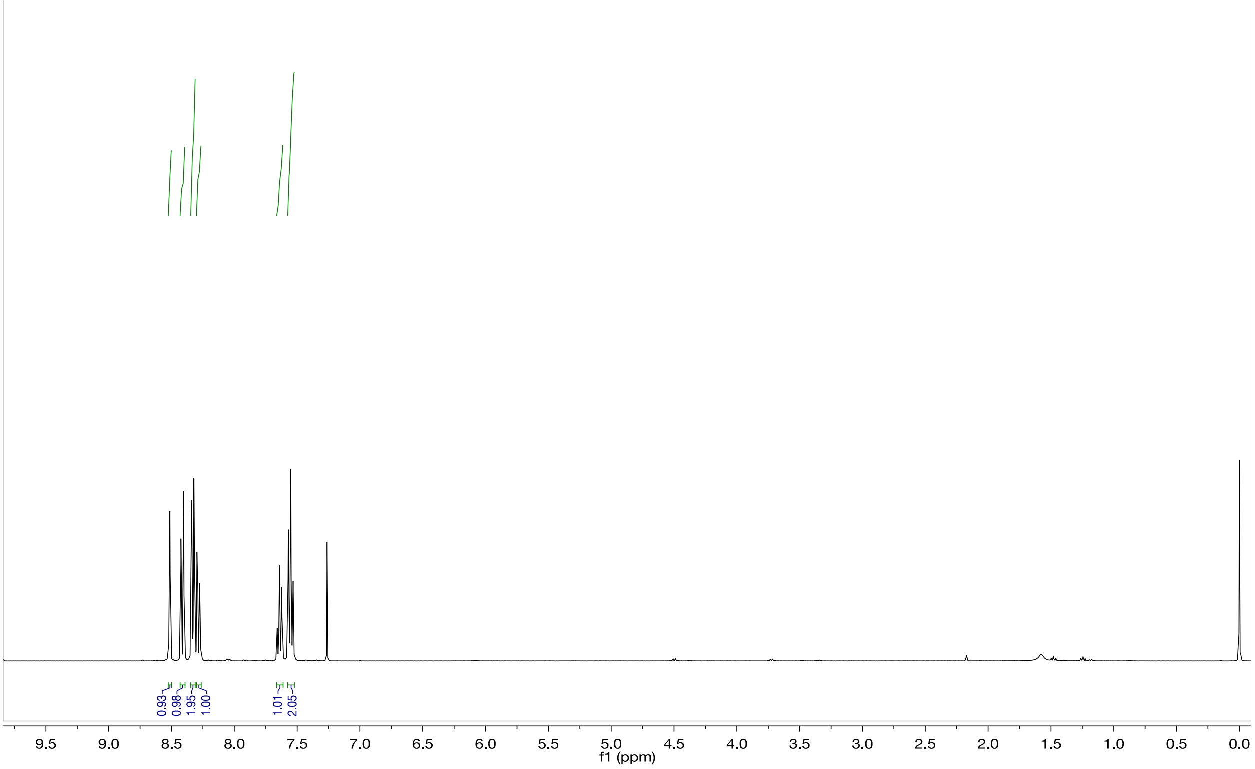

^{13}C for *7-Nitro-2-phenyl-4H-benzo[d][1,3]oxazin-4-one* (**14d**)

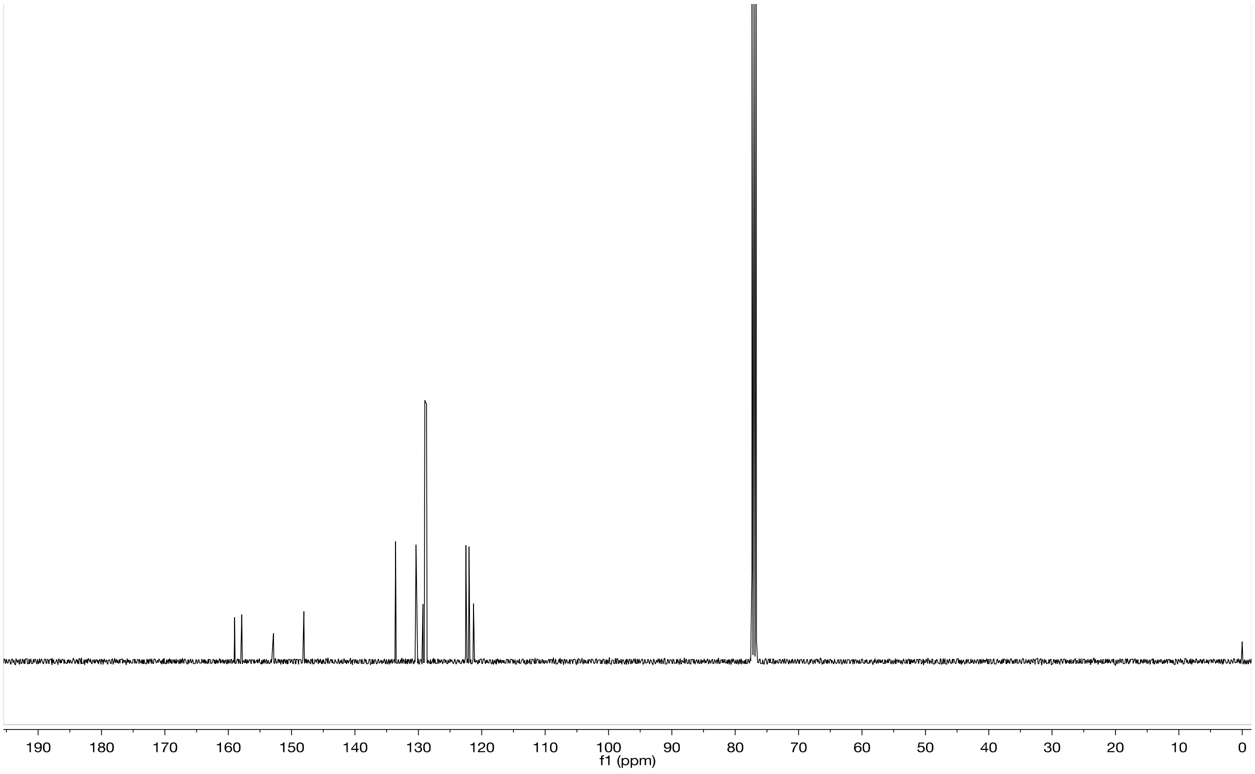

[1]H for *7-Chloro-2-methyl-4H-benzo[d][1,3]oxazin-4-one* (**15a**)

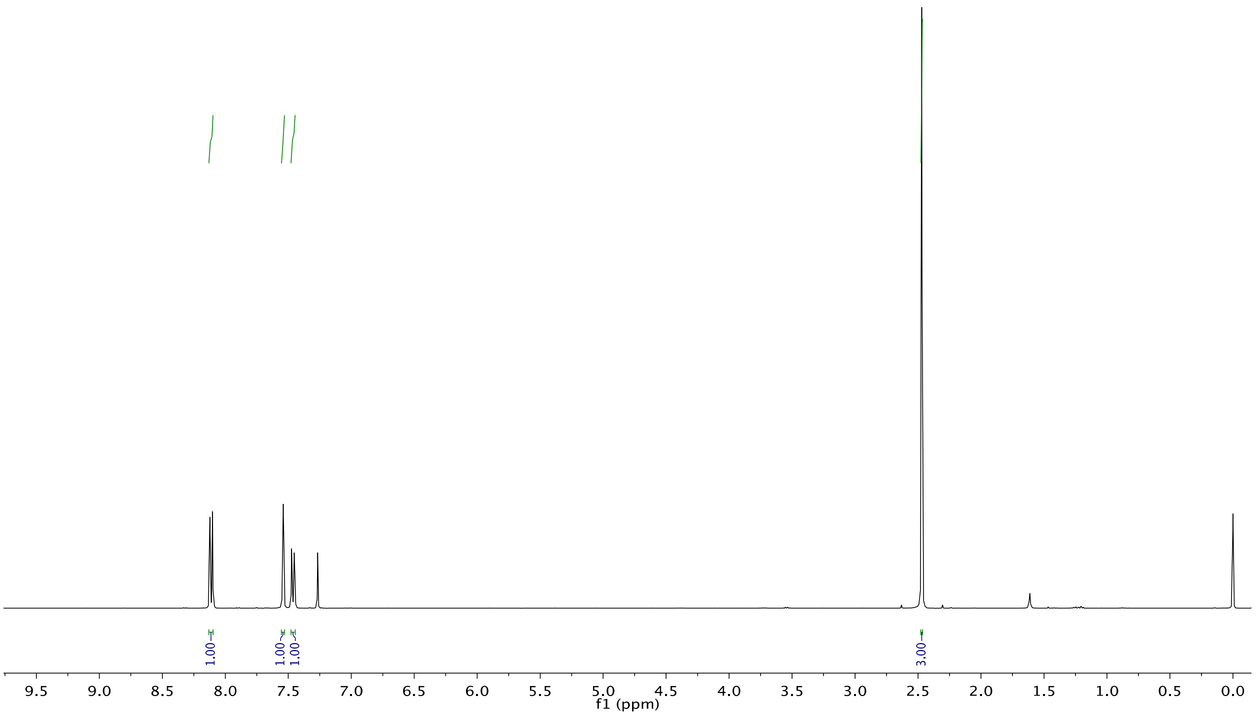

[13]C for *7-Chloro-2-methyl-4H-benzo[d][1,3]oxazin-4-one* (**15a**)

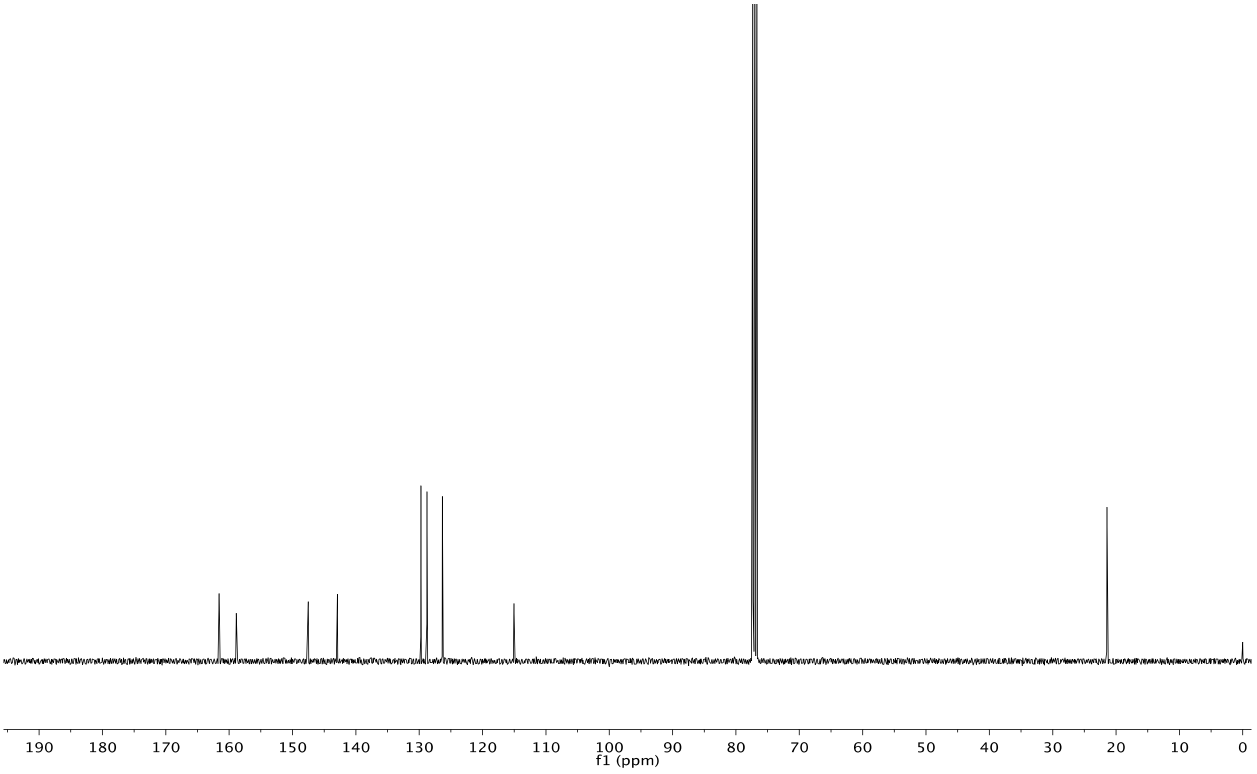

^{1}H for *7-Chloro-2-phenyl-4H-benzo[d][1,3]oxazin-4-one* (**15d**)

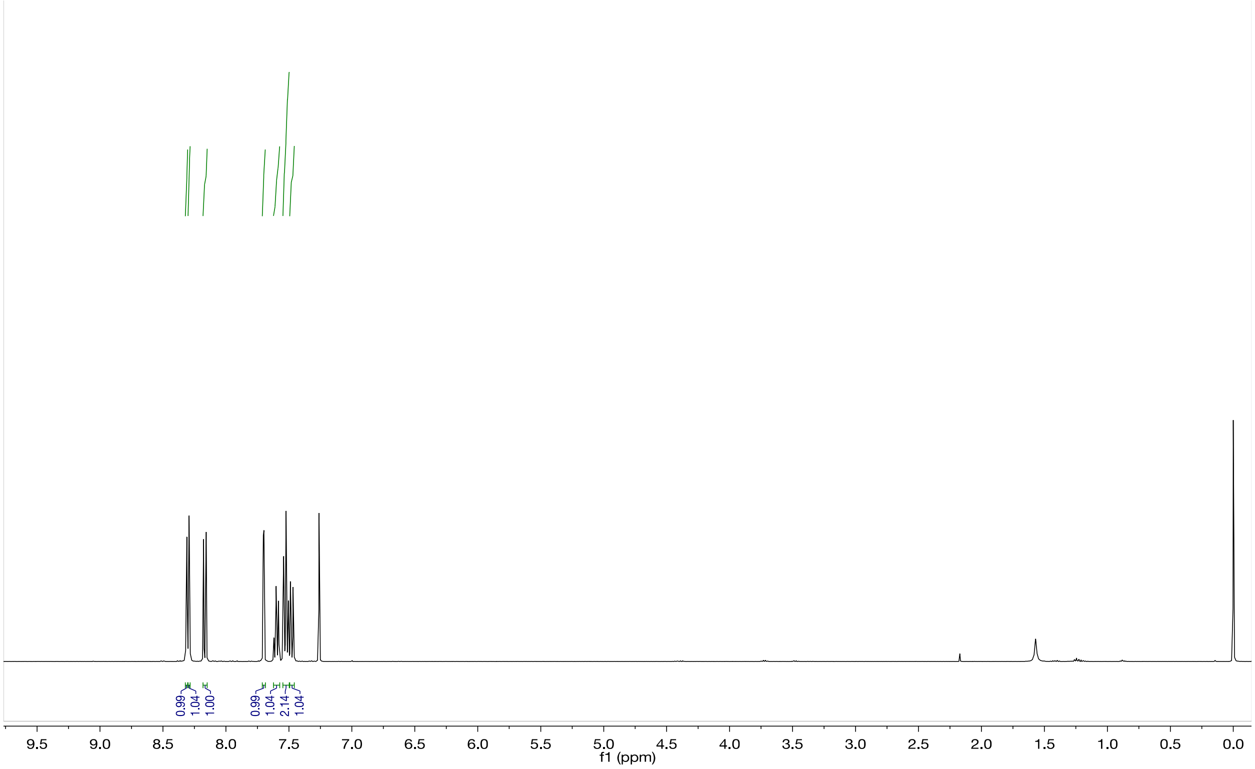

^{13}C for *7-Chloro-2-phenyl-4H-benzo[d][1,3]oxazin-4-one* (**15d**)

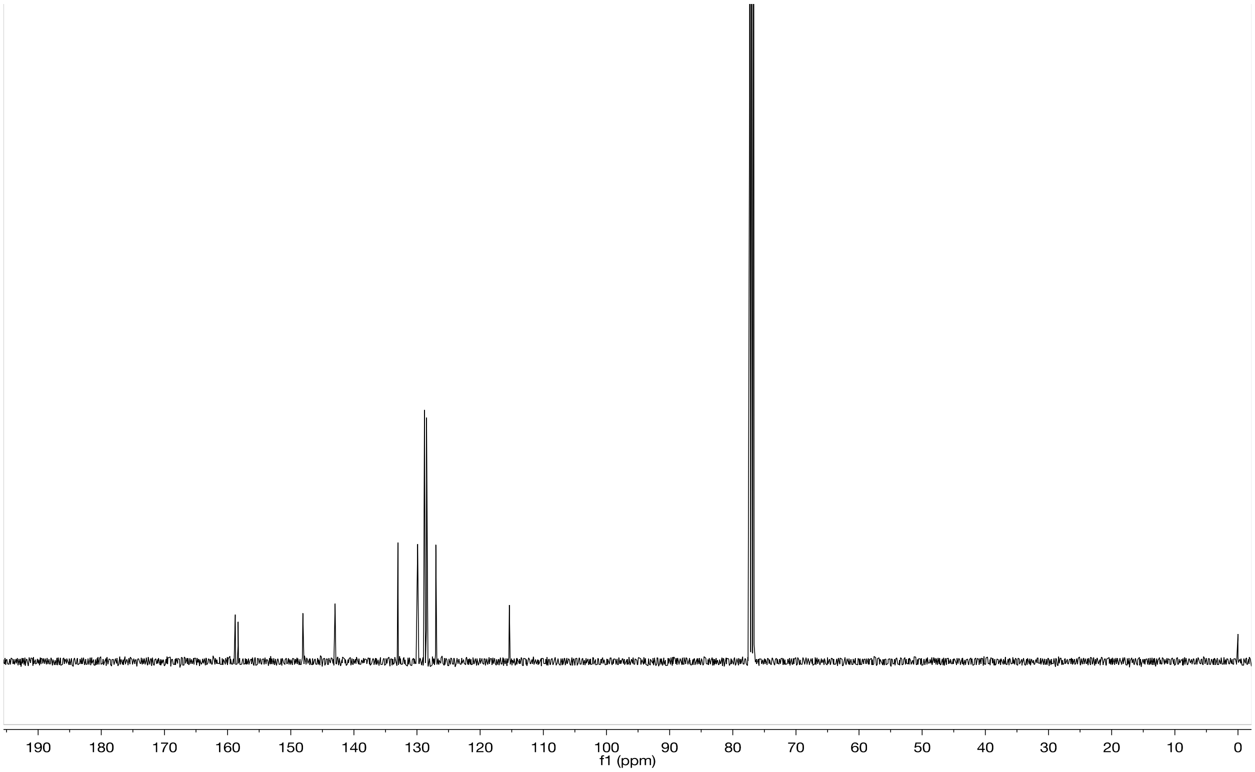

^{1}H for *(±)-2-Ethoxy-2-phenyl-1,2-dihydro-4H-benzo[d][1,3]oxazin-4-one* **(17d)**

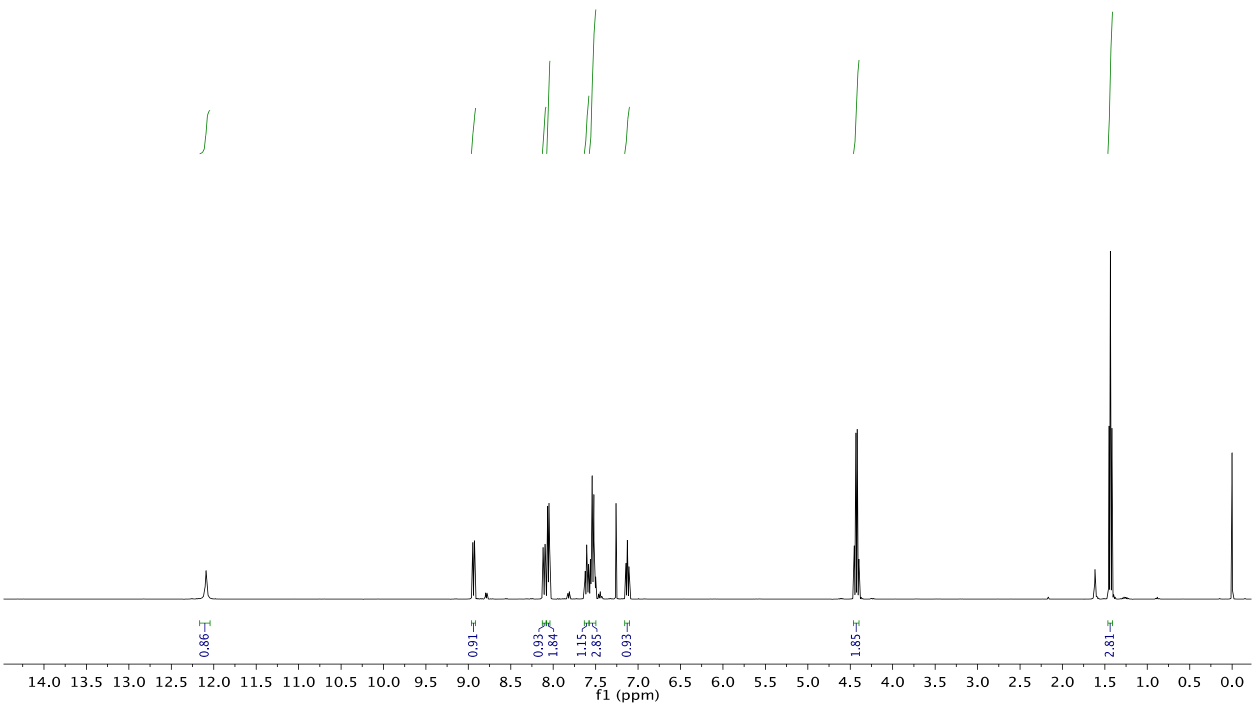

^{13}C for *(±)-2-Ethoxy-2-phenyl-1,2-dihydro-4H-benzo[d][1,3]oxazin-4-one* (**17d**)

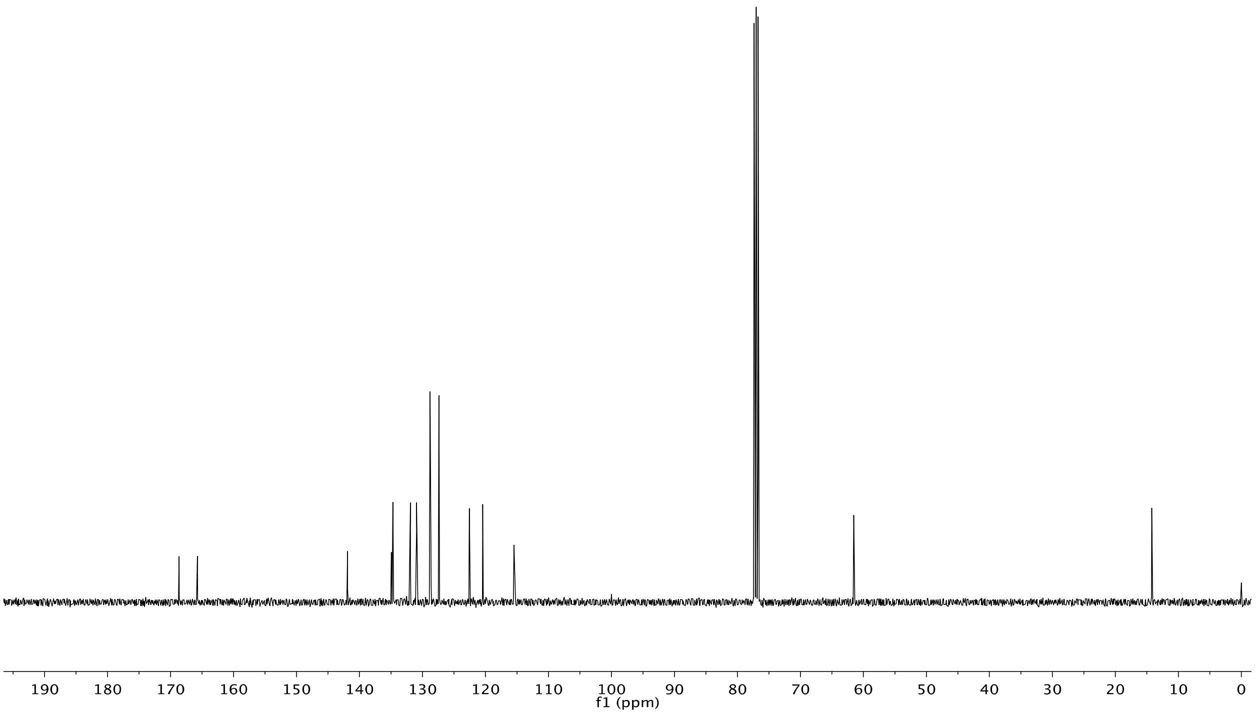

^{1}H for *(±)-2-Ethoxy-2,6-dimethyl-1,2-dihydro-4H-benzo[d][1,3]oxazin-4-one* (**18a**)

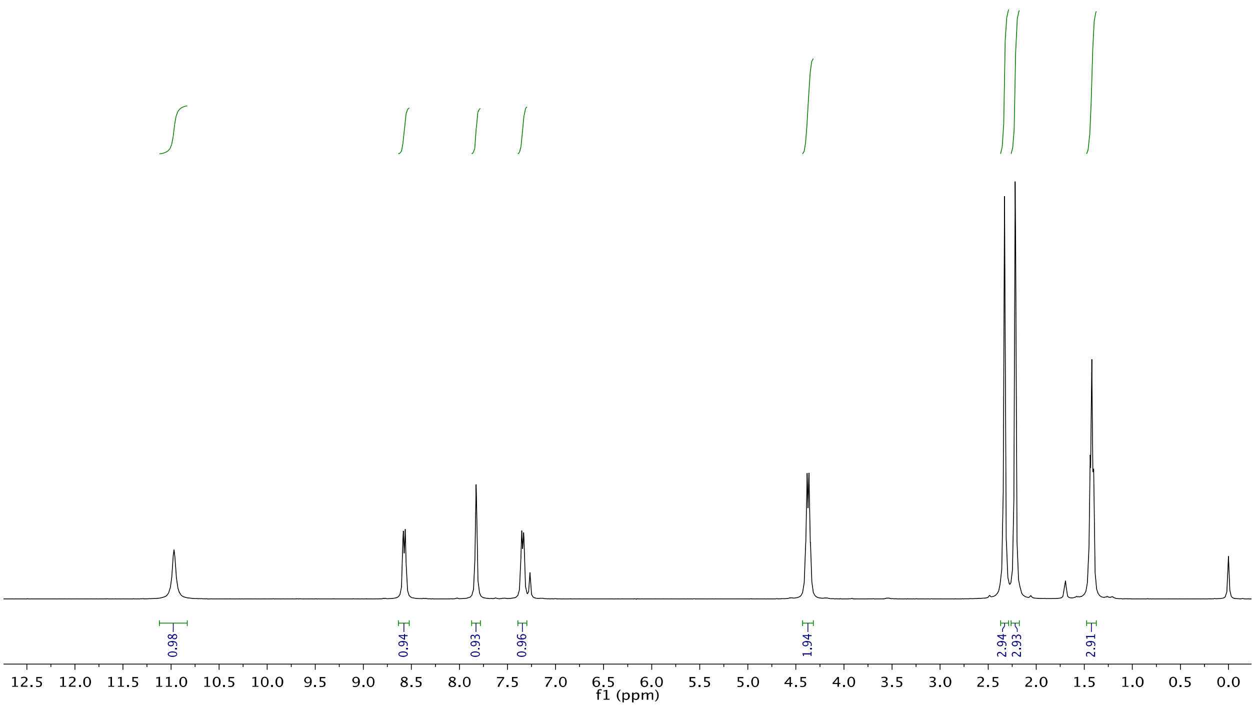

^{13}C for *(±)-2-Ethoxy-2,6-dimethyl-1,2-dihydro-4H-benzo[d][1,3]oxazin-4-one* (**18a**)

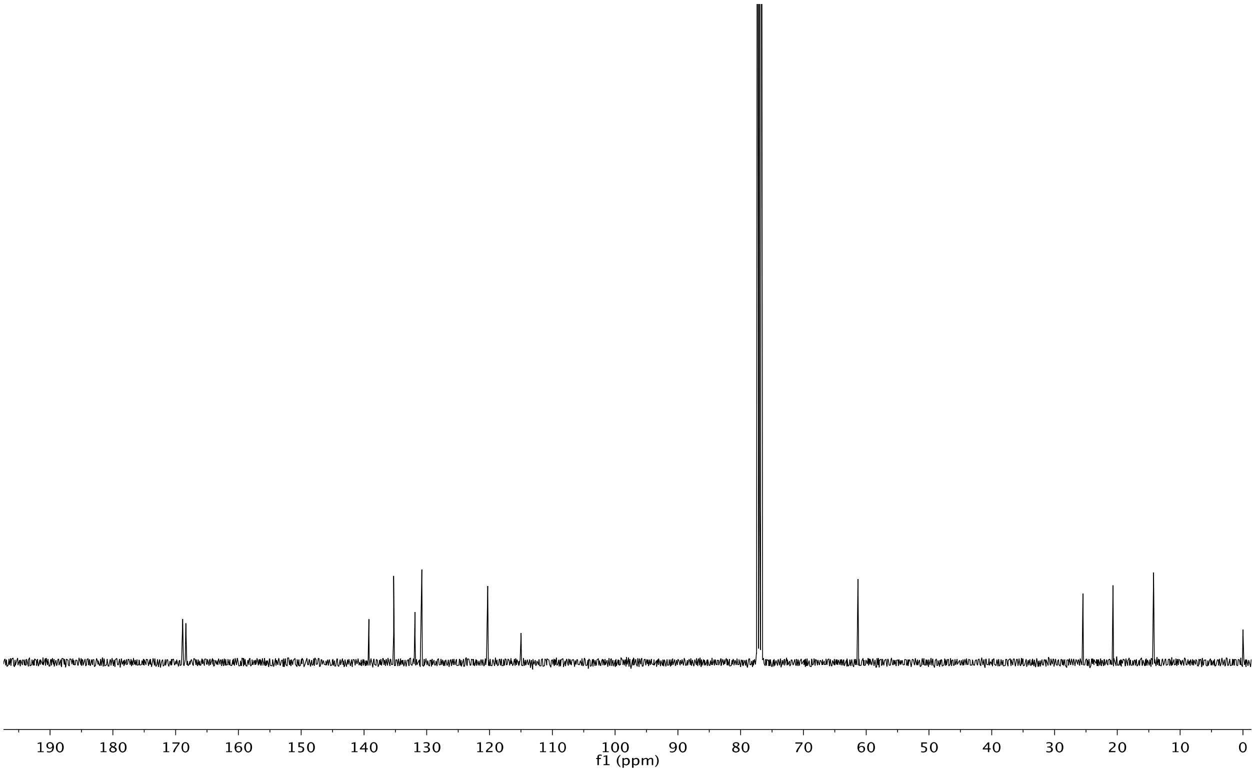

[1]H for *(±)-2-Ethoxy-2-ethyl-6-methyl-1,2-dihydro-4H-benzo[d][1,3]oxazin-4-one* (**18b**)

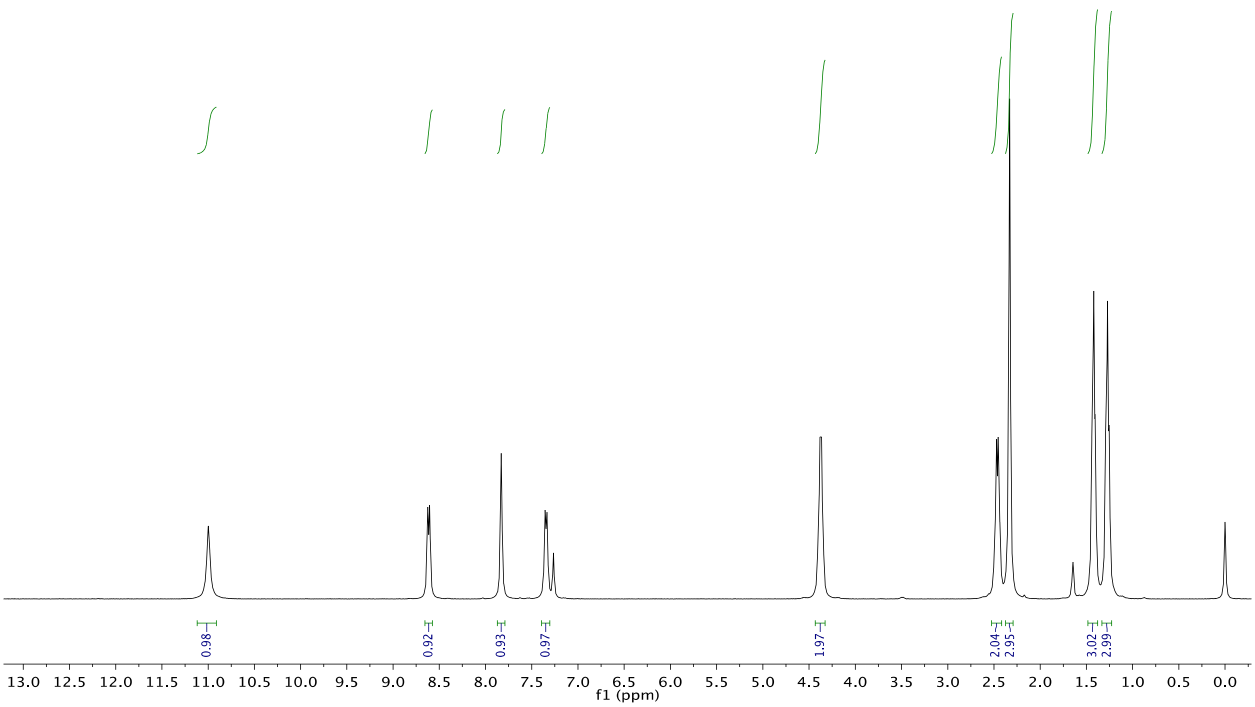

[13]C for *(±)-2-Ethoxy-2-ethyl-6-methyl-1,2-dihydro-4H-benzo[d][1,3]oxazin-4-one* (**18b**)

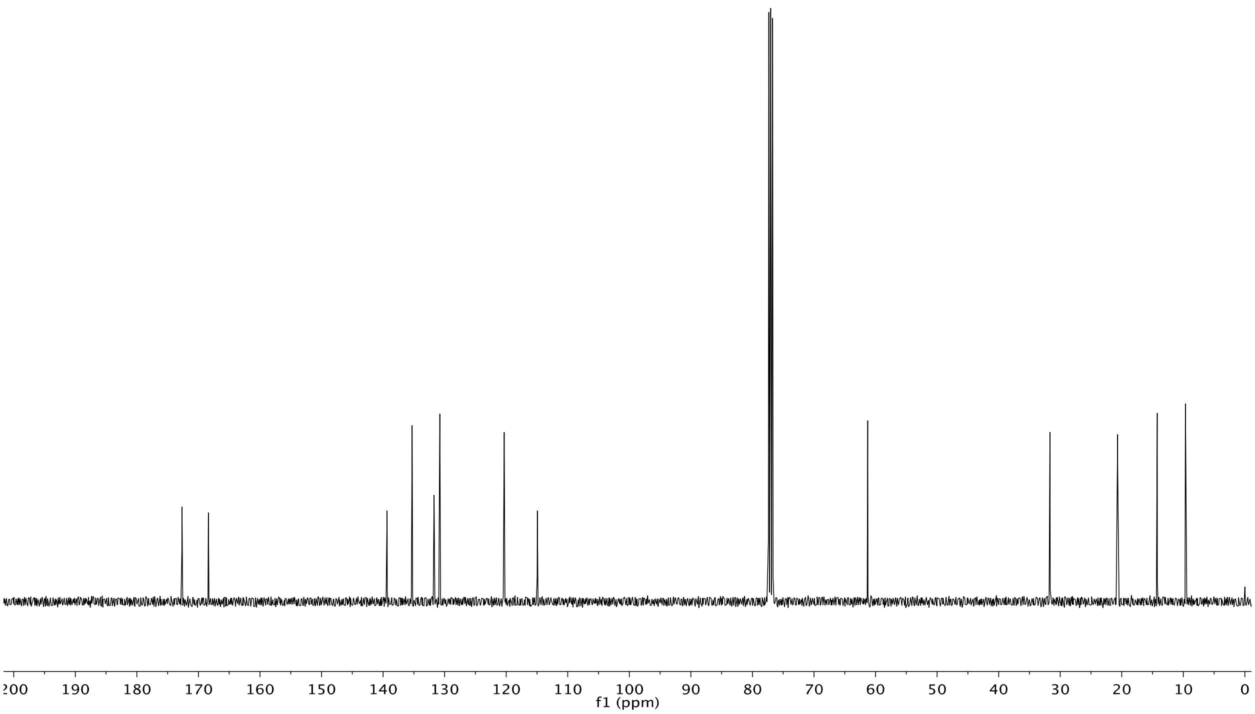

[1]H for *(±)-2-Ethoxy-6-methyl-2-propyl-1,2-dihydro-4H-benzo[d][1,3]oxazin-4-one* (**18c**)

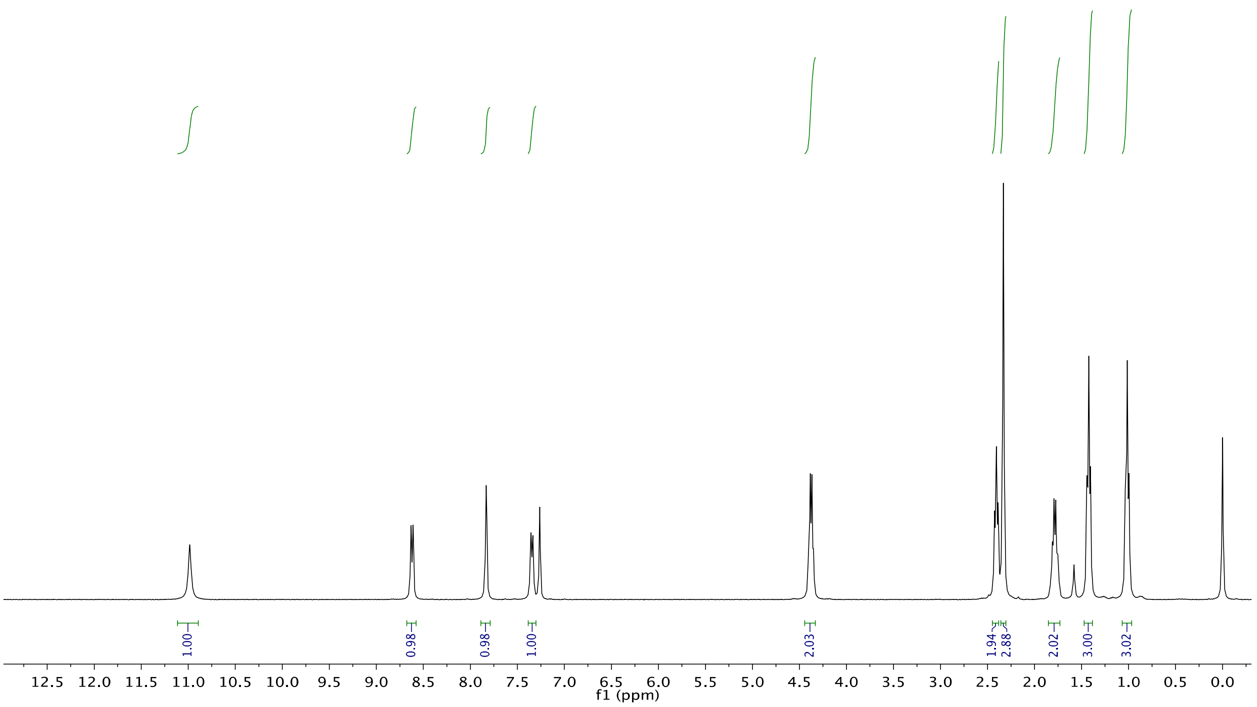

[13]C for *(±)-2-Ethoxy-6-methyl-2-propyl-1,2-dihydro-4H-benzo[d][1,3]oxazin-4-one* (**18c**)

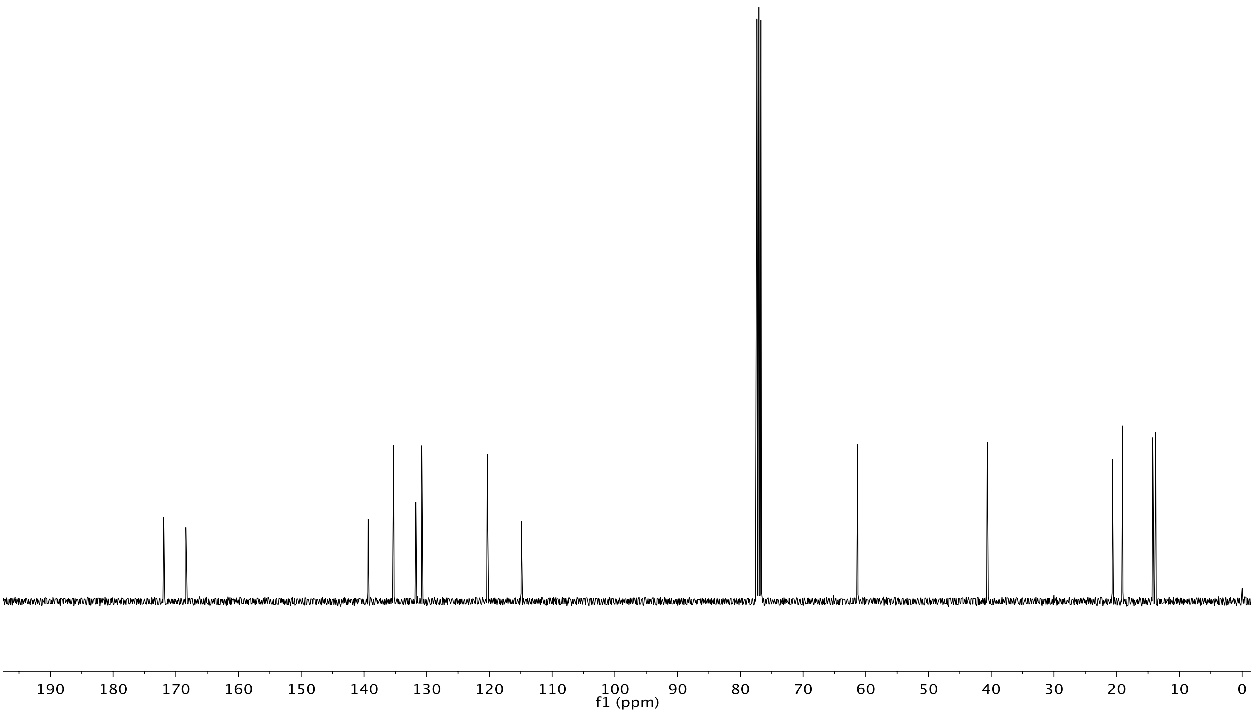

^{1}H for *(±)-2-Ethoxy-6-methyl-2-phenyl-1,2-dihydro-4H-benzo[d][1,3]oxazin-4-one* (**18d**)

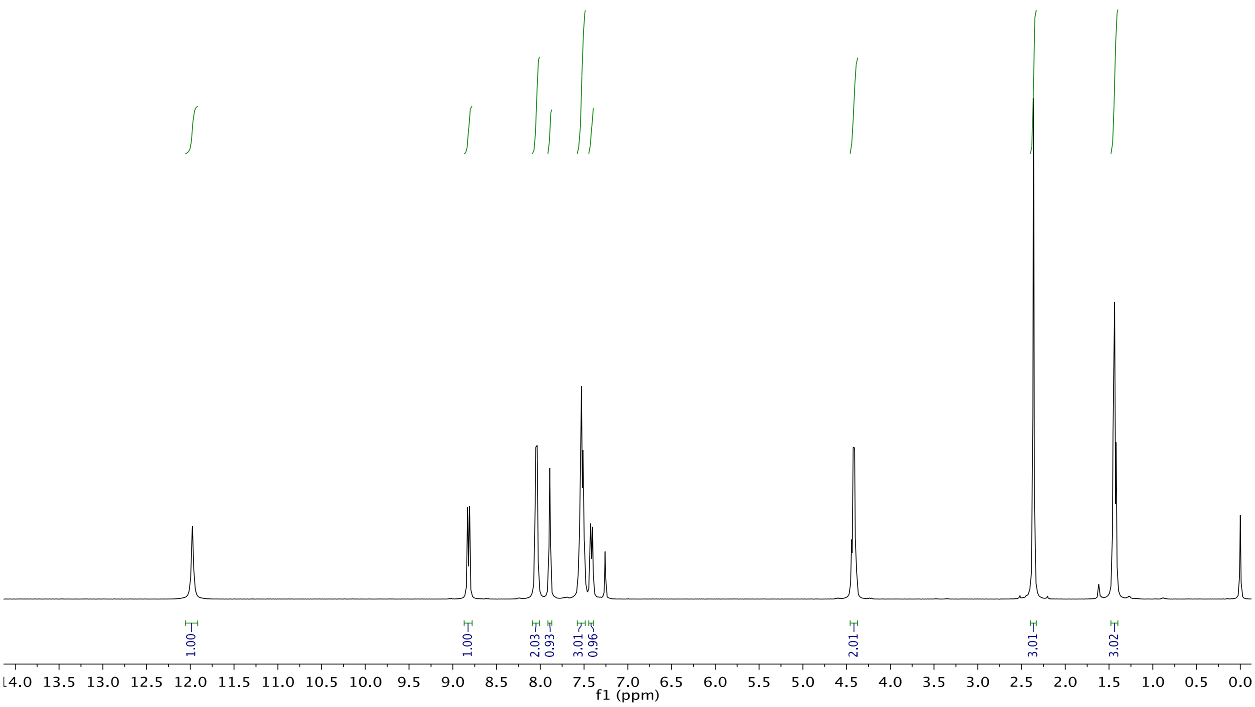

^{13}C for *(±)-2-Ethoxy-6-methyl-2-phenyl-1,2-dihydro-4H-benzo[d][1,3]oxazin-4-one* (**18d**)

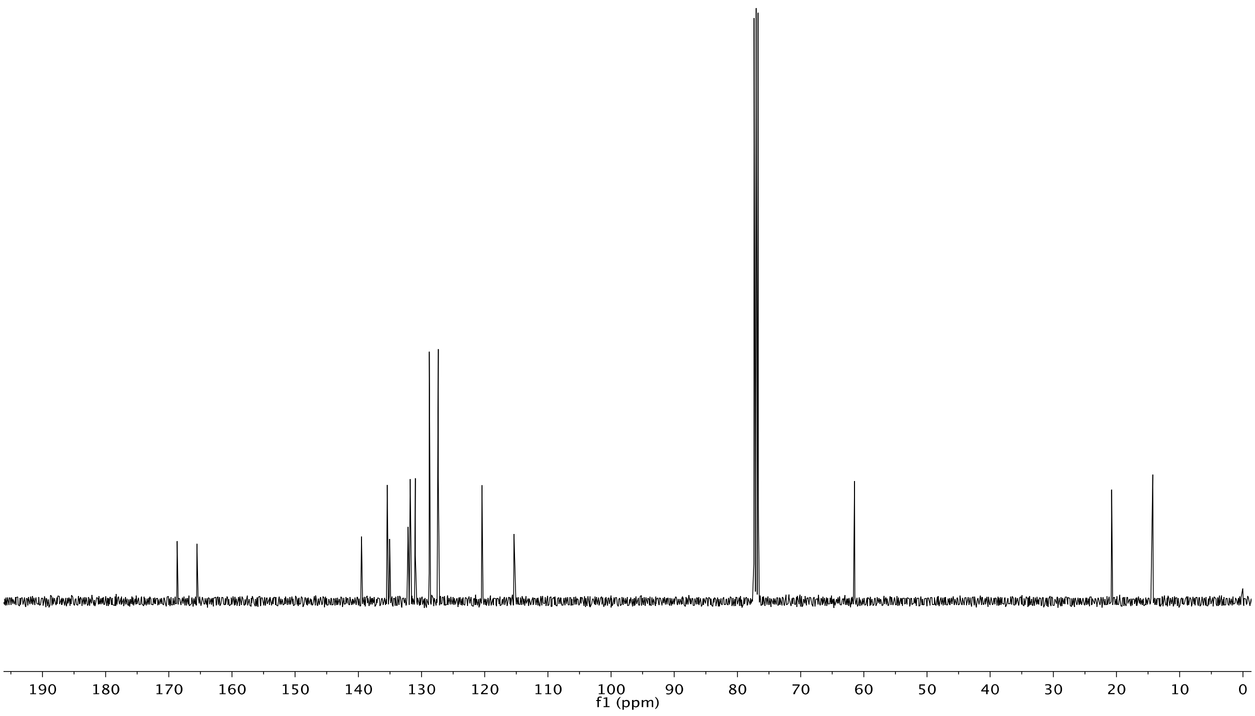

[1]H for *(±)-2-Ethoxy-2-methyl-7-nitro-1,2-dihydro-4H-benzo[d][1,3]oxazin-4-one* (**19a**)

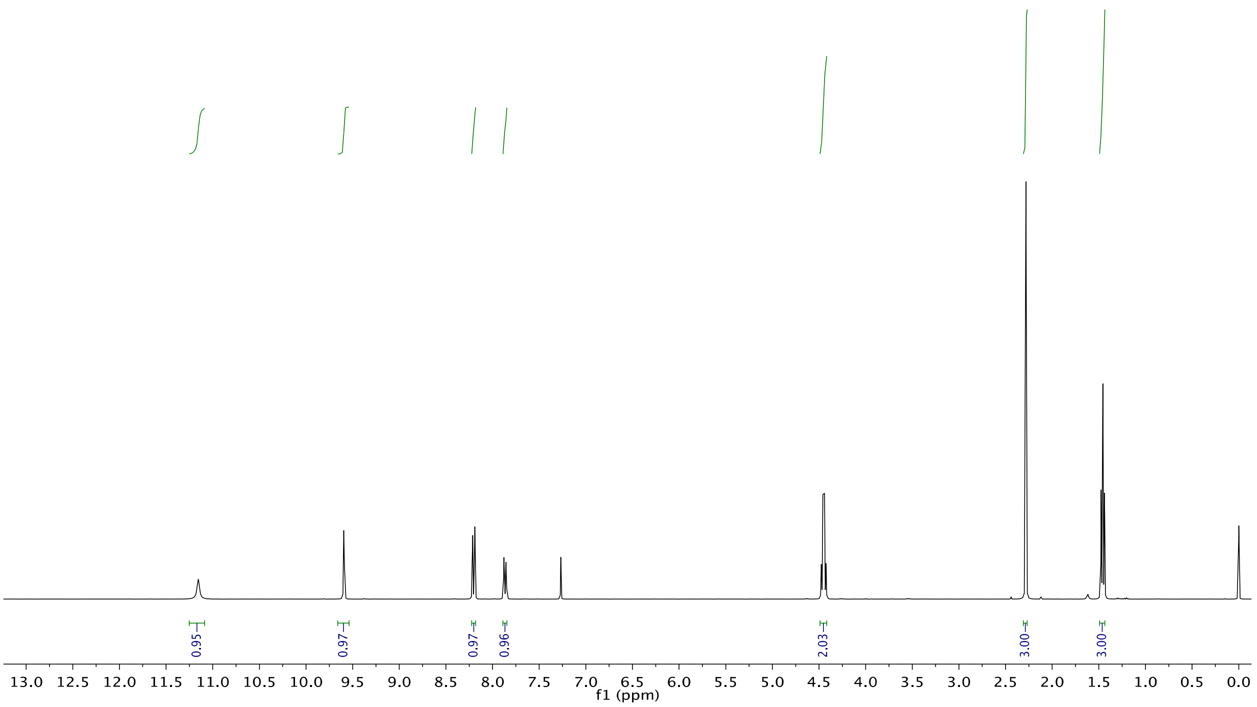

[13]C for *(±)-2-Ethoxy-2-methyl-7-nitro-1,2-dihydro-4H-benzo[d][1,3]oxazin-4-one* (**19a**)

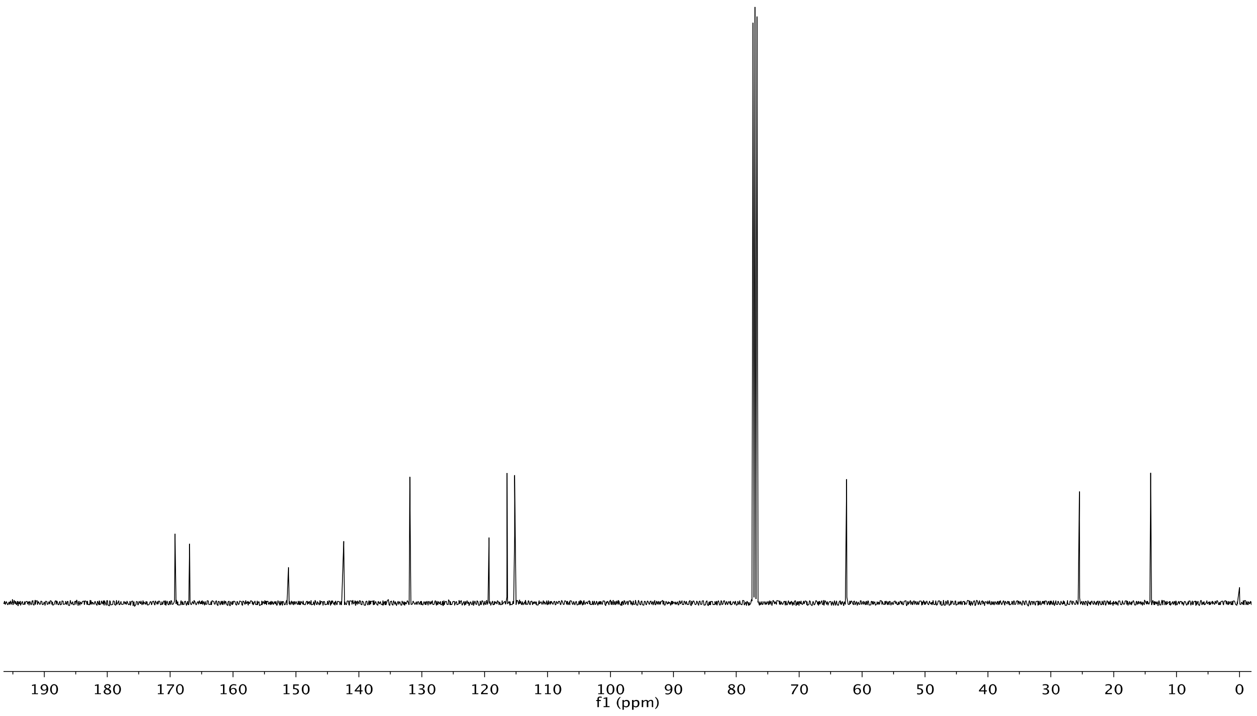

[1]H for *(±)-2-Ethoxy-2-ethyl-7-nitro-1,2-dihydro-4H-benzo[d][1,3]oxazin-4-one* (**19b**)

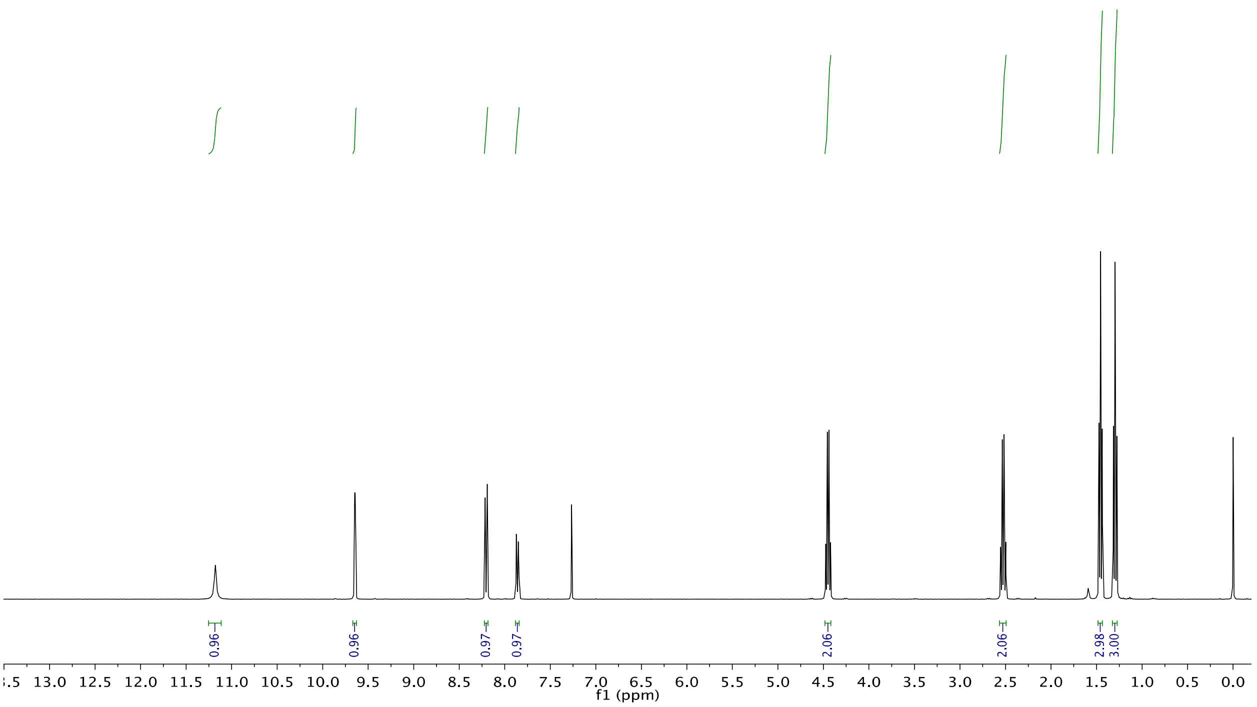

[13]C for *(±)-2-Ethoxy-2-ethyl-7-nitro-1,2-dihydro-4H-benzo[d][1,3]oxazin-4-one* (**19b**)

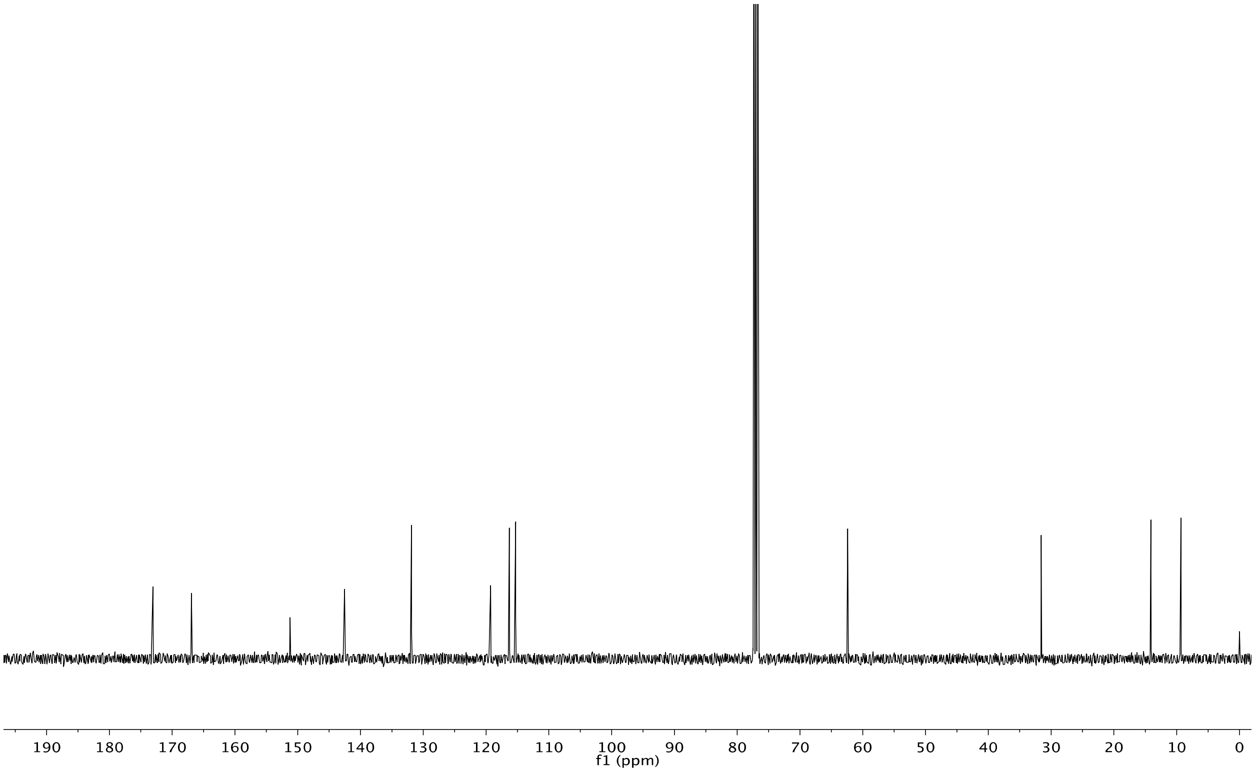

^{1}H for *(±)-2-Ethoxy-7-nitro-2-propyl-1,2-dihydro-4H-benzo[d][1,3]oxazin-4-one* (**19c**)

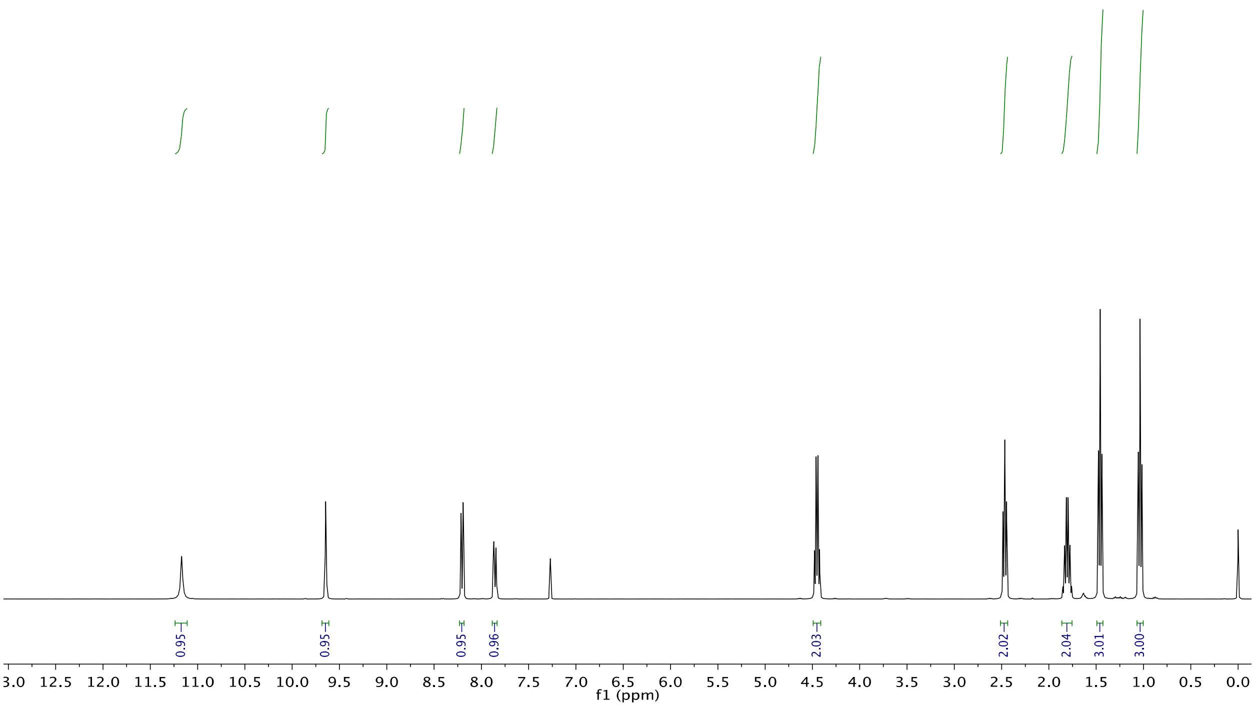

^{13}C for *(±)-2-Ethoxy-7-nitro-2-propyl-1,2-dihydro-4H-benzo[d][1,3]oxazin-4-one* (**19c**)

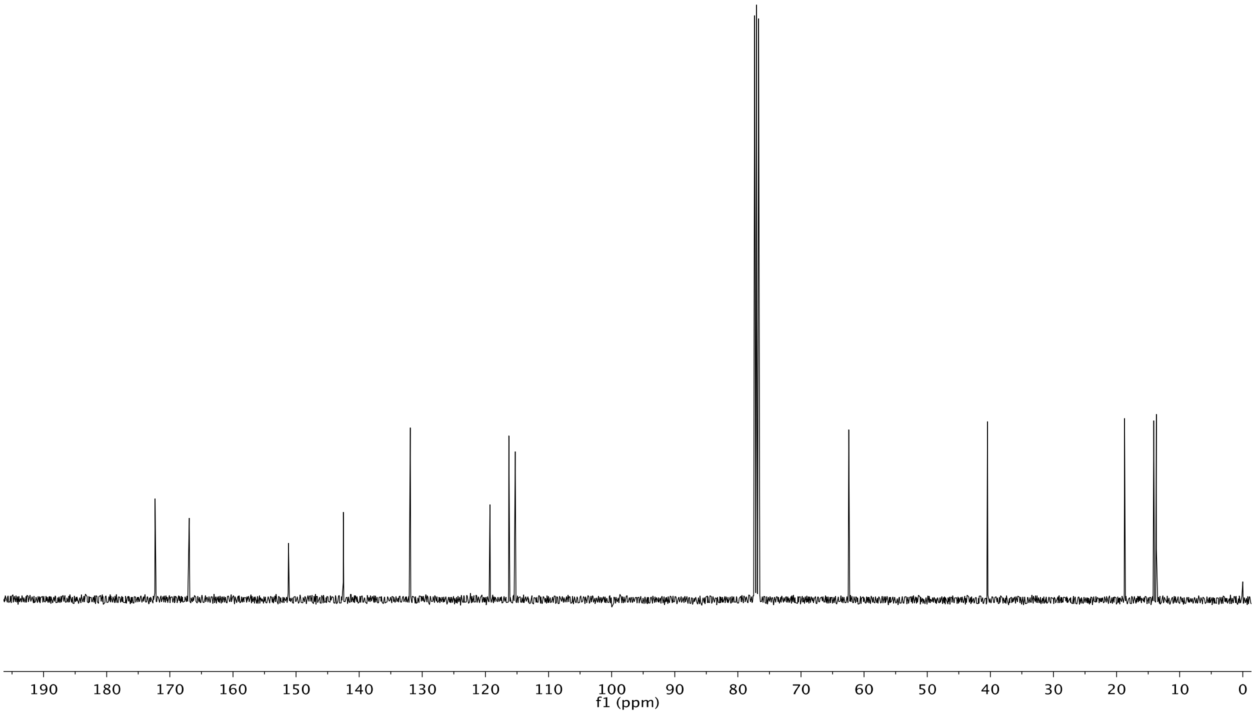

[1]H for *(±)-2-Ethoxy-7-nitro-2-phenyl-1,2-dihydro-4H-benzo[d][1,3]oxazin-4-one* (**19d**)

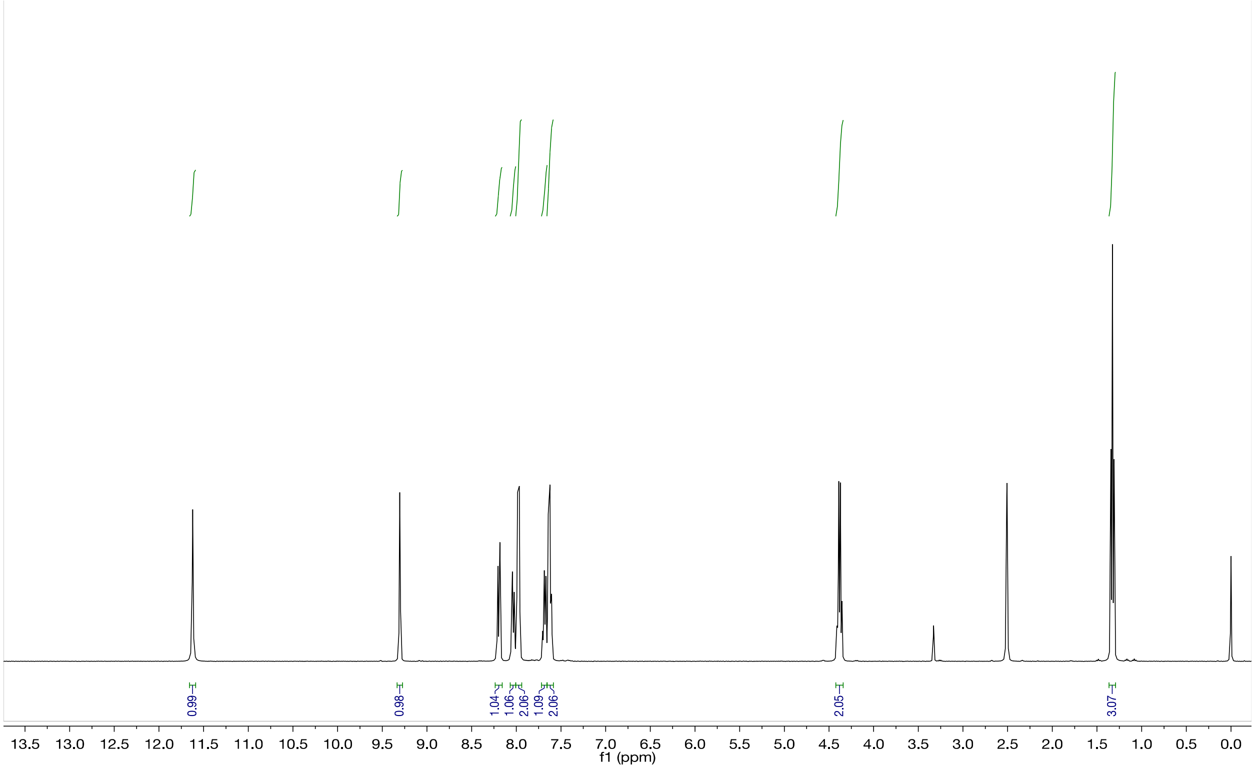

[13]C for *(±)-2-Ethoxy-7-nitro-2-phenyl-1,2-dihydro-4H-benzo[d][1,3]oxazin-4-one* (**19d**)

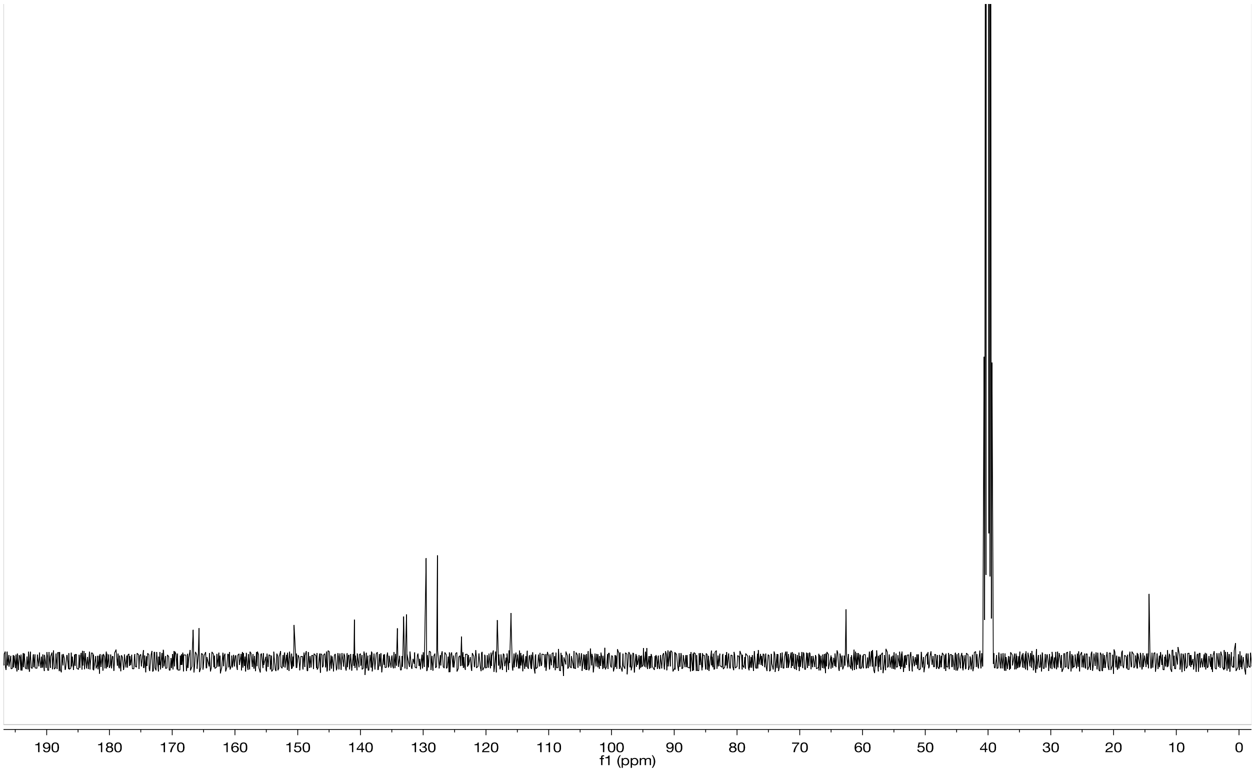

[1]H for *(±)-7-Chloro-2-ethoxy-2-methyl-1,2-dihydro-4H-benzo[d][1,3]oxazin-4-one* (**20a**)

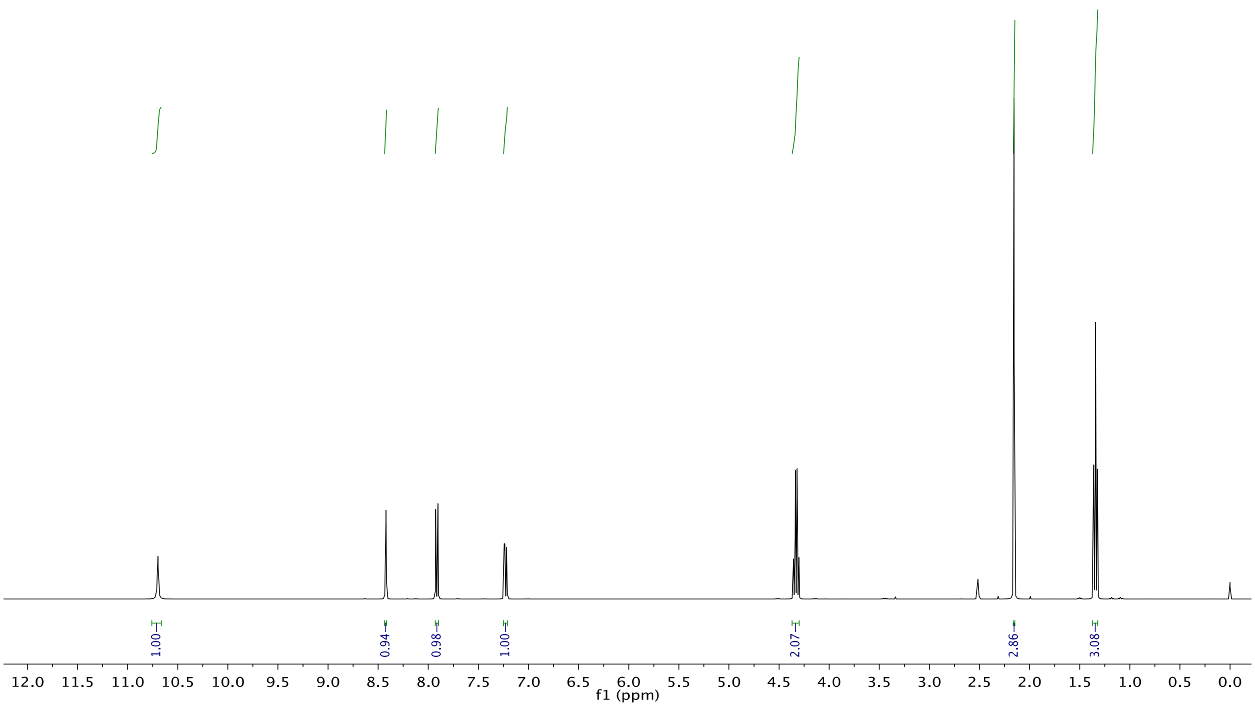

[13]C for *(±)-7-Chloro-2-ethoxy-2-methyl-1,2-dihydro-4H-benzo[d][1,3]oxazin-4-one* (**20a**)

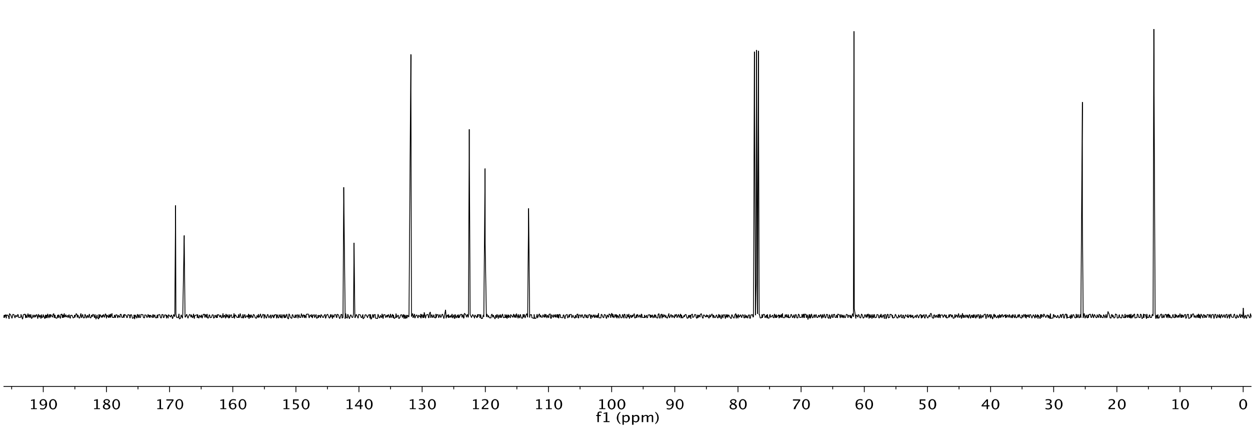

^{1}H for *(±)-7-Chloro-2-ethoxy-2-ethyl-1,2-dihydro-4H-benzo[d][1,3]oxazin-4-one* (**20b**)

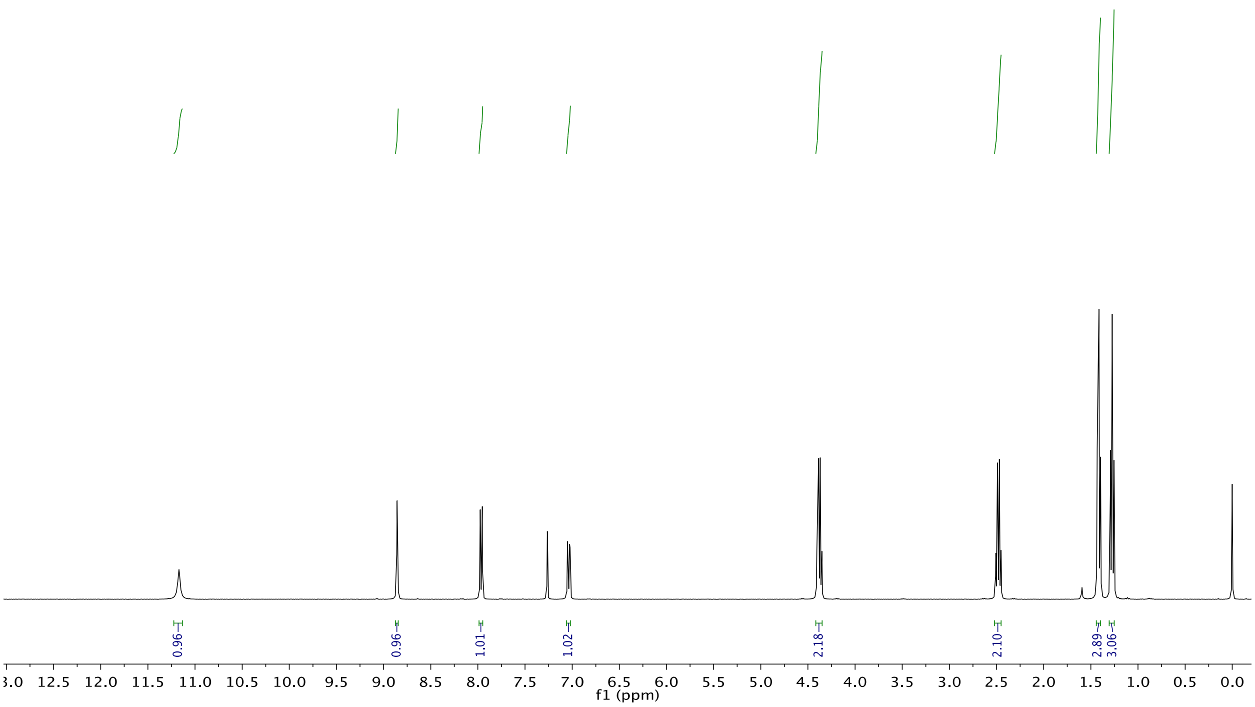

^{13}C for *(±)-7-Chloro-2-ethoxy-2-ethyl-1,2-dihydro-4H-benzo[d][1,3]oxazin-4-one* (**20b**)

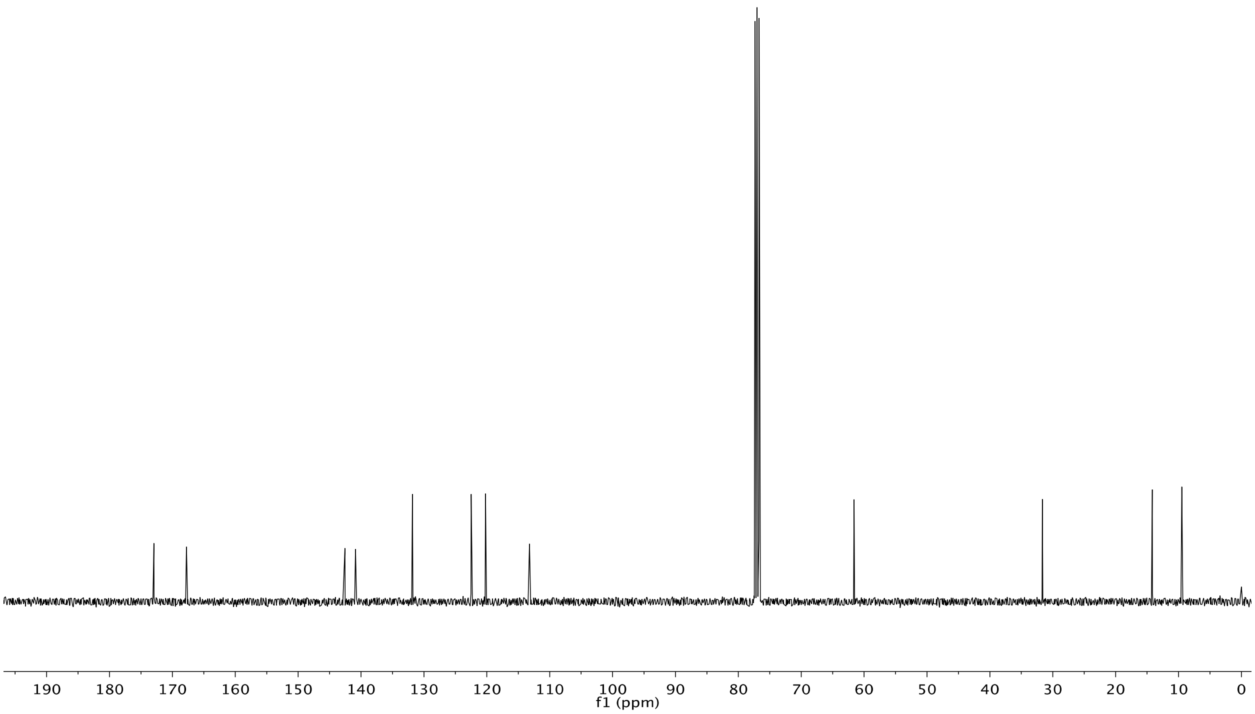

^{1}H for *(±)-7-Chloro-2-ethoxy-2-propyl-1,2-dihydro-4H-benzo[d][1,3]oxazin-4-one* (**20c**)

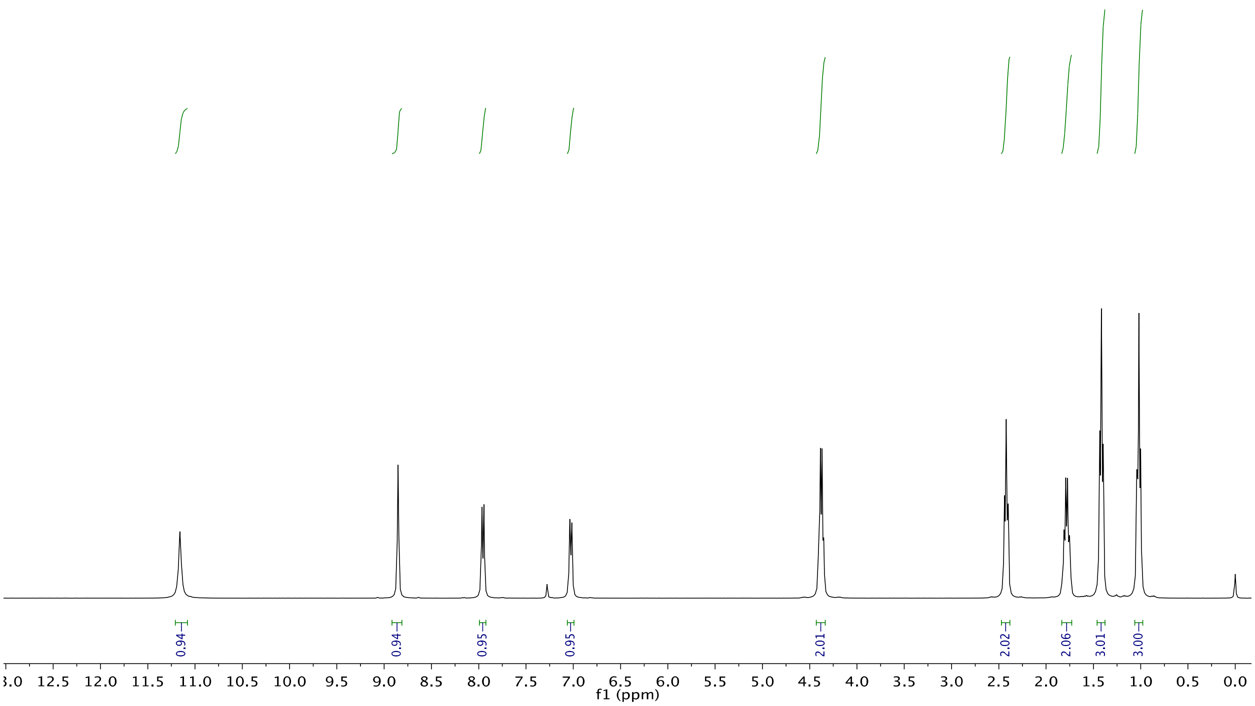

^{13}C for *(±)-7-Chloro-2-ethoxy-2-propyl-1,2-dihydro-4H-benzo[d][1,3]oxazin-4-one* (**20c**)

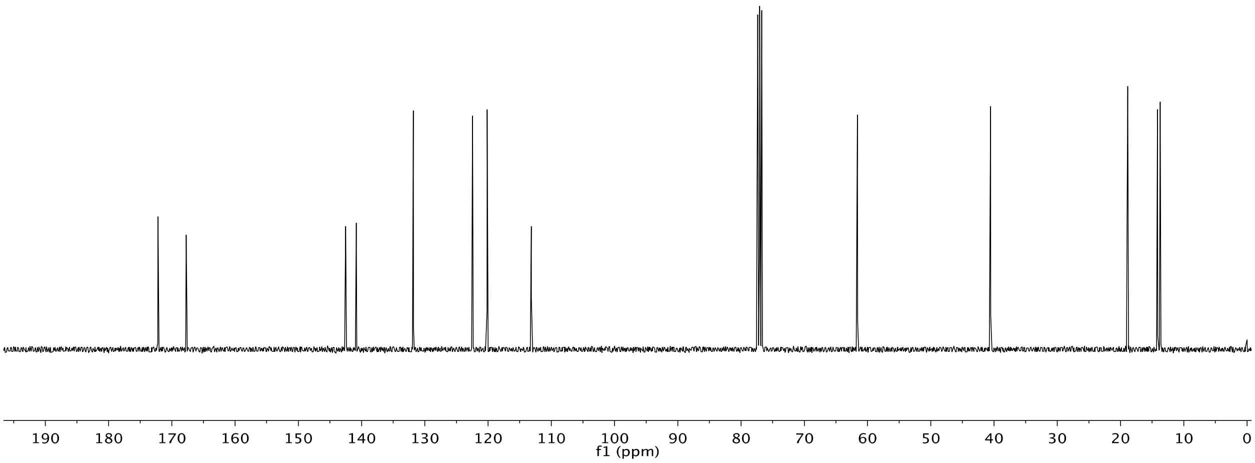

^{1}H for *(±)-7-Chloro-2-ethoxy-2-phenyl-1,2-dihydro-4H-benzo[d][1,3]oxazin-4-one* (**20d**)

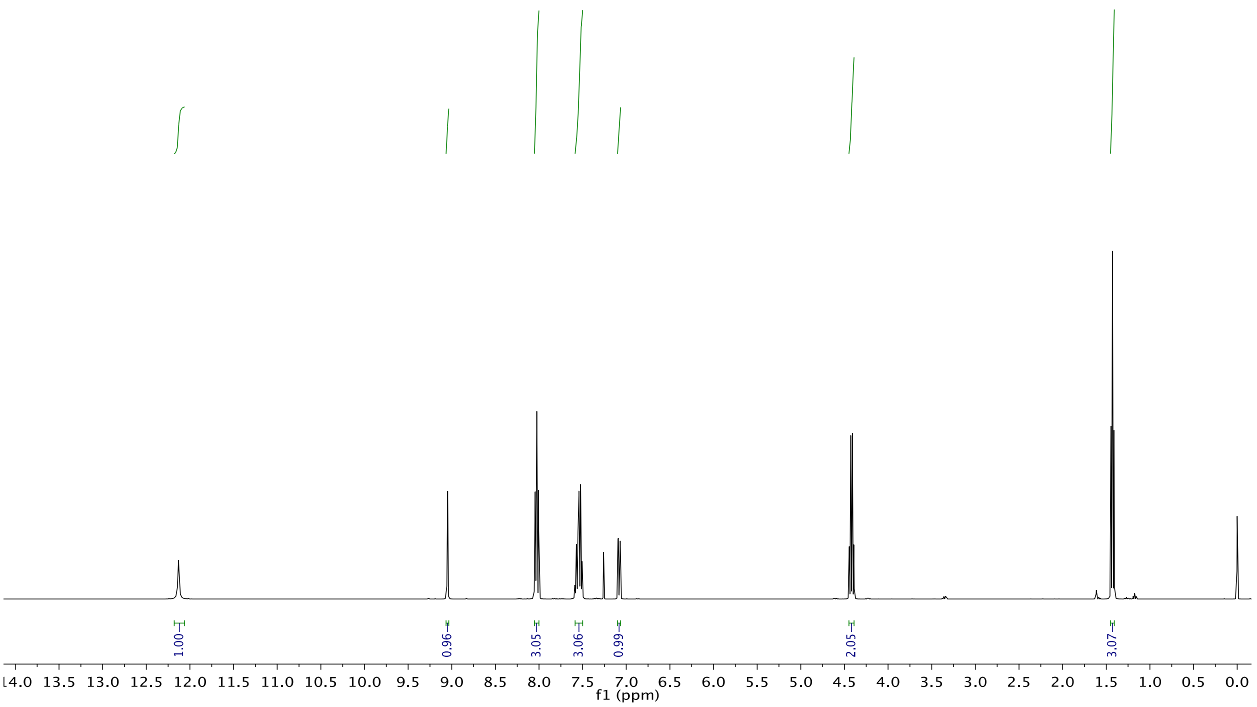

^{13}C for *(±)-7-Chloro-2-ethoxy-2-phenyl-1,2-dihydro-4H-benzo[d][1,3]oxazin-4-one* (**20d**)

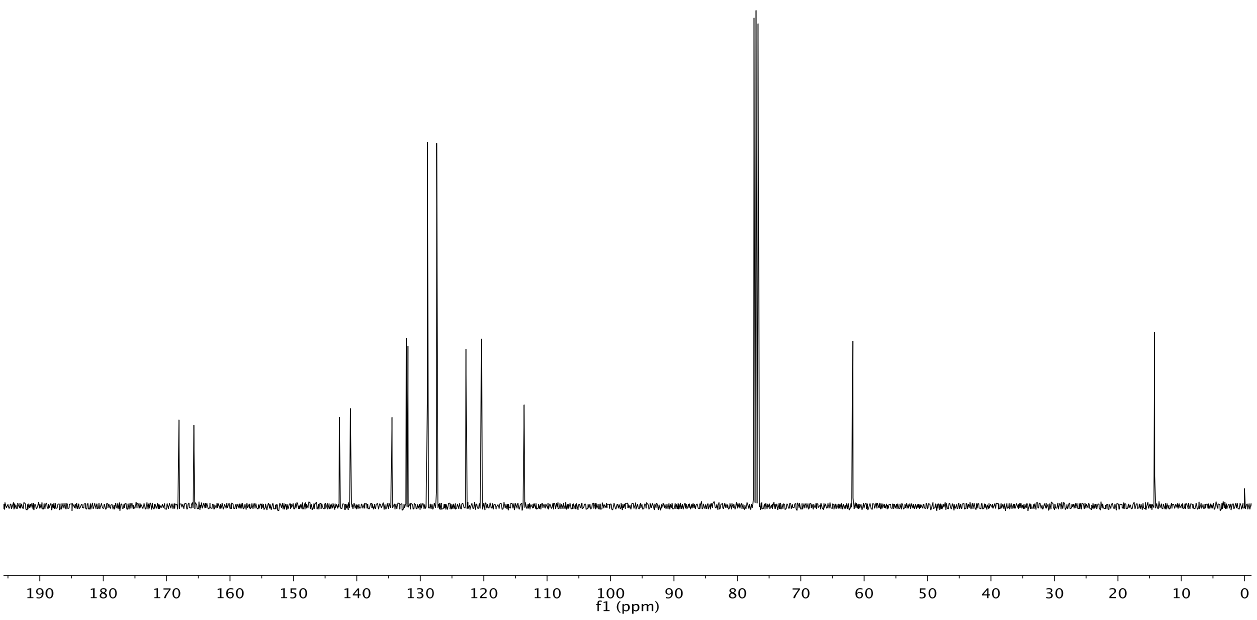

^{1}H for *(±)-2-Ethoxy-2-methyl-1,2-dihydro-4H-pyrido[2,3-d][1,3]oxazin-4-one* (**21a**)

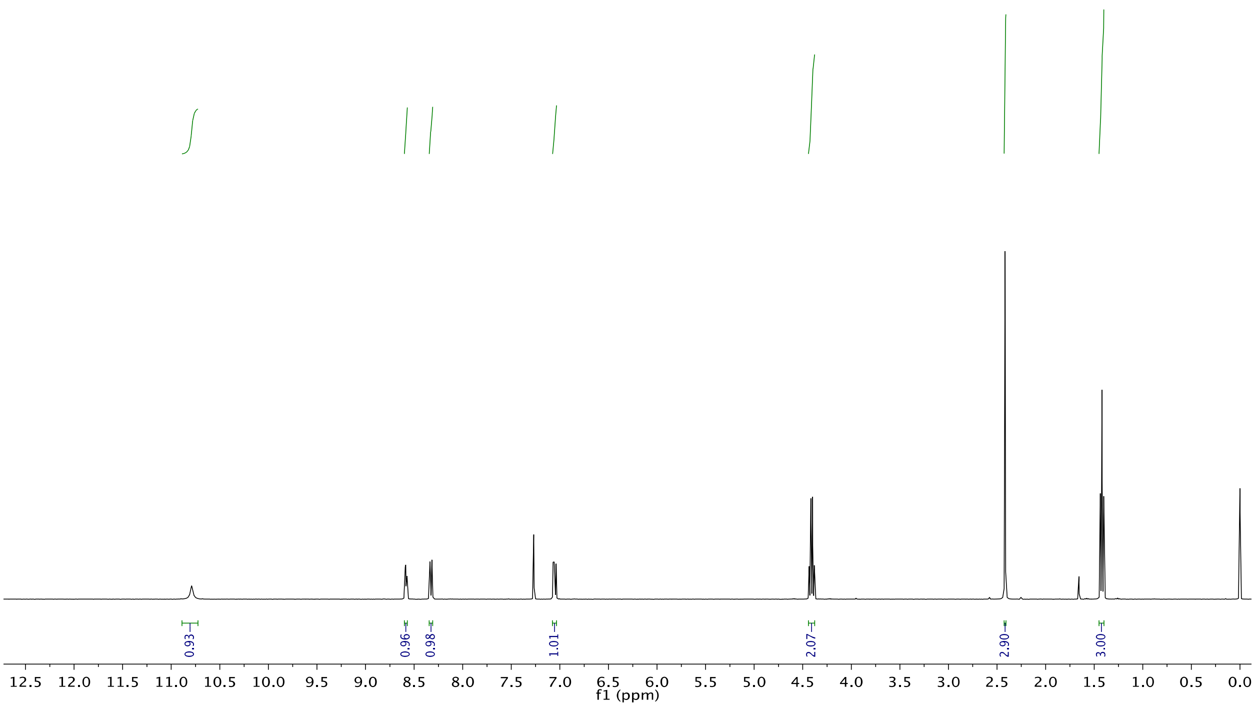

^{13}C for *(±)-2-Ethoxy-2-methyl-1,2-dihydro-4H-pyrido[2,3-d][1,3]oxazin-4-one* (**21a**)

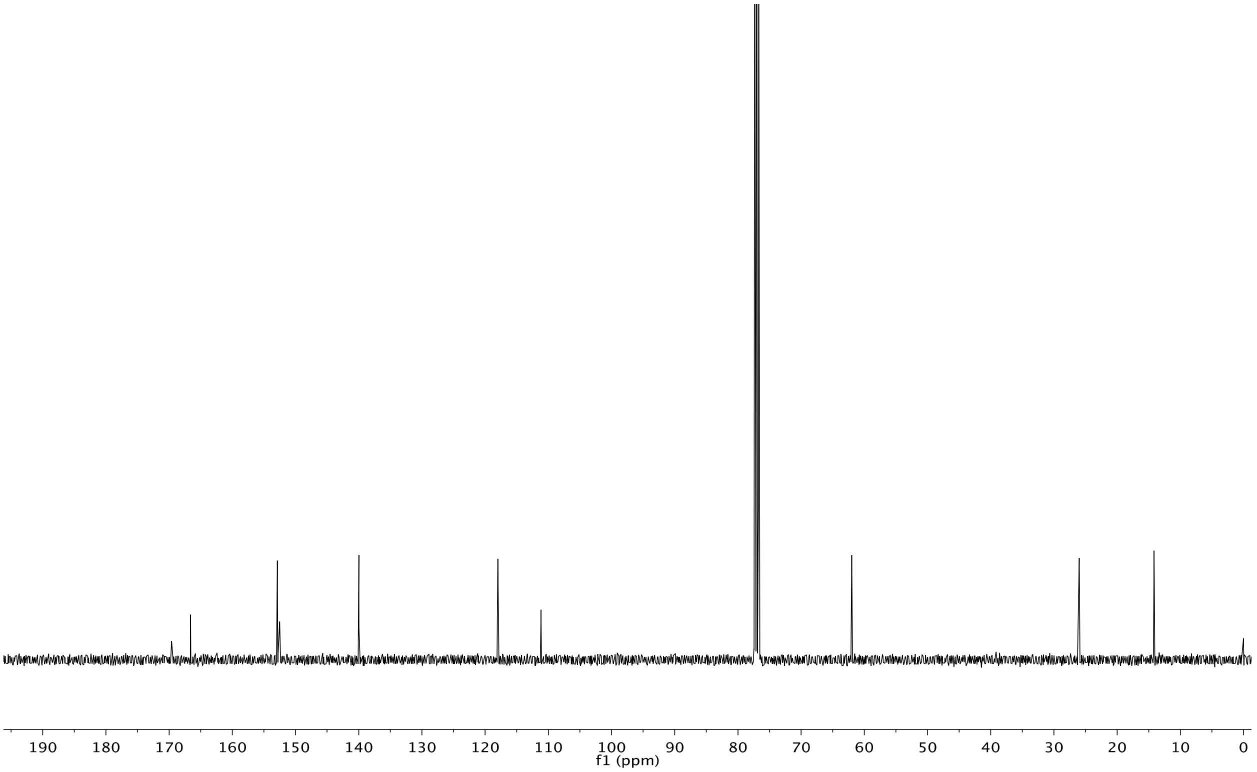

Supplement: Supplementary file 1 [file molecules-24-03555-s001.pdf]
